# Conversational linguistic features inform social-relational inference

**DOI:** 10.3758/s13423-025-02654-0

**Published:** 2025-03-06

**Authors:** Helen Schmidt, Sophia Tran, John D. Medaglia, Virginia Ulichney, William J. Mitchell, Chelsea Helion

**Affiliations:** 1 Department of Psychology and Neuroscience, Temple University, Philadelphia, PA USA; 2 Department of Psychological and Brain Sciences, Drexel University, Philadelphia, PA USA

**Keywords:** Social cognition, Judgment and decision making, Language

## Abstract

Whether it is the first day of school or a new job, individuals often find themselves in situations where they must learn the structure of existing social relationships. However, the mechanisms through which individuals evaluate the strength and nature of these existing relationships – social-relational inference – remain unclear. We posit that linguistic features of conversations may help individuals evaluate social relationships and may be associated with social-relational inference. Leveraging a naturalistic behavioral experiment (57 adults; 34,735 observations), participants watched a mid-season episode of a reality television show and evaluated the observed dyadic relationships between contestants. We employed novel person- and stimulus-focused approaches to: (1) investigate social-relational inference similarity between participants, (2) examine the association between distinct linguistic features and social-relational inference, and (3) explore the relationship between early season conversation similarity and later perceived relationship formation. We found high pairwise participant response similarity across two relational subtypes (friendship, rivalry), distinct associations between relational judgments and linguistic features, including semantic similarity, sentiment, and clout, and no evidence of an association between early conversation similarity and later friendship inference. These findings suggest that naturalistic conversational content is both a potential mechanism of social-relational inference and a promising avenue for future research.

**Supplementary Information:**

The online version contains supplementary material available at 10.3758/s13423-025-02654-0.

## Introduction

The first day at a new school or job can be remarkably challenging. Not only does one have to navigate a new building and adjust to new responsibilities, but one must also learn about new relationships and complex social structures (Basyouni & Parkinson, 2022). Success in learning new social structures is critically important for health and well-being, as an individual’s position and linkage within a social network has been associated with both positive (e.g., prosocial behavior, physical and mental health) (Bond et al., 2012; van den Bos et al., 2018) and negative (e.g., propensity for depression, tobacco use) (Ennett & Bauman, 1994; Rosenquist et al., 2011) outcomes. Moreover, having a more accurate representation of one’s network is associated with better social outcomes (Yu & Kilduff, 2020) and positively impacts professional and academic performance (Lee et al., 2017; Marineau, 2017). Despite the importance of successfully learning about the strength and valence of social relationships, what we term *social-relational inference*, to date, relatively little is known about how we learn and represent these relational associations dynamically (Tompson et al., 2019). It is also unclear how conversations, a foundational form of social connection between individuals, inform social-relational inference.

The majority of research related to social learning has aimed to either (1) identify the consequences of accurate knowledge of existing social network positions and structures (Alt et al., 2022; Mobasseri et al., 2022; Yu & Kilduff, 2020), or (2) examine how individuals learn experimenter-generated networks that manipulate specific network features (Son et al., 2021; Tompson et al., 2020). This research builds upon these two areas by examining how individuals learn and represent specific dyadic relationships – rather than social network properties – using passive observation of naturalistic stimuli (Grall & Finn, 2022; Nastase et al., 2020). By naturalistic stimuli, we refer to stimuli that involve the integration of multimodal features, are immersive, contain dynamic, temporal components, and more closely approximate the complexity of real life (Stasiak et al., 2023). We focus on passive observation given its importance in relational learning across species (Lee et al., 2024; Seyfarth & Cheney, 2013) and in early development (Hamlin et al., 2007). Moreover, it is likely one of the key modalities through which novel social information is learned in the real world.

In this article, we examine how individuals make inferences about two types of dyadic associations – relational and non-relational – in the context of a competitive game. Relational inferences (Liberman & Shaw, 2018) refer to evaluating if observed dyads are friends or rivals; this association is the basis for a social network (Alt et al., 2022; Baek et al., 2021). Non-relational inferences refer to examining who in an observed dyad is more likely to beat the other person to win the game. While both are social in nature, the former involves integrating information about dyadic relationships while *also* focusing on the nature of their interactions with each other.

The approach in the present research allows us to gain more nuanced knowledge of what specific factors are associated with social-relational inference. Prior work has found that multidimensional features of interpersonal interactions, including personality, emotion, and verbal content, provide information about social relationships (Alt et al., 2022; Son et al., 2021; Tong et al., 2020). Recently, psychologists have argued that linguistic analysis is a tool that should be used more frequently to study the underlying psychological mechanisms behind interpersonal interactions (Boyd & Schwartz, 2021; Jackson et al., 2021). This approach may help to close a meaningful knowledge gap in how features from communicative channels *specifically* contribute to social-relational inference. There is reason to suspect they might play a large role. People who tell secrets to each other are more likely to be perceived as friends (Liberman & Shaw, 2018), and differences in linguistic styles can reliably reflect personality characteristics (Slatcher et al., 2007). Verbal cues can indicate friendly or hostile attitudes between speakers (Argyle et al., 1971). Additionally, verbal indicators of positive emotions can evoke signals of trustworthiness (Anderson & Thompson, 2004) and are predictive of more enjoyable social interactions (Berry & Hansen, 1996) and closer friendships (Berry et al., 2000). Lastly, verbal content has been shown to be the best predictor of perceived conversational affect relative to non-verbal and tonal cues (Krauss et al., 1981).

While many linguistic features have psychological relevance (Cambria et al., 2017; Zhang et al., 2018), we focus primarily on linguistic similarity. Other work has identified that this feature may be particularly important for social-relational inference (Kovacs & Kleinbaum, 2020). For example, couples who speak more similarly to each other early in their relationships are more likely to be together in the long term (Ireland et al., 2011), and language style-matching and positive emotion words are seen in supportive conversations with friends (Cannava & Bodie, 2017). Dyadic conversations between strangers also exhibit style-matching, that is, conversations that are more stylistically similar (Babcock et al., 2014). As such, we hypothesize that greater dyadic verbal semantic similarity would be associated with an increased likelihood of perceived friendship (and a reduced likelihood of perceived rivalry).

We also examine the association between linguistic similarity and relational homophily (i.e., the prediction that similar individuals are more likely to be friends) (McPherson et al., 2001). Prior work suggests that friends are more similar to each other (Bahns et al., 2017) and individuals perceive and respond to information similarly to their friends (Parkinson et al., 2018). However, it is unclear if *linguistic* similarity precedes or results from friendship; in other words, are individuals who speak similarly more likely to become friends or do individuals speak more similarly to their existing friends? Consistent with prior work, we predict that early linguistic similarity between individuals will be associated with a greater likelihood of being perceived as friends later.

In the present research, participants took part in a naturalistic behavioral experiment wherein they passively observed contestant interactions and evaluated dyadic relationships while watching a mid-season episode of a reality television show. Across three analytic aims, we explored whether passive observation of these interactions was associated with similar patterns of social-relational inference, whether distinct linguistic features of contestant conversations – in particular, semantic similarity – were associated with social-relational inference, and whether early conversational similarity was associated with later relational judgments.

## Social-relational inference similarity

### Method

#### Participants

Fifty-seven participants were recruited from a large city in the northeastern USA. Informed consent was obtained for all participants, and participants received course credit for their time. Experimental procedures were approved by the university’s Institutional Review Board. Demographic information for the first ten participants was not collected due to an administrative error, but information was collected for all subsequent participants (45 female; two other; 23 non-White; *M*_*age*_ = 19.08 years, *SD*_*age*_ ± 1.48 years). The goal sample size was 50 participants due to time and resource constraints, but due to oversampling, data from 57 participants was collected (Brysbaert & Stevens, 2018). Two participants missed responses to 50 or more trials, but this did not greatly impact the total number of observations collected (*N*_*observations*_ = 34,735, *M*_*participant*_ = 609.4 responses, *SD*_*responses*_ ± 19.7 responses), so they were included in the final sample.^1^ Participants were verbally screened about their familiarity with the reality show season and episode, and none reported being familiar with it.

#### Procedure

Participants took part in an experiment wherein they learned about the nature and strength of novel social relationships via passive observation of a mid-season television episode of *Survivor* (CBS Television). *Survivor* is a particularly fitting stimulus to examine these questions – it captures unscripted, real-life conversations between individuals in a context where the goal is forming different types of relationships (i.e., forming alliances and rivalries) to win the game. We selected a mid-season episode that came right after previously separated teams merged, ensuring a mix of alliances and rivalries.

**Fig. 1:**
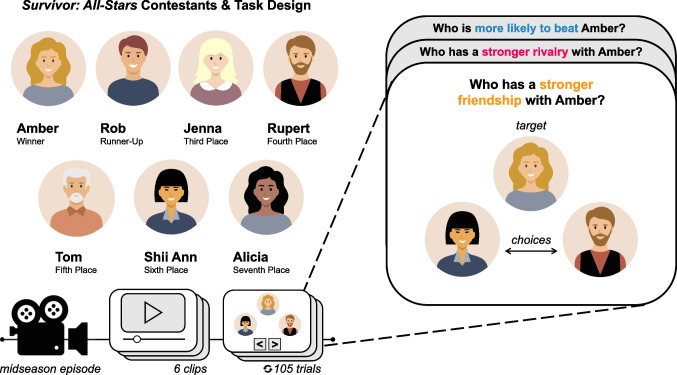
Overview of task design. Participants observed seven contestants interacting with each other on a mid-season episode of *Survivor* and made binary decisions about the extent to which contestants were friends with, rivals with, or more likely to win the season over other contestants

Participants watched a full episode from the television show *Survivor* (Season 8, Episode 13; aired 22 April 2004, CBS Television), which was divided into six clips (*M*_*length*_ = 6.50 min, *SD*_*length*_ ± 0.03 min). In a within-subjects design, participants watched each clip with closed captioning in chronological order and made a series of keyboard responses after each clip finished playing. The task was designed and implemented using PsychoPy (Peirce et al., 2019). Each episode clip and subsequent keyboard responses comprised one experimental block, with each block falling into one of three decision conditions: friendship, rivalry, or win (Fig. 1). Participants completed each decision condition twice, and decision blocks were presented in a random order.

When making responses, participants were presented with three photos of contestants from the episode. One photo, displayed at the top of the screen, was the “target,” and the other two photos, displayed below the target photo, were possible “choices.” Above the target photo, a question was displayed that dynamically changed depending on the decision condition. For friendship blocks, participants were asked “Who has a stronger friendship with X?”, where X was the name of the contestant in the target photo. In rivalry blocks, participants were asked “Who has a stronger rivalry with X?” and in win blocks, participants were asked “Who is more likely to beat X?” Participants selected one of the two choice contestants per trial using the keyboard. Once a response was made, a new target photo and two new choices were displayed. Participants repeated these responses for every possible pairwise combination of contestants (105 possible trials per block), and they had up to 5 s to make a response.

#### Intersubject correlation

To investigate if passive observation of contestant interactions was an effective means to evaluate the nature and strength of relationships, we first examined the similarity of participant responses across each condition (friend, rival, win). We used intersubject correlation (ISC) – a computational approach that explores shared information representation using pairwise correlations – to examine whether participants represented social-relational information similarly to one another (Nastase et al., 2019; Smith et al., 2020). Using the “tidyverse” (Wickham et al., 2019) suite of packages in R, we calculated the proportion of time participants chose each dyad (i.e., pair of contestants) as friends, rivals, or winners when presented during a given trial. For example, if during the friendship block a participant selected Amber 80% of the time that she was presented as a choice when Rob was the target and also selected Rob 60% of the time that *he* was presented as a choice when Amber was the target, this participant’s average friendship proportion for the Amber-Rob dyad would be 70%. Friend and rival responses were aggregated regardless of which contestant was shown as the target or choice option to evaluate the dyadic relationship, while win responses were kept directional. Due to the nature of the question presented to participants, the win response is an evaluation of which choice option will win *instead* of the target, while friend and rival responses evaluate the relational strength *between* the target and selected choice. Moreover, because there are two response blocks for each decision condition, the calculated proportions of time chosen for each dyad incorporate trials from both blocks. Throughout the article, these proportions of time chosen are referred to as *percent chosen* to reflect within-participant judgments and *response similarity* to reflect between-participant agreement.

To calculate response similarity across each condition (i.e., friendship, rivalry, win likelihood), we used the “widyr” package in R (Robinson & Silge, 2022) to calculate pairwise Pearson correlations between complete participant responses (i.e., participant-level percent chosen for each contestant dyad). Pairwise correlations ranged from − 1 (completely dissimilar responses) to 1 (completely similar responses). We calculated a median pairwise similarity value for each condition using the lower triangle of each response similarity matrix (Chen et al., 2016).

We compared each condition’s median pairwise response similarity score to a null distribution using bootstrapping. Across 10,000 iterations, we shuffled and randomly sampled with replacement from participant-level mean percent chosen values (i.e., participant A selected Amber and Rob as friends 80% of the time, on average) to calculate new participant by participant pairwise correlation matrices. This was performed separately for each condition. For each iteration, we calculated a median pairwise similarity value from the correlation matrix, and we examined the observed median for each condition to the distribution of 10,000 sampled pairwise similarity values. This comparison between the observed median and distribution of medians was then used to assess significance of the observed value (i.e., what proportion of bootstrapped medians fell at or above the observed value). See Online Supplemental Material for distributions of bootstrapped medians for each condition.

### Results

**Fig. 2:**
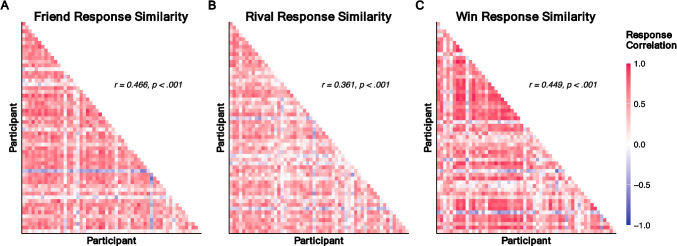
Response similarity across conditions. Response similarity matrices show each participant plotted along both axes, and the colored tile reflects each participant-by-participant pairwise correlation. Red values denote more similar responses while blue values denote less similar responses. Median pairwise similarity correlation (*r*) and significance (*p*) values reported for each condition. (**A**) Similarity matrix of friendship responses. (**B**) Similarity matrix of rivalry responses. (**C**) Similarity matrix of win responses

We found that participants made similar evaluations about contestant relationships across all three conditions (Fig. 2). Friendship responses were the most similar amongst participants (*r*_*friend*_ = 0.466, *SD*_*friend*_ = 0.282, *p* < 0.001), followed closely by win (*r*_*win*_ = 0.449, *SD*_*win*_ = 0.333, *p* < 0.001) and rivalry responses (*r*_*rival*_ = 0.361, *SD*_*rival*_ = 0.259, *p* < 0.001).

### Discussion

These results suggest that participants represented the nature and strength of observed contestant relationships similarly to one another across both relational (friend, rival) and non-relational (win) judgments. This indicates that individuals may be gleaning important and similar information from the observed content. In the next section, we examine one potential source of that information: the linguistic information conveyed by contestants in the episode.

## Conversational linguistic features

### Method

We test the hypothesis that greater dyadic semantic similarity is associated with social-relational inference, consistent with prior research that suggests greater conversational similarity is predictive of longer-term friendships and relationships (Cannava & Bodie, 2017; Ireland et al., 2011). In comparative analyses, we also test the extent to which two other linguistic features – sentiment, or the affective tone of a verbal exchange (Lindquist et al., 2015; Xie et al., 2021), and clout, or the confidence with which verbal information is conveyed (Kacewicz et al., 2014) – are associated with social-relational inference. We focus on sentiment and clout to capture specific emotional components of language that are both distinct from semantic similarity and closely relate to social dynamics within relational contexts. In addition to examining three distinct linguistic features, we also examine social-relational inference at four levels of analysis to examine how social dynamics and observer insights vary with time and dyadic involvement.

#### Episode transcription

Two independent coders transcribed dialogue from the *Survivor* episode, noting all spoken dialogue, the speaker’s name, the recipient’s name (to whom the speaker is talking), and block number (1–6). The transcriptions were further organized at the sentence-level, such that each sentence had a corresponding speaker name, recipient name, and block number. In all analyses, only contestants (Amber, Alicia, Jenna, Rob, Rupert, Shii Ann, and Tom) were included as speakers and no dialogue spoken by the host (Jeff Probst) was included.

#### Natural language processing

To evaluate how verbal information from conversations between *Survivor* contestants may inform social-relational judgments made by participants, we employed natural language processing techniques to quantify three distinct linguistic features. This included our primary linguistic metric of interest – semantic similarity – and two additional comparative exploratory features – sentiment and clout.

##### Semantic similarity

Semantic similarity was calculated using Google’s pre-trained Universal Sentence Encoder (USE-4), available on TensorFlow Hub and implemented in Python (Cer et al., 2018). This dimensional analysis measures to what extent two pieces of text have the same meaning using text embeddings, which is an advancement from prior work that investigates to what extent pieces of text use similar words or have similar styles (Kovacs & Kleinbaum, 2020). USE converts text into vectors in high-dimensional space (512 dimensions). USE is capable of calculating embeddings for sentences and paragraphs of text; our text inputs range from one to 283 words, which is within the range that USE-4 is capable of handling. We calculated the cosine similarity (i.e., the cosine of the angle between two text vectors) using “scikit-learn” (Pedregosa et al., 2011) to assess the similarity of two pieces of text. Cosine similarity values can range from − 1 (perfectly dissimilar) to 1 (perfectly similar), but most values in this article range from 0 to 1.

##### Sentiment 

Using the “sentimentr” package in R (Naldi, 2019; Rinker, 2021), a package designed to calculate sentiment at the sentence-level, we calculated a sentiment score per sentence of dialogue. Importantly, “sentimentr” takes into account valence-shifting language (e.g., negators like “not” and amplifiers like “very”). The package uses a “bag of words” approach to compare sentences to a dictionary of emotional words and tag polarized words that appear in the sentence. Positive words are tagged with a + 1 value and negative words are tagged with a − 1 value. Then, a cluster of words (four before, two after) appearing around the polarized word is examined to identify valence shifters (neutral, negator, amplifier, de-amplifier).

Neutral words are not considered in the overall sentiment calculator, but polarized words are further weighted by the valence shifters surrounding them. Amplifiers increase the polarity by 1.8 and become de-amplifiers if the cluster contains an odd number of negators. Negators flip the sign of the polarized word. Furthermore, adversative conjunctions (such as “but,” “however”) increase the weight of the polarized word by 1.85 times the number of conjunctions *before* the polarized word and decrease the weight by 1.85 times the number of conjunctions *after* the polarized word. Together, these weights result in an unbounded sentiment score per sentence. Higher scores reflect a more positive sentiment while lower scores reflect more negative sentiment.

##### Clout 

Using the Linguistic Inquiry and Word Count (LIWC) software (Boyd et al., 2022), we calculated a clout score per sentence of dialogue. Clout refers to the relative social status, confidence, and self-assurance conveyed in dialogue. Clout scores are calculated using an algorithm that was developed based on research that identified language features that were relevant to individuals’ social positions or ranks (Kacewicz et al., 2014). Scores range from 1 to 99, with larger scores referring to higher clout.

#### Analytic approach

**Fig. 3:**
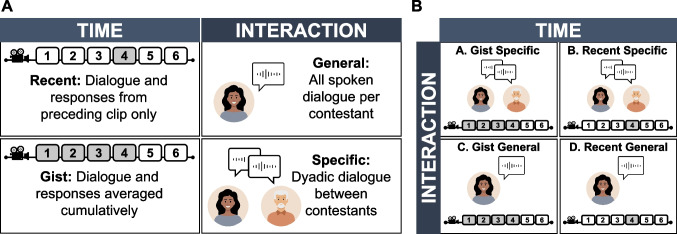
Overview of analytic approach, examining the relationship between social-relational inference and conversational linguistic features at four levels of analysis across time and interactions. (**A**) Overview of time and interaction levels. (**B**) Overview of combinatorial analytic representations using time and interaction levels. Numeric identifiers correspond to reported results (see Figs 5, 6, 7)

Due to the wealth of linguistic information across the *Survivor* episode, and the large number of relational judgments from participant responses, we examined the association between distinct linguistic features and social-relational inference at four levels of analysis: gist specific, recent specific, gist general, and recent general. These levels reflect unique combinations of time (recent, gist) and interaction (general, specific) to better understand how conversational content might inform social-relational inference on temporally and dyadically (Fig. 3A). We primarily focus on gist-specific representations to most clearly explore how verbal information from dyadic conversations inform social-relational judgments made by participant observers, but other levels are shown (Fig. 3B). Unless otherwise noted, other levels of analysis do not show evidence toward an interaction effect, and all discussed calculations were done independently for each linguistic feature (see Online Supplemental Material for a full summary of all results and an examination of all linguistic features together).

Levels of analysis were taken into account when calculating linguistic scores from conversational content. Each sentence-level similarity, sentiment, and clout score was averaged at the clip and interaction level, depending on the level of analysis. To calculate gist-specific and recent-specific scores, we calculated an average linguistic score per dyad, taking into account everything a unique pair of contestants said to each other in a given episode clip. Importantly this subset only includes dyadic dialogue spoken between two contestants. Gist-specific scores were calculated as a cumulative average for each clip and included all dialogue from the most recent and all preceding episode clips, while recent-specific scores only included dialogue from the most recent episode clip. For example, if calculating gist-specific clout for the Amber-Rob dyad from Clip 5, only dialogue spoken between Rob and Amber from Clips 1 through 5 would be included, but if calculating recent-specific clout, only dialogue spoken in Clip 5 would be included.

To calculate gist-general and recent-general scores, we calculated an average linguistic score per contestant, taking into account everything they said during a given episode clip. Importantly, this subset of dialogue includes everything a contestant said, and includes camera confessionals, interactions with groups, and interactions with the host. Gist-general scores were calculated as a cumulative average for each clip, while recent-general scores only included dialogue from the most recent episode clip. For example, if calculating gist-general sentiment for Amber in Clip 3, all of Amber’s spoken dialogue from across her interactions (e.g., with other contestants, the camera, the host) from Clips 1 through 3 would be included, but if calculating recent-general sentiment, only Amber’s dialogue from Clip 3 would be included.

**Fig. 4:**
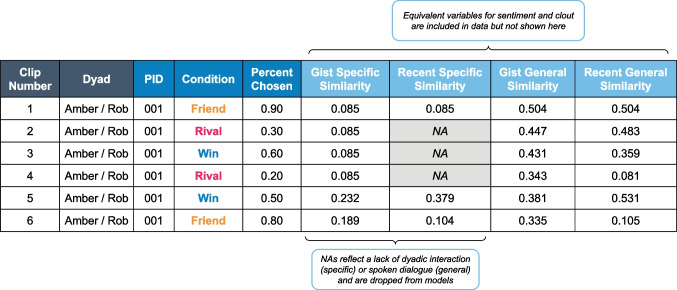
Example of data used to fit Bayesian models. Each row corresponds to a unique observation per participant, dyad, condition, and clip number. Missing values (*NA*) reflect the absence of a dyadic interaction (specific) or contestant dialogue (general). Dark blue variables correspond to overlapping data shared between experimental responses and language scores from the *Survivor* episode. Medium blue variables correspond to data from the experimental responses and light blue variables correspond to data from the *Survivor* episode conversations. Although not shown, there are four equivalent variables for both sentiment and clout and equivalent rows for all participants. Only complete cases are included in models at each level of analysis

This approach resulted in four distinct variable calculations for each of the three language features of interest (Fig. 4). These scores were subsequently merged with participant behavioral data and the final dataset was used to fit statistical models.

#### Bayesian modeling

Statistical analyses were performed using the “brms” package in R (Bürkner, 2018, 2021). Twelve models (four levels of analysis: recent general, recent specific, gist general, gist specific; three linguistic features: semantic similarity, sentiment, clout) were fit to investigate the association between each linguistic feature on relational judgments. The outcome variable (percent chosen) reflects a ratio of instances a contestant was selected to instances a contestant was presented to participants. All models included an interaction term between relational condition (friend, rival) and the model-specific linguistic feature (i.e., recent general semantic similarity). We also included a random effect of condition nested within contestant dyad and a random effect of condition nested within participant to appropriately account for repetitions in contestant presentations during the task and participant responses.

All Bayesian models used minimally informative priors, including a normal distribution with mean 0 and standard deviation 5 for fixed effects, a half-Cauchy distribution with mean 0 and standard deviation 5 for random effects, and a normal distribution with mean 0.5 and standard deviation 1 for the intercept. Each model specified 10,000 iterations (2,000 warm-up) of Markov chain Monte Carlo (MCMC) posterior sampling, four chains, and initial sampling values set to zero. Due to a low ratio of effective sample size to total sample size (*N*_*eff*_ < 0.1), we increased the iterations beyond 10,000 for the sentiment recent-specific model only (12,000 iterations, 3,000 warm-up). All other models had an acceptable ratio of effective sample size to total sample size (0% of samples *N*_*eff*_ < 0.1).

For all models, we used a beta-binomial response distribution with a logit link, modeling the outcome (% chosen) as a ratio of instances that contestants were presented as a choice to instances selected for a given target and condition. For example, Rob was presented as a friendship choice when Amber was the target contestant five times. If a given participant selected him three times, the dependent variable (percent chosen) is the ratio of 3/5 or 60%. 95% credibility intervals (CrIs) are presented in model results and 95%, 80%, and 50% credibility intervals are included in figures. Finally, we calculated a Bayes factor (BF) between the interaction and main effects (i.e., no interaction term between linguistic feature and condition) to determine evidence toward an interaction effect. Generally, BF values > 100 provide very strong evidence toward an effect, between 10 and 100 provide moderate evidence toward an effect, and < 10 provide little to no evidence toward an effect (Andraszewicz et al., 2015). See Online Supplemental Material for a summary of posterior predictive checks and MCMC chain convergence and resolution for all models (Kruschke, 2021).

### Results

#### Gist-specific semantic similarity was positively associated with perceived friendship and negatively associated with perceived rivalry

**Fig. 5:**
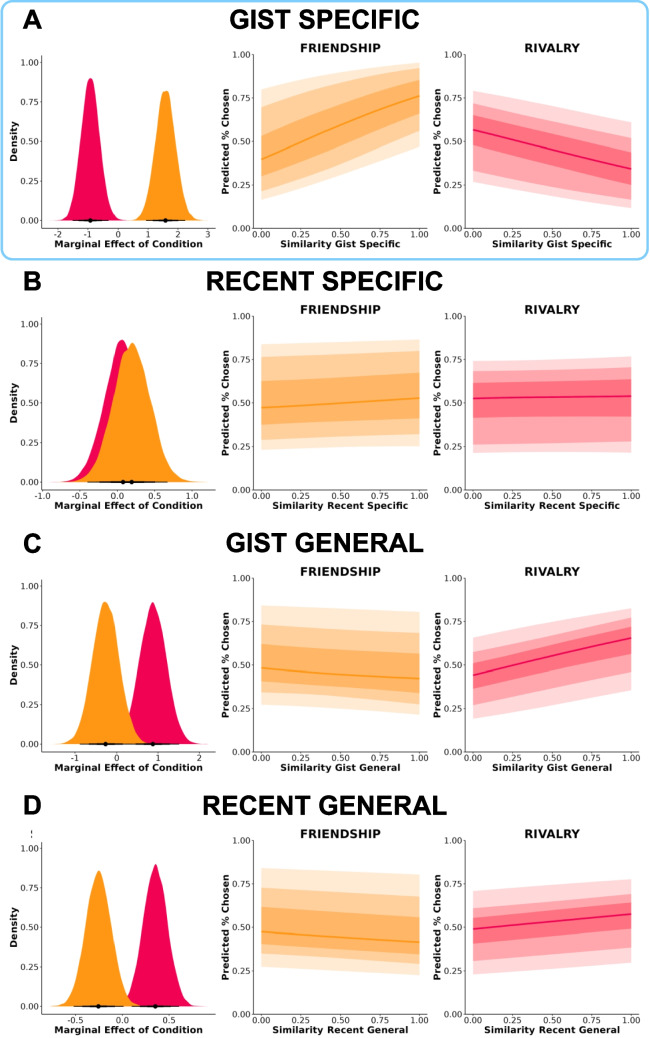
Associations between semantic similarity and social-relational inference. (**A**) Gist specific. (**B**) Recent specific. (**C**) Gist general. (**D**) Recent general. Dark to light shading gradients reflect 50%, 80%, and 95% credibility intervals, respectively. Marginal effects and expected outcomes (predicted % chosen) are plotted using 1,000 randomly selected posterior draws. Blue border denotes strong evidence toward a relational interaction effect

First, we examined to what extent gist-specific semantic similarity was associated with social-relational inference (Fig. 5A). Gist-specific similarity was differentially associated with friendship and rivalry judgments, *b* = −2.500, *95% CrI* [−3.502, −1.495], *BF* = 12,595.93. The marginal effect for gist specific similarity was *b* = 1.582, *95% CrI* [0.932, 2.243] for friendship and *b* = −0.919, *95% CrI* [−1.524, −0.326] for rivalry.

There was no association between recent-specific semantic similarity and perceived friendship and rivalry (Fig. 5B). However, gist-general (Fig. 5C) and recent-general (Fig. 5D) semantic similarity were both weakly negatively associated with perceived friendship and positively associated with perceived rivalry. Gist-general similarity was weakly differentially associated with friendship and rivalry judgments, *b* = 1.134, *95% CrI* [0.194, 2.066], *BF* = 1.627. The marginal effect for gist-general similarity was *b* = −0.261, *95% CrI* [−0.869, 0.381] for friendship and *b* = 0.874, *95% CrI* [0.236, 1.516] for rivalry. Recent-general similarity was weakly differentially associated with friendship and rivalry judgments, *b* = 0.606, *95% CrI* [0.239, 0.975], *BF* = 6.321. The marginal effect for recent-general similarity was *b* = −0.254, *95% CrI* [−0.516, 0.014] for friendship and *b* = 0.353, *95% CrI* [0.089, 0.596] for rivalry.

#### Gist-specific sentiment was positively associated with perceived friendship and negatively associated with perceived rivalry

**Fig. 6:**
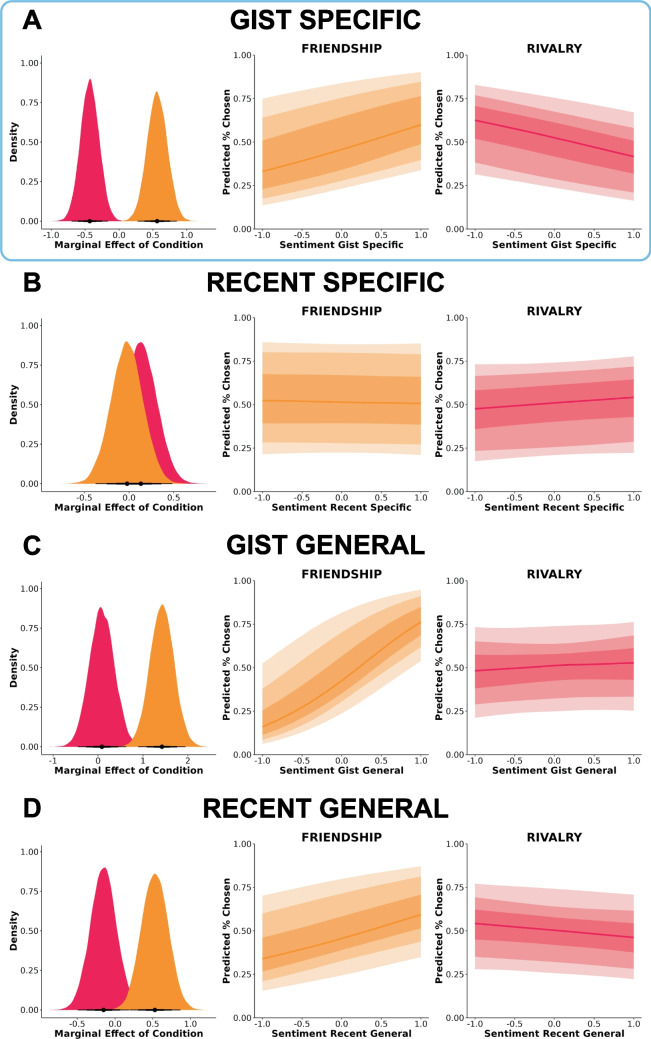
Associations between sentiment and social-relational inference. (**A**) Gist specific. (**B**) Recent specific. (**C**) Gist general. (**D**) Recent general. Dark to light shading gradients reflect 50%, 80%, and 95% credibility intervals, respectively. Marginal effects and expected outcomes (predicted % chosen) are plotted using 1,000 randomly selected posterior draws. Blue border denotes strong evidence toward a relational interaction effect

We first examined to what extent gist-specific sentiment was associated with social-relational inference (Fig. 6A). Gist-specific sentiment was differentially associated with friendship and rivalry judgments, *b* = −0.996, *95% CrI* [−1.425, −0.570], *BF* = 1475.313. The marginal effect for gist-specific sentiment was *b* = 0.563, *95% CrI* [0.277, 0.857] for friendship and *b* = −0.432, *95% CrI* [−0.696, −0.160] for rivalry.

There was no association between recent-specific sentiment and perceived friendship and rivalry (Fig. 6A). However, gist-general sentiment (Fig. 6C) and recent-general sentiment (Fig. 6D) were weakly positively associated with perceived friendship and negatively associated with perceived rivalry. Gist-general sentiment was differentially associated with friendship and rivalry judgments, *b* = −1.335, *95% CrI* [−2.086, −0.567], *BF* = 29.246. The marginal effect for gist general sentiment was *b* = 1.423, *95% CrI* [0.897, 1.949] for friendship and *b* = 0.084, *95% CrI* [−0.446, 0.628] for rivalry. Recent-general sentiment was weakly differentially associated with friendship and rivalry judgments, *b* = −0.681, *95% CrI* [−1.163, −0.202], *BF* = 2.470. The marginal effect for recent-general sentiment was *b* = 0.527, *95% CrI* [0.191, 0.865] for friendship and *b* = −0.153, *95% CrI* [−0.483, 0.194] for rivalry.

#### Gist-specific and recent-specific clout were positively associated with perceived friendship and negatively associated with perceived rivalry

**Fig. 7:**
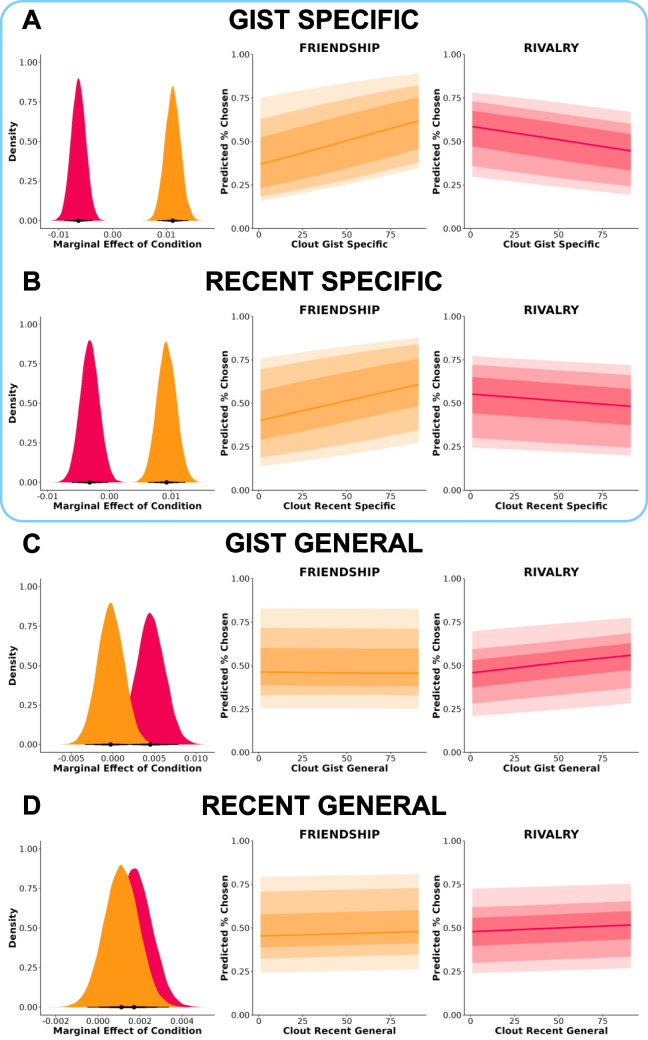
Associations between clout and social-relational inference. (**A**) Gist specific. (**B**) Recent specific. (**C**) Gist general. (**D**) Recent general. Dark to light shading gradients reflect 50%, 80%, and 95% credibility intervals, respectively. Marginal effects and expected outcomes (predicted % chosen) are plotted using 1,000 randomly selected posterior draws. Blue border denotes strong evidence toward a relational interaction effect

We first examined to what extent gist-specific clout was associated with social-relational inference (Fig. 7A). Gist-specific clout was differentially associated with friendship and rivalry judgments, *b* = −0.018, *95% CrI* [−0.022, −0.014], and there is especially strong evidence toward this effect, *BF* = 5,129,119,269,855.19. The marginal effect for gist specific clout was *b* = 0.011, *95% CrI* [0.008, 0.014] for friendship and *b* = −0.006, *95% CrI* [−0.009, −0.004] for rivalry.

Next, we examined to what extent recent-specific clout was associated with social-relational inference (Fig. 7B). Recent-specific clout was differentially associated with friendship and rivalry judgments, *b* = −0.013, *95% CrI* [−0.017, −0.008], *BF* = 3899.30. The marginal effect for recent-specific clout was *b* = 0.009, *95% CrI* [0.006, 0.012] for friendship and *b* = −0.003, *95% CrI* [−0.006, −0.0003] for rivalry.

Gist-general clout was weakly positively associated with perceived friendship and negatively associated with perceived rivalry (Fig. 7C). Gist-general clout was somewhat differentially associated with friendship and rivalry judgments, *b* = 0.005, *95% CrI* [0.0002, 0.009], but there is weak evidence toward this effect, *BF* = 0.004. The marginal effect for gist-general clout was *b* = −0.0003, *95% CrI* [−0.003, 0.003] for friendship and *b* = 0.005, *95% CrI* [0.001, 0.008] for rivalry. There was no association between recent-general clout and perceived friendship and rivalry (Fig. 7D).

### Discussion

These results suggest that there is a consistent and strong association between gist-specific linguistic information (i.e., specific dyadic interactions represented cumulatively across the episode) and relational judgments, such that greater semantic similarity, more positive sentiment, and higher clout were positively associated with friendship judgments. Conversely, less semantic similarity, more negative sentiment, and lower clout were associated with rivalry judgments. The association between conversational linguistic features and social-relational inference varies widely across features and levels of analysis, and we summarized and compared these models in the Online Supplemental Material to examine to what extent all three language features together are associated with social-relational inference. However, since relationships unfold over time, we wanted to further examine if early semantic similarity is associated with later relational judgments and explore if similarity precedes relational homophily (Parkinson et al., 2018).

## Language similarity and relational homophily

### Method

#### Episode transcriptions

**Fig. 8:**
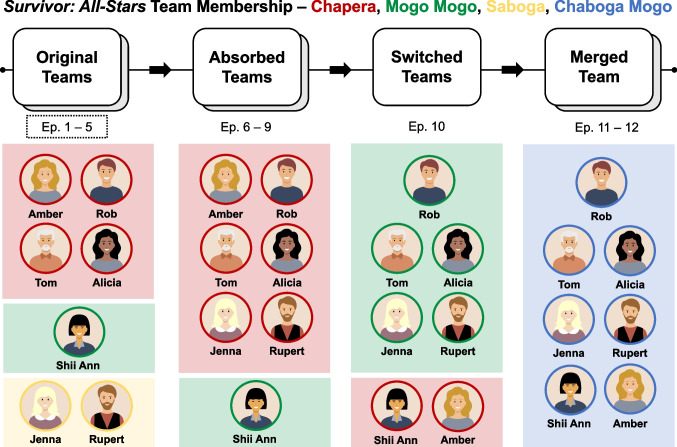
*Survivor: All-Stars* team membership*.* All episodes in the season leading up to the episode presented to participants are shown. Only contestants remaining in the presented episode are shown, but teams included additional members that were voted off over the course of the season. During the first five episodes, contestants are split into three teams – Chapera, Mogo Mogo, and Saboga. Only transcribed conversational dialogue from these first five episodes are included in analyses (dotted outline). In Episode 6, teams are merged, and the Saboga team is absorbed into the remaining two teams, Chapera and Mogo Mogo. These two teams remain for Episodes 6 through 9, but in Episode 10, all contestants are randomly swapped. All but one Chapera member (Amber) moved over to Mogo Mogo. Finally, in Episode 11, Chapera and Mogo Mogo merge into one team called Chaboga Mogo. The episode presented to participants (Episode 12) includes this remaining merged team. Team colors reflect the colors shown on *Survivor* – Chapera team members are denoted with red outlines and shading, Mogo Mogo is green, Saboga is yellow, and Chaboga Mogo is blue. The recap episode is not included and episode numbers in the figure reflect recoded numbers skipping the recap

Two coders transcribed dialogue from all *Survivor* episodes that preceded the episode presented to participants (Season 8, Episodes 1– 8, Episodes 10–12; CBS Television). We did not transcribe Episode 9 as it was a recap episode with no additional narrative content. We recoded episode numbers to reflect the continuity of televised content, such that Episodes 10 through 13 became 9 through 12. As in the previous episode transcription, the whole-season transcriptions noted all spoken dialogue, the speaker’s name, the recipient’s name, and episode number. The transcriptions were further organized at the sentence level, such that each sentence had a corresponding speaker and recipient. To examine if early semantic similarity was associated with later relational judgments, we only included the first five episodes (*N*_*episodes*_ = 5) in these analyses. We focus on the initial five episodes as they capture the original team structure of the season (i.e., before contestants switch or merge teams; Fig. 8), and thus present a more conservative context in which to test our hypothesis that early linguistic similarity predicts later relational formation (e.g., relational homophily).^2^

#### Semantic similarity

Semantic similarity was calculated using the same approach outlined above. We examined the association between conversational similarity in early episodes and later social-relational inference at two levels of analysis: *general* and *specific*. These levels reflect the conversational content spoken by the contestants who appear in our focal episode across the first five episodes in the season, with general scores taking into account everything a contestant said (including camera confessionals, interactions with groups, and interactions with host) and specific scores only taking into account dyadic interactions between focal episode contestants. As an example, Amber’s dialogue to all parties and Rob’s dialogue to all parties would be used to calculate their *general* similarity scores, while dyadic interactions exclusively between Rob and Amber would be used to calculate their *specific* similarity scores.

#### Bayesian modeling

Statistical analyses were performed using the “brms” package in R (Bürkner, 2018, 2021). Two models were fit to investigate the association between early semantic similarity and later relational judgments. The outcome variable (percent chosen) reflects a ratio of instances a contestant was selected to instances a contestant was presented to participants. These models included an interaction term between relational condition (friend, rival) and semantic similarity (general, specific) averaged across the first five episodes. We also included a random effect of condition nested within contestant dyad and a random effect of condition nested within participant to appropriately account for repetitions in contestant presentations during the task and participant responses. All Bayesian models used the same priors and specifications as outlined in the previous section.

### Results

**Fig. 9:**
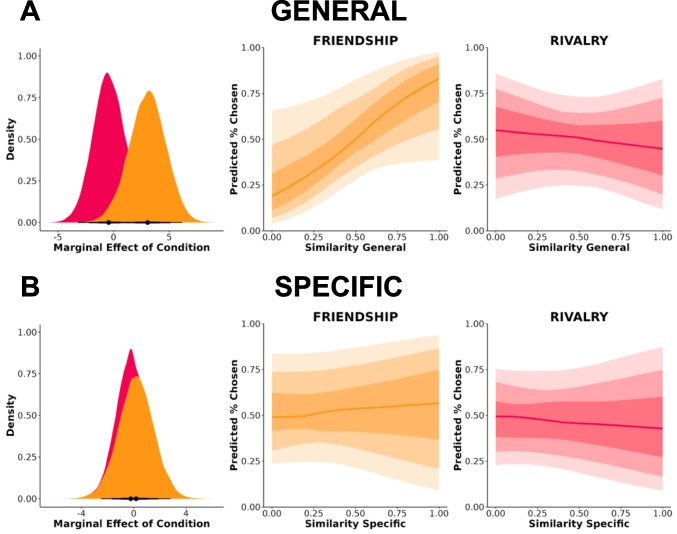
No associations between early semantic similarity and later social-relational inference. (**A**) General. (**B**) Specific. Dark to light shading gradients reflect 50%, 80%, and 95% credibility intervals, respectively. Marginal effects and expected outcomes (predicted % chosen) are plotted using 1,000 randomly selected posterior draws

There was no association between general (Fig. 9A) or specific (Fig. 9B) semantic similarity in early episodes and later perceived friendship and rivalry. Early general semantic similarity was not differentially associated with later friendship and rivalry judgments, *b* = −3.422, *95% CrI* [−8.975, 2.437], *BF* = 1.210. The marginal effect for general semantic similarity was *b* = 3.063, *95% CrI* [−0.291, 6.16] for friendship and *b* = −0.432, *95% CrI* [−3.269, 2.44] for rivalry. Early specific semantic similarity was also not differentially associated with later friendship and rivalry judgments, *b* = −0.403, *95% CrI* [−5.151, 4.438], *BF* = 0.452. The marginal effect for specific semantic similarity was *b* = 0.180, *95% CrI* [−2.42, 2.87] for friendship and *b* = −0.231, *95% CrI* [−2.47, 2.09] for rivalry.

### Discussion

Individuals must synthesize complex, multi-stream information to learn about novel social relationships. Our results suggest that information from one stream – conversations – may assist people in learning about the nature and strength of these relationships. Moreover, by leveraging a novel stimulus (*Survivor*) and examining naturalistic social-relational inference in a brief laboratory session, this work is a potentially meaningful advancement in the fields of cognitive and social psychology.

Because of the naturalistic nature of our stimulus, it is difficult to determine an underlying “truth” to the contestant relationships shown in the episode. However, our ISC approach allowed us to closely examine shared pairwise representational similarities of contestant relationships and connect them to tangible season outcomes. Participant responses were highly similar to each other across conditions (friend, rival, win), suggesting that participants gathered information that guided them to similar judgments about the nature of contestant relationships. Notably, participants had not seen this season of *Survivor*, but friendship judgments were more consistent with the results of the show than win assessments. That is, the average friendship score for each individual contestant (e.g., the average percent of time a contestant was chosen as a friend by participants) exactly followed the subsequent order of contestants voted off the show in this and subsequent episodes (discounting immunity). Moreover, the two contestants who were most often chosen as friends by participants (Rob and Amber), got married at the end of the season, suggesting that the information that participants learned about dyads via passive observation tracked real-world relationship qualities.

Turning more generally to learning, our linguistic findings support prior work underscoring language’s informative value for individual and social judgments (Tong et al., 2020). Consistent with prior research (Babcock et al., 2014; Cannava & Bodie, 2017; Ireland et al., 2011), we found evidence across analytic approaches that greater semantic similarity, more positive sentiment, and higher clout were associated with friendship judgments, and less semantic similarity, more negative sentiment, and lower clout were associated with rivalry judgments. This pattern of linguistic results indicates that information gathered via specific dyadic exchanges over time (i.e., gist specific) is most predictive of social-relational inference. This cumulative information accrual of multiple conversational features is associated with the degree and valence of social relationships perceived by participants. This was largely not the case for language used by the same contestants in interactions that were not dyad-specific, nor for dyad-specific interactions that occurred during the most recent snapshot of time. This suggests that social-relational inference is likely the result of a multi-component process that spans across an observed time period, considering past observations and dyadic-specific interactions rather than reflecting general language use at the individual level or anchoring to the most recent exchanges between individuals. Future research should examine the extent to which these and other linguistic features, and cumulative versus recent timescales of conversational interactions, map on to other types of relationships or social environments (e.g., communal vs. exchange, competitive vs. cooperative) (Clark & Mills, 1993).

We also examined early levels of semantic similarity at two levels of analysis and related those to relational inferences made during later evaluation. Counter to our predictions, and inconsistent with prior work (Bahns et al., 2017; Parkinson et al., 2018), we did not find evidence that early season linguistic similarity predicts later season friendship judgments. This was the case for both an individual’s general interactions with others and their specific interactions with later teammates. These longitudinal results for specific interactions between dyads initially seem counter to our linguistic results in the focal episode, which could be due to a number of reasons.

First, it is possible that this analysis lacks statistical power. Given the design of *Survivor*, there are many more individuals in early episodes (Episode 1: 18 starting contestants; Episode 5: 14 remaining contestants), meaning that individuals from our focal episode are likely having less frequent and comparatively shorter interactions as compared to later in the season. It is also possible that *Survivor* is a context that is fundamentally distinct from previously studied social environments – wherein early homophily predicts relational formation – that are largely focused on positive affiliation relationships like friendships or romantic partnerships. For example, individuals who are more similar to others at an early stage of a competitive game like *Survivor* could be viewed as particularly threatening rivals, reducing the value of the distinct contributions that they could make to their team (Berendt et al., 2018). In our focal episode, the positive association between semantic similarity and friendship may also be indicative of the adoption of shared goals (or alliances, in the parlance of reality television), that emerge over time. This adoption of shared goals, alliances, and constrained environmental context may also explain the weak positive association between gist- and recent-general semantic similarity and perceived rivalry in the focal episode. Future research should examine how the overarching social goals of an environment may inform how previously examined interaction features like homophily predict initial relational formation and the strength of those relationships over time. These results are particularly exciting and are among the first to address whether homophily is a cause or consequence of friendship in a competitive environment.

Although *Survivor* is an excellent naturalistic stimulus to use to explore our research questions, it is not without limitations. The competitive nature of the content may influence participant behavior such that win judgments were easier to make than friendship or rivalry judgments. Participants gather more salient win information after seeing how contestants perform in challenges or upon learning who is voted out by their peers, but they may have more ambiguity about friendships and rivalries depending on observed interactions and voting decisions. Our use of *Survivor* as a stimulus contributes to advancements in naturalistic experimentation (e.g., using movies and television; Chen et al., 2017; Jolly et al., 2023), and a key strength of reality television in particular is that it contains unscripted conversations (Grall & Finn, 2022). However, this needed improvement is also imperfect, since reality television content is highly curated and produced, and the editing may implicitly control the narrative of friendship and rivalries depicted. Nonetheless, we can still closely simulate the social-relational inference experience that people encounter in everyday life, wherein affiliative, adversarial, and competitive aspects may be similarly intertwined (rather than isolated) in relationships. Moreover, television is made with the viewer in mind, which complements our perceiver-directed natural language-processing methods.

Taken together, this work combines a novel, naturalistic stimulus – *Survivor* – with innovative and comprehensive language analysis methods to understand how conversations inform people of the underlying psychology and nature of social relationships.

## Supplementary Information

Below is the link to the electronic supplementary material.

Supplementary file1 (PDF 11817 KB)

## Data Availability

De-identified data and code are publicly accessible at https://osf.io/8wqhb/.

## Footnotes

1 We conducted analyses including and excluding two participants who missed responses to 50 or more trials and saw a similar pattern of results. We ultimately included these participants in the final sample.

2 Given that our primary research question for this analysis relates to linguistic similarity and relational homophily, we did not analyze sentiment or clout in this context.

## Code availability

Code is publicly available at https://osf.io/8wqhb/.

## Declarations

### Open practices statement

Data and materials are available at https://osf.io/8wqhb/.
