# Supplementary material for: Conversational linguistic features inform social-relational inference
Source: Psychon Bull Rev. 2025 Mar 6;32(4):1860–77. doi: 10.3758/s13423-025-02654-0 (PMC12325574; doi:10.3758/s13423-025-02654-0)
Supplement: Supplementary file 1 — Supplementary file1 (PDF 11817 KB) [file 13423_2025_2654_MOESM1_ESM.pdf]

Supplemental

Social-Relational Inference Similarity......2
Average Contestant Presentations and Selections...... 2
Average Contestant Presentations and Selections by Condition...... 4
Average Participant Responses Overall and by Condition...... 6
Proportion of Trials Selected per Condition...... 8
Bootstrapping Distributions...... 11
Conversational Linguistic Features......14
Sentiment & Clout Conversation Examples......14
Semantic Similarity...... 15
Gist Specific...... 15
Recent Specific...... 18
Gist General......21
Recent General......24
Sentiment......27
Gist Specific......27
Recent Specific......30
Gist General......33
Recent General......36
Clout...... 39
Gist Specific......39
Recent Specific......42
Gist General......45
Recent General......48
LASSO Regression – Combined Linguistic Features...... 51
Language Similarity and Relational Homophily...... 53
General...... 53
Specific...... 56
Supplemental References......59

Social-Relational Inference Similarity

Average Contestant Presentations and Selections

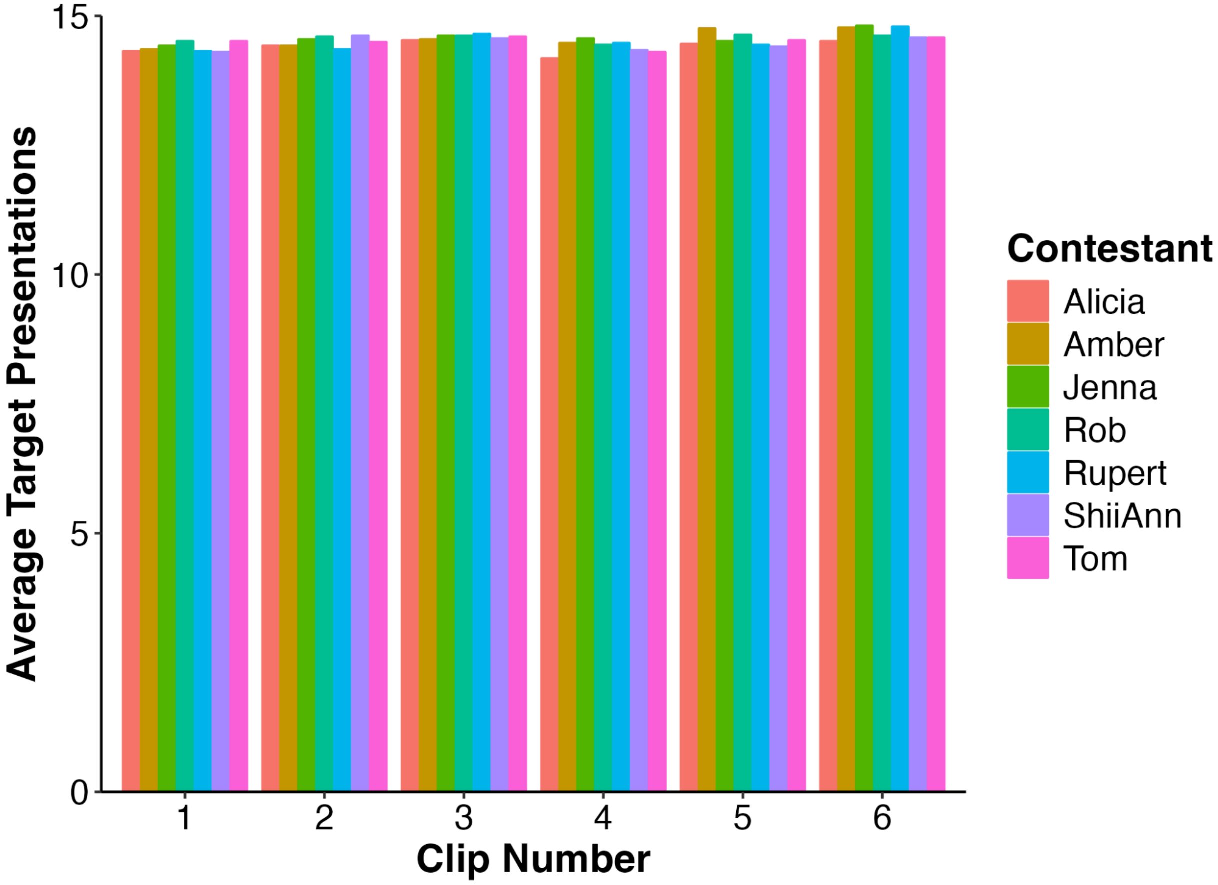

Supp. Fig. 1. Average number of target presentations per Survivor contestant across episode clips. Clip numbers (1 – 6) displayed on x-axis and average number of target presentations in included data displayed on the y-axis. The target participant was displayed on the top of the screen, above the relational (friend, rival) or non-relational (win) assessment of interest and choice pair. Bar color denotes contestant identity.

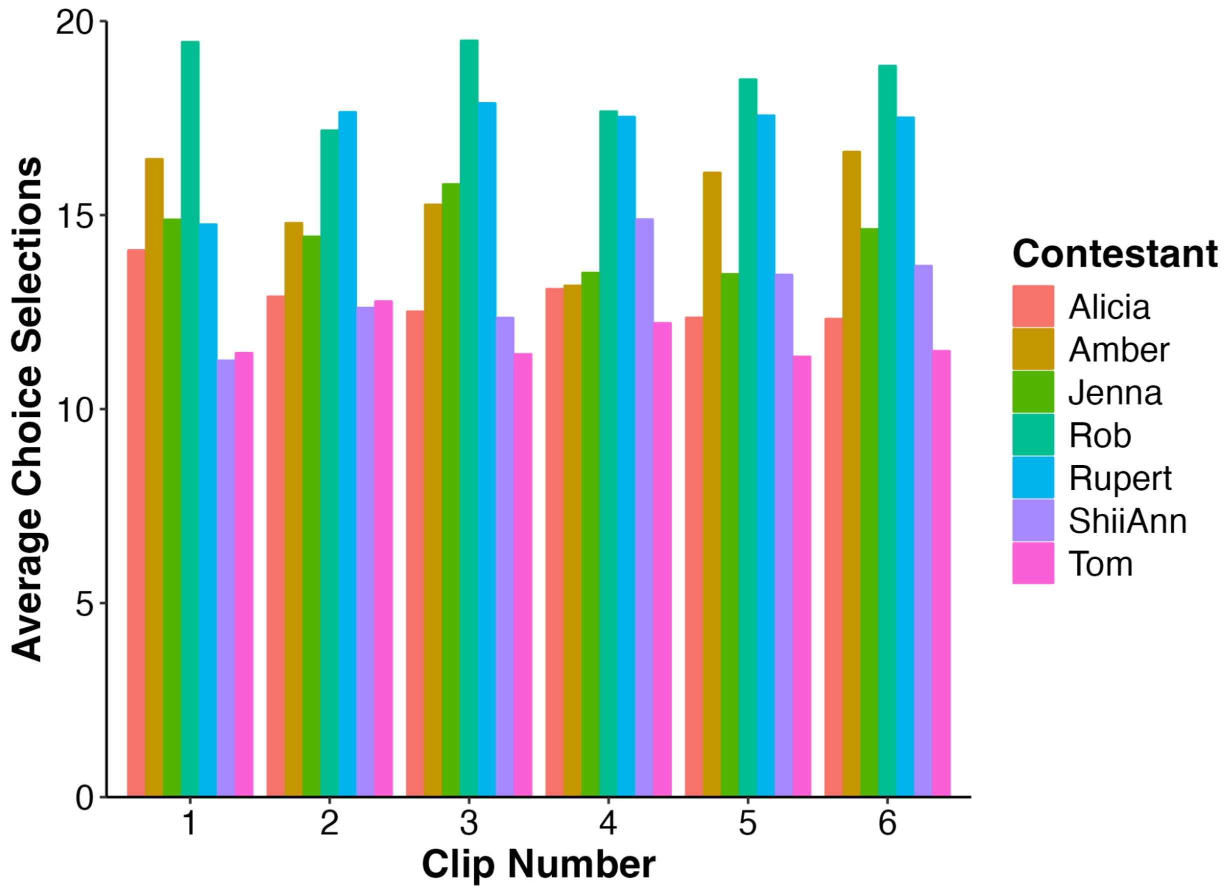

Supp. Fig. 2. Average number of choice selections per *Survivor* contestant across episode clips. Clip numbers (1 – 6) displayed on x-axis and average number of choice selections in included data displayed on the y-axis. Choice selections refer to how often a participant was chosen in response to the relational or non-relational assessment of interest. Bar color denotes contestant identity.

Average Contestant Presentations and Selections by Condition

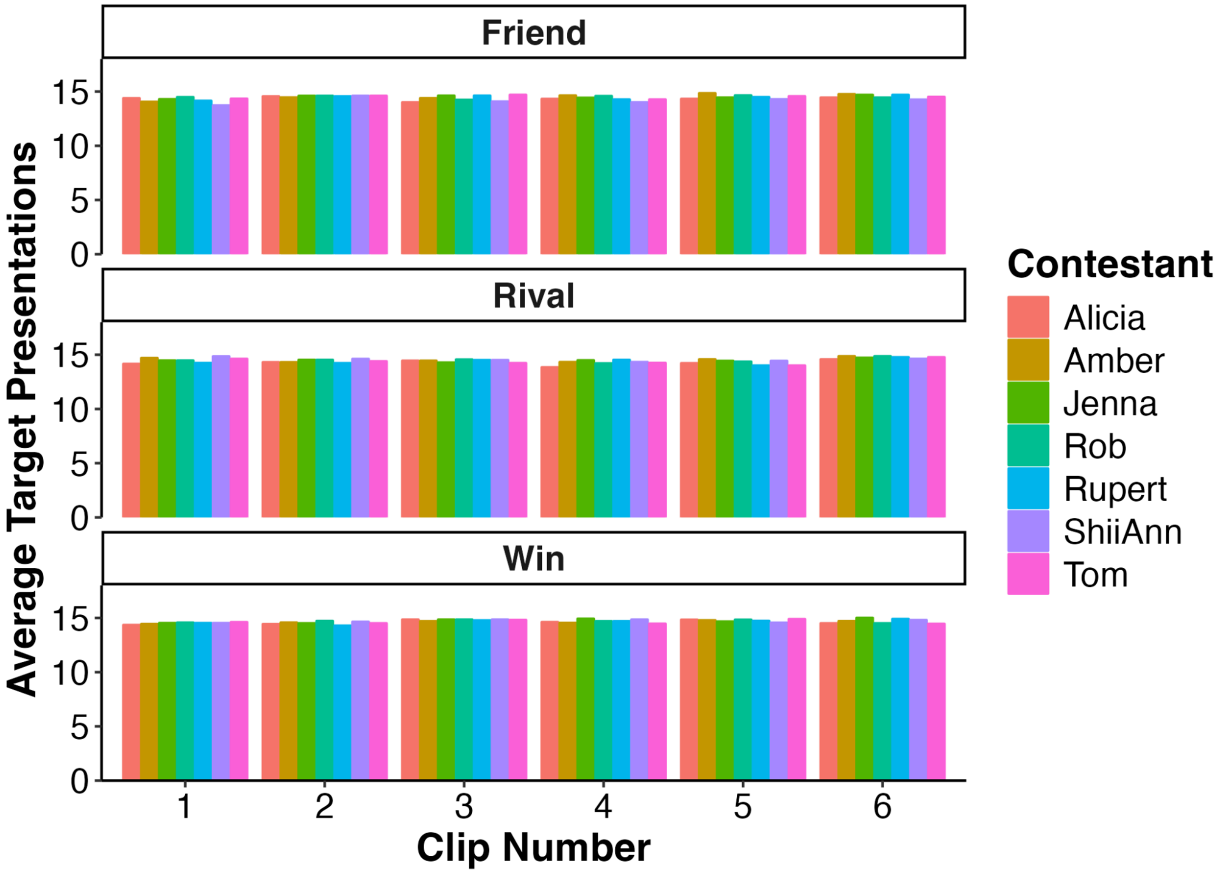

Supp. Fig. 3. Average number of target presentations per *Survivor* contestant across episode clips and experimental conditions. Clip numbers (1 – 6) displayed on x-axis and average number of target presentations in included data displayed on the y-axis. Bar color denotes contestant identity. Figure facets denote responses for friend, rival, and win conditions.

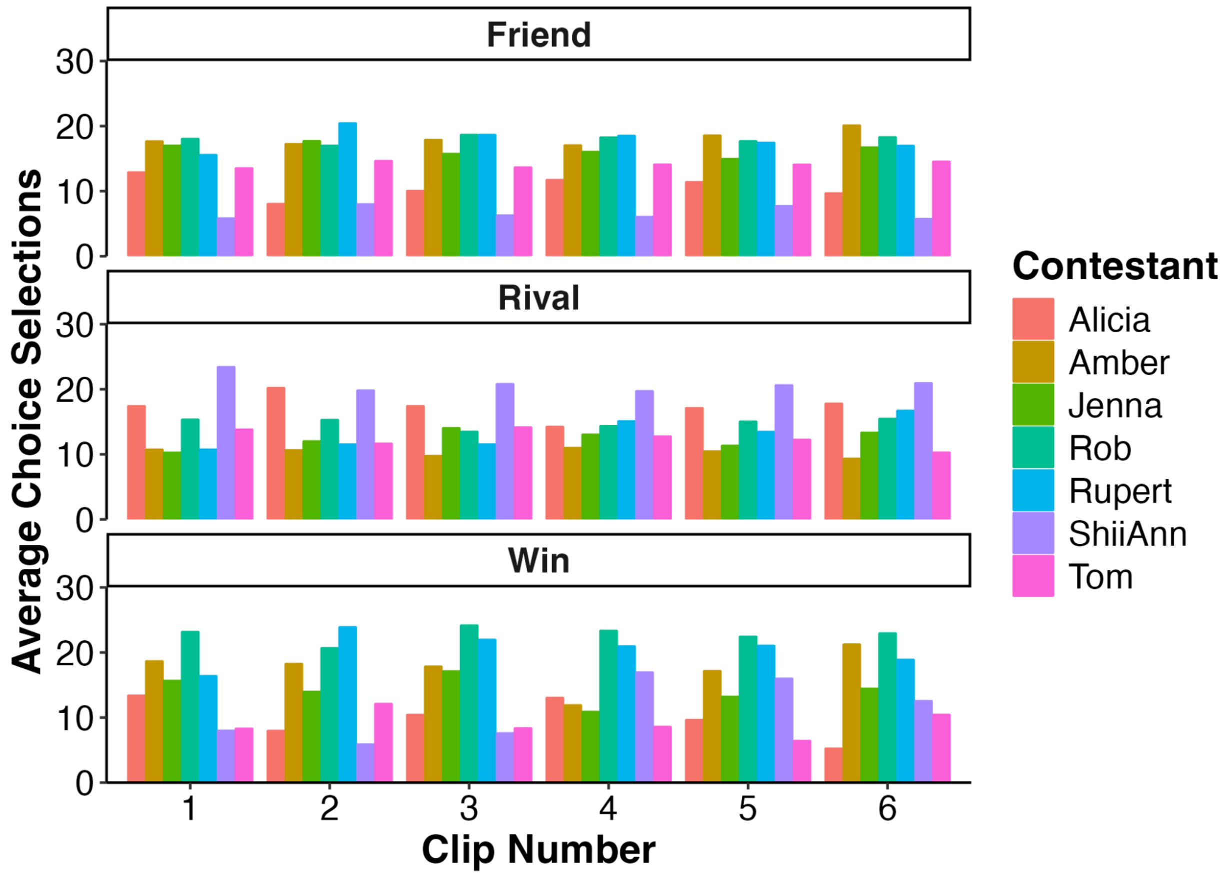

Supp. Fig. 4. Average number of choice selections per *Survivor* contestant across episode clips and experimental conditions. Clip numbers (1 – 6) displayed on x-axis and average number of choice selections in included data displayed on the y-axis. Bar color denotes contestant identity. Figure facets denote responses for friend, rival, and win conditions.

Average Participant Responses Overall and by Condition

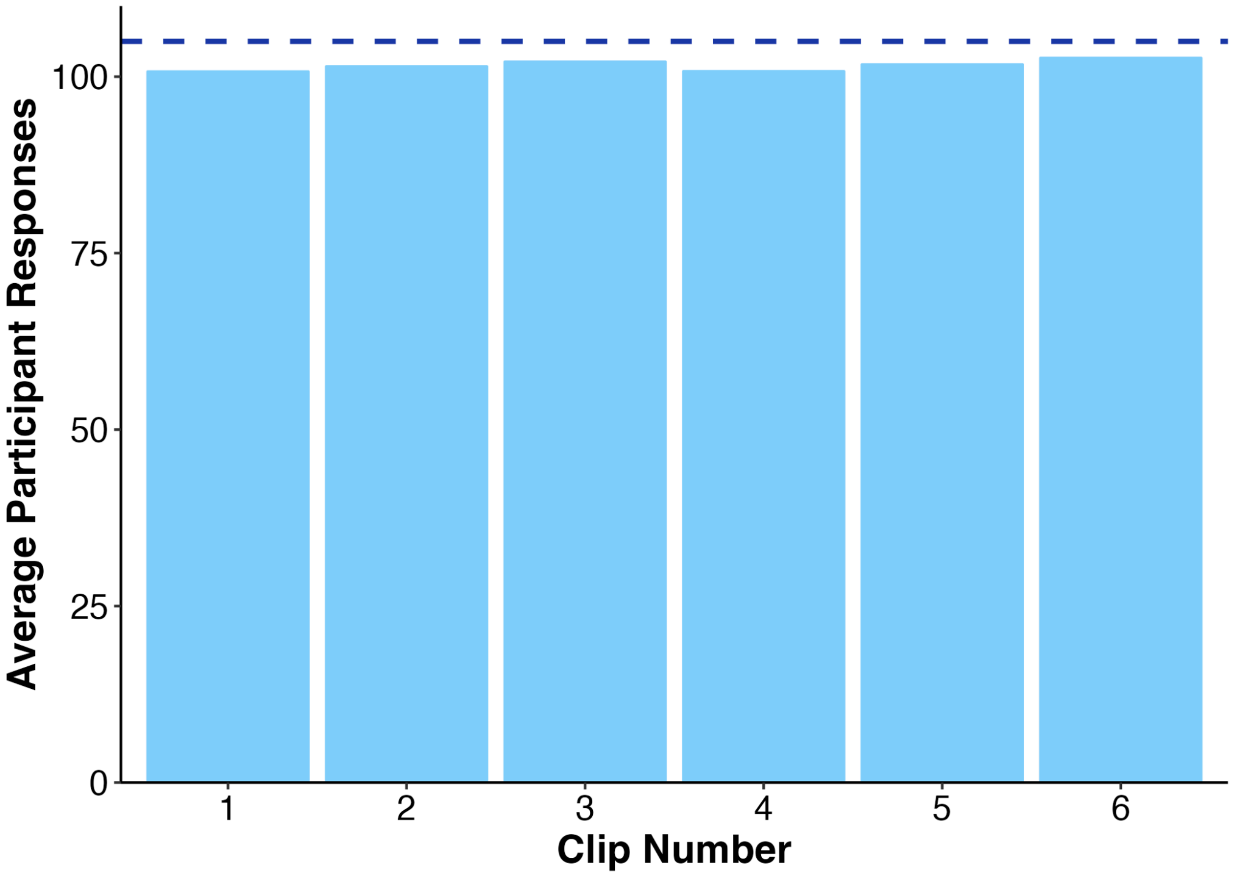

Supp. Fig. 5. Average number of participant responses made per episode clip. Clip numbers (1 – 6) displayed on x-axis and average number of participant responses in included data displayed on the y-axis. Horizontal dotted line denotes the maximum possible number of responses per experimental block (105).

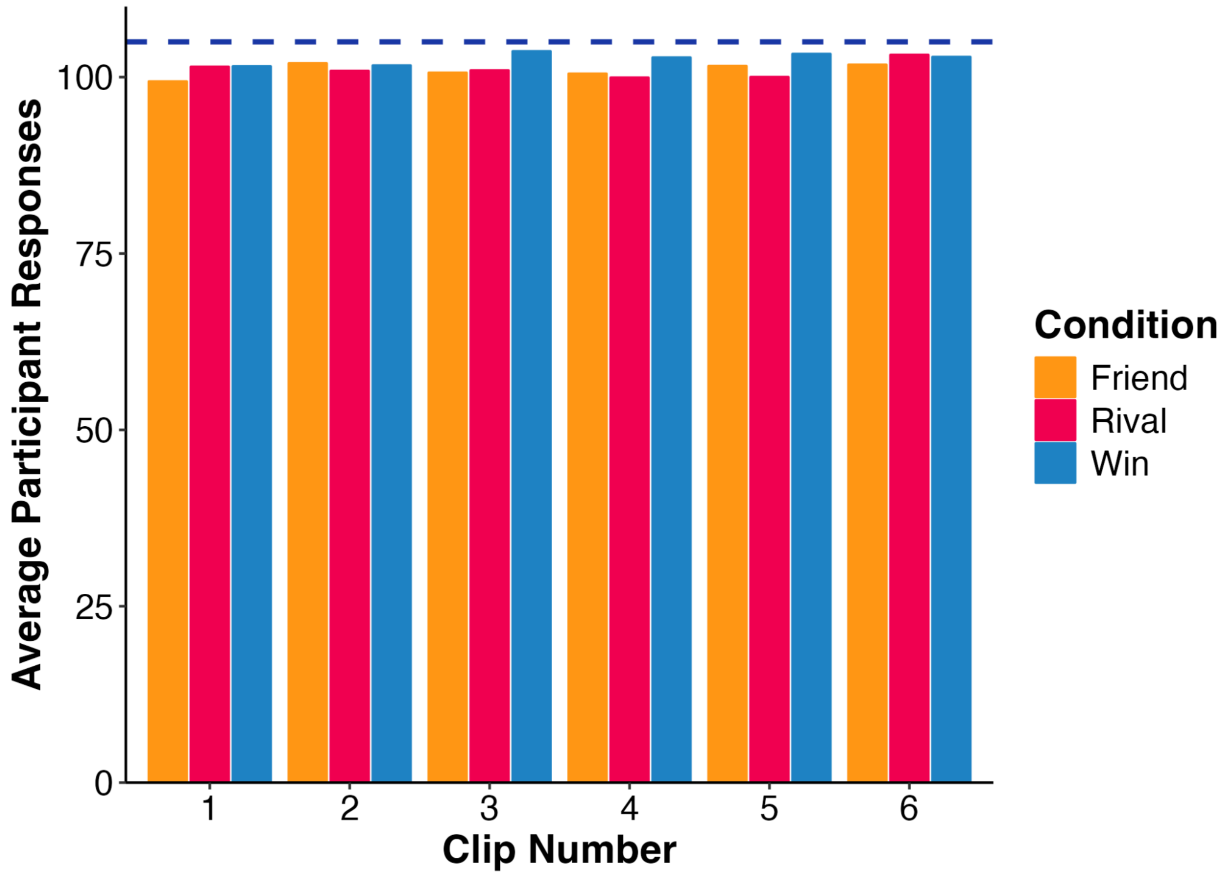

Supp. Fig. 6. Average number of participant responses made per episode clip by experimental condition. Clip numbers (1 – 6) displayed on x-axis and average number of participant responses in included data displayed on the y-axis. Horizontal dotted line denotes the maximum possible number of responses per experimental block (105). Bar colors denote experimental response conditions (friend, rival, win).

Proportion of Trials Selected per Condition

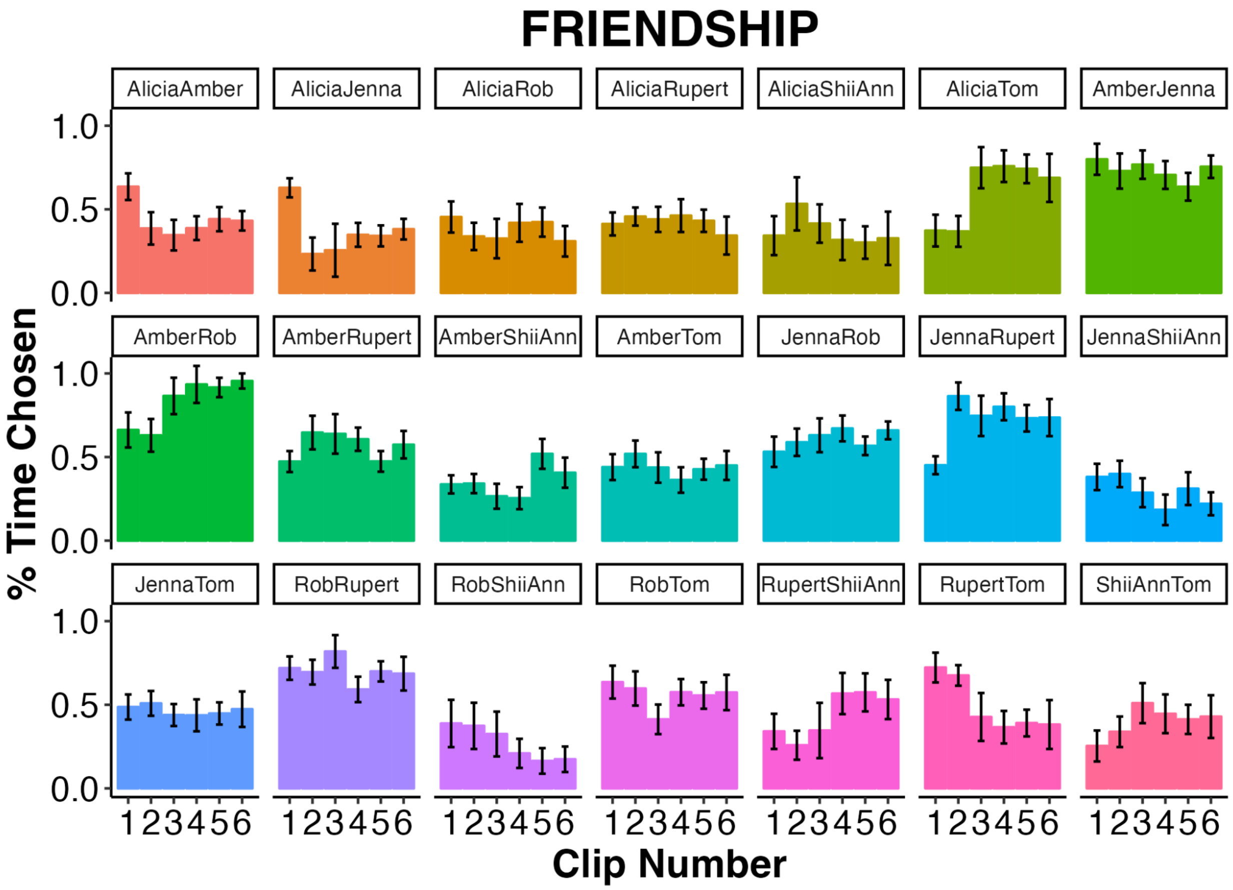

Supp. Fig. 7. Dyadic friendship responses. Clip numbers (1 – 6) displayed on x-axis. Average % time each dyad was chosen as friends across participants displayed on y-axis. Error bars reflect 95% confidence intervals.

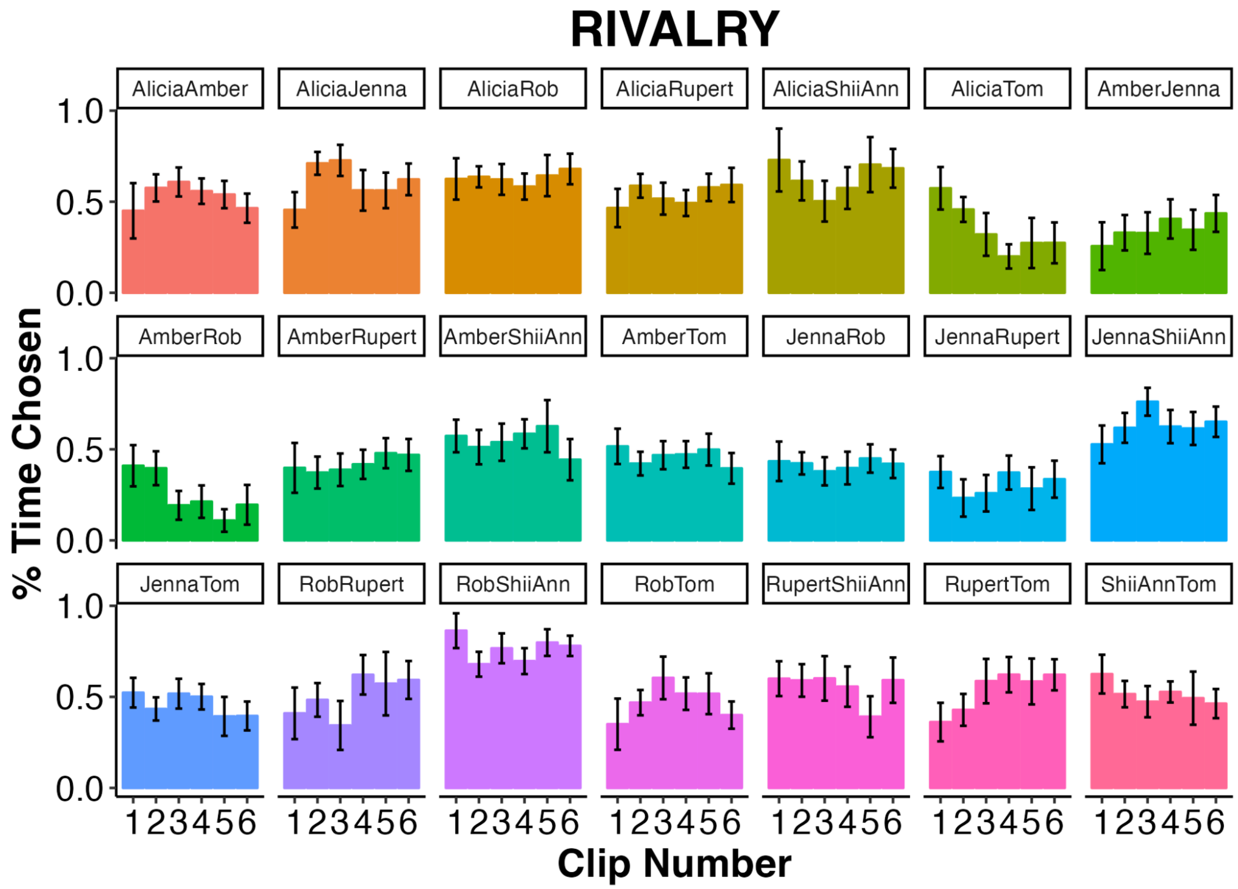

Supp. Fig. 8. Dyadic rivalry responses. Clip numbers (1 – 6) displayed on x-axis. Average % time each dyad was chosen as rivals across participants displayed on y-axis. Error bars reflect 95% confidence intervals.

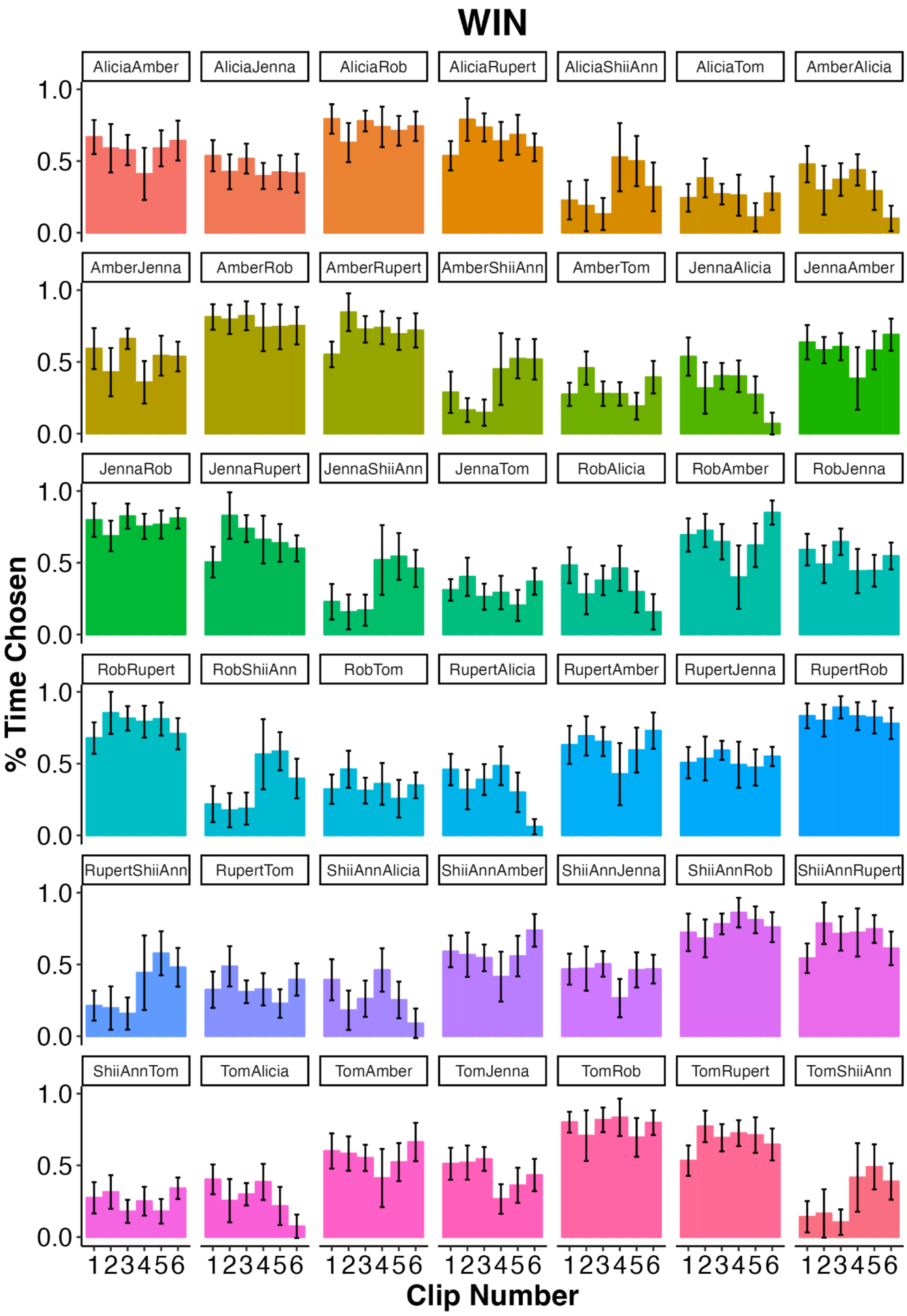

Supp. Fig. 9. Dyadic win responses. Clip numbers (1 – 6) displayed on x-axis. Average % time chosen one individual was chosen as a winner over the other across participants displayed on y-axis. Each dyad is shown twice since a win average reflects the % time a contestant was chosen as a predicted winner *over* the target contestant. The second name in the dyad name reflects the contestant chosen to win over the first name in the dyad. Error bars reflect 95% confidence intervals.

Bootstrapping Distributions

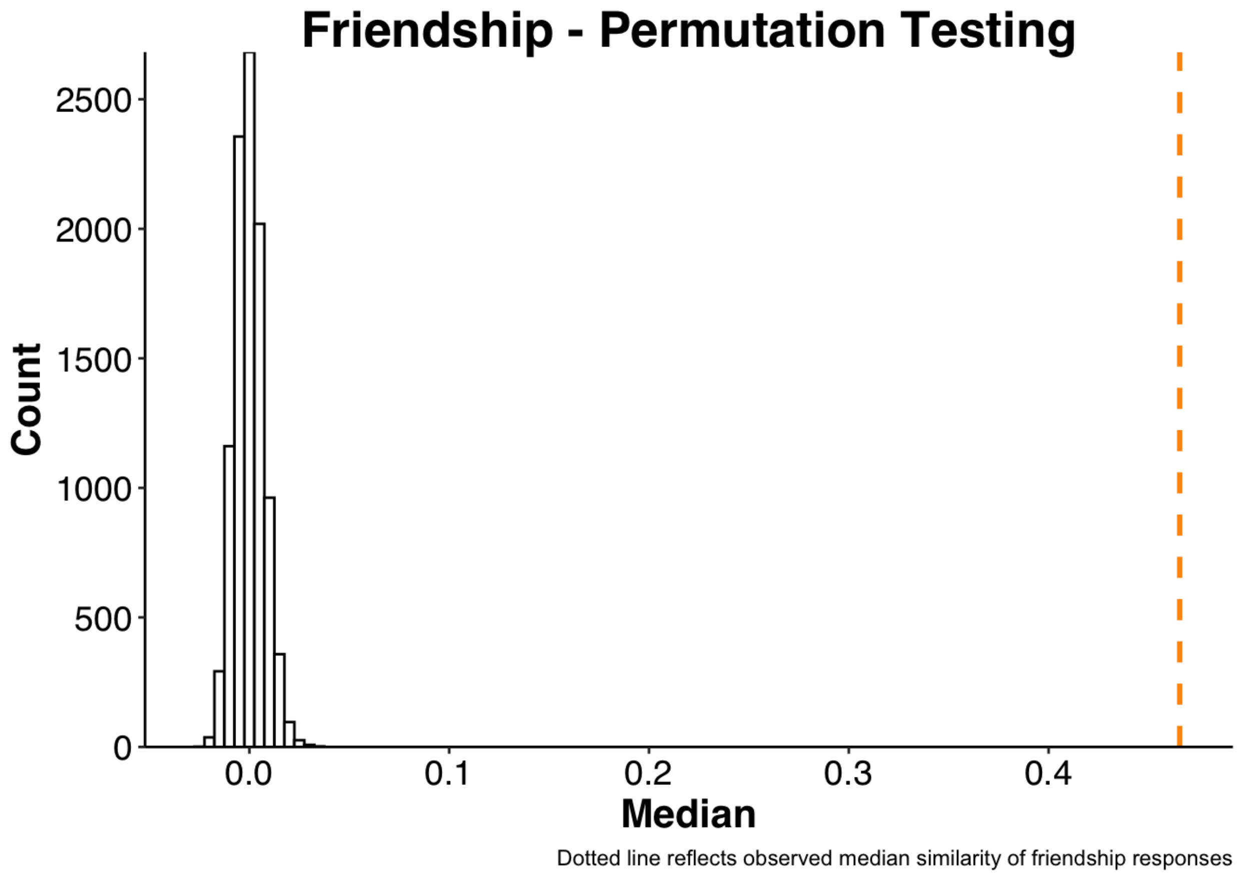

Supp. Fig. 10. Null distribution of median pairwise similarity for friendship responses compared to observed friendship median. Distribution of median pairwise similarity values across 10,000 bootstrapping iterations pictured in white bars and observed median friendship similarity value pictured as dotted orange vertical line.

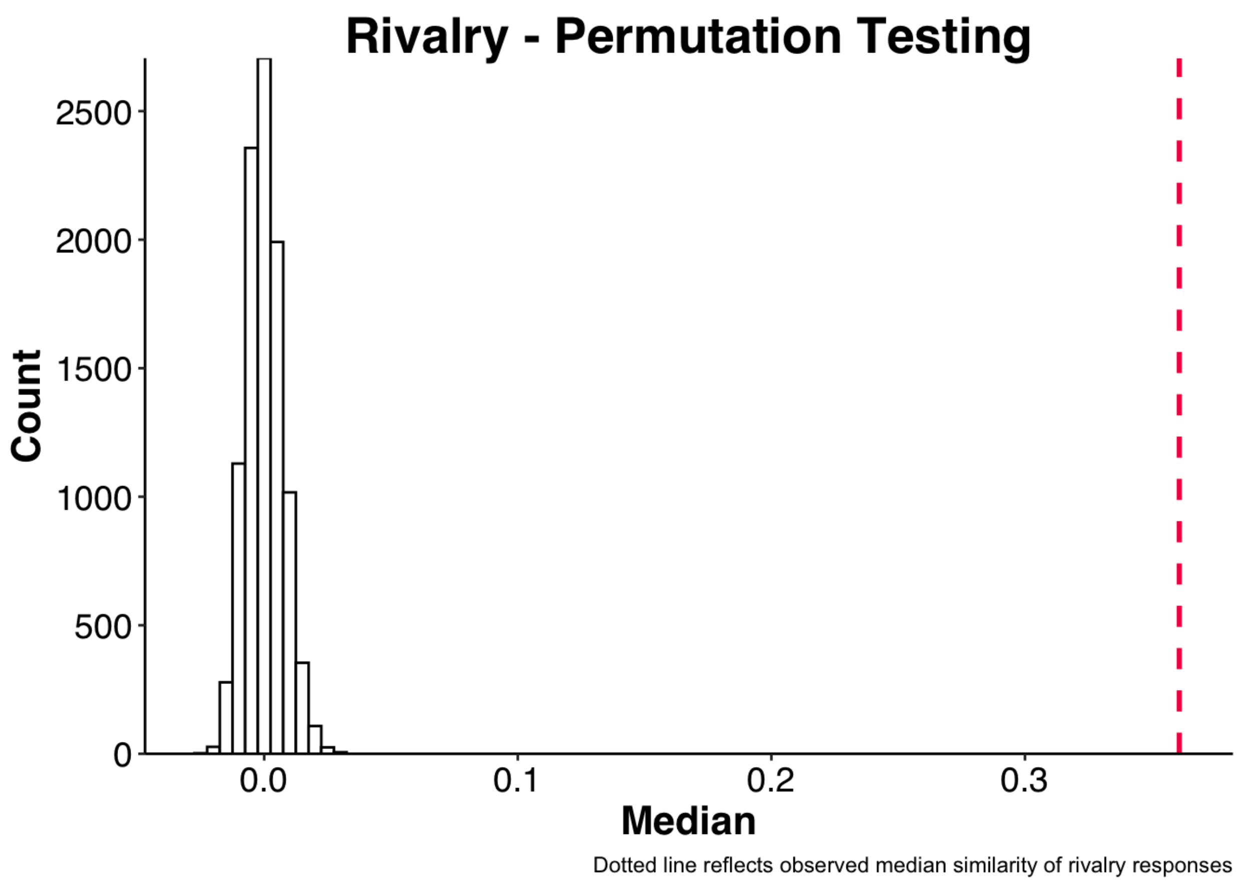

Supp. Fig. 11. Null distribution of median pairwise similarity for rivalry responses compared to observed rivalry median. Distribution of median pairwise similarity values across 10,000 bootstrapping iterations pictured in white bars and observed median rivalry similarity value pictured as dotted red vertical line.

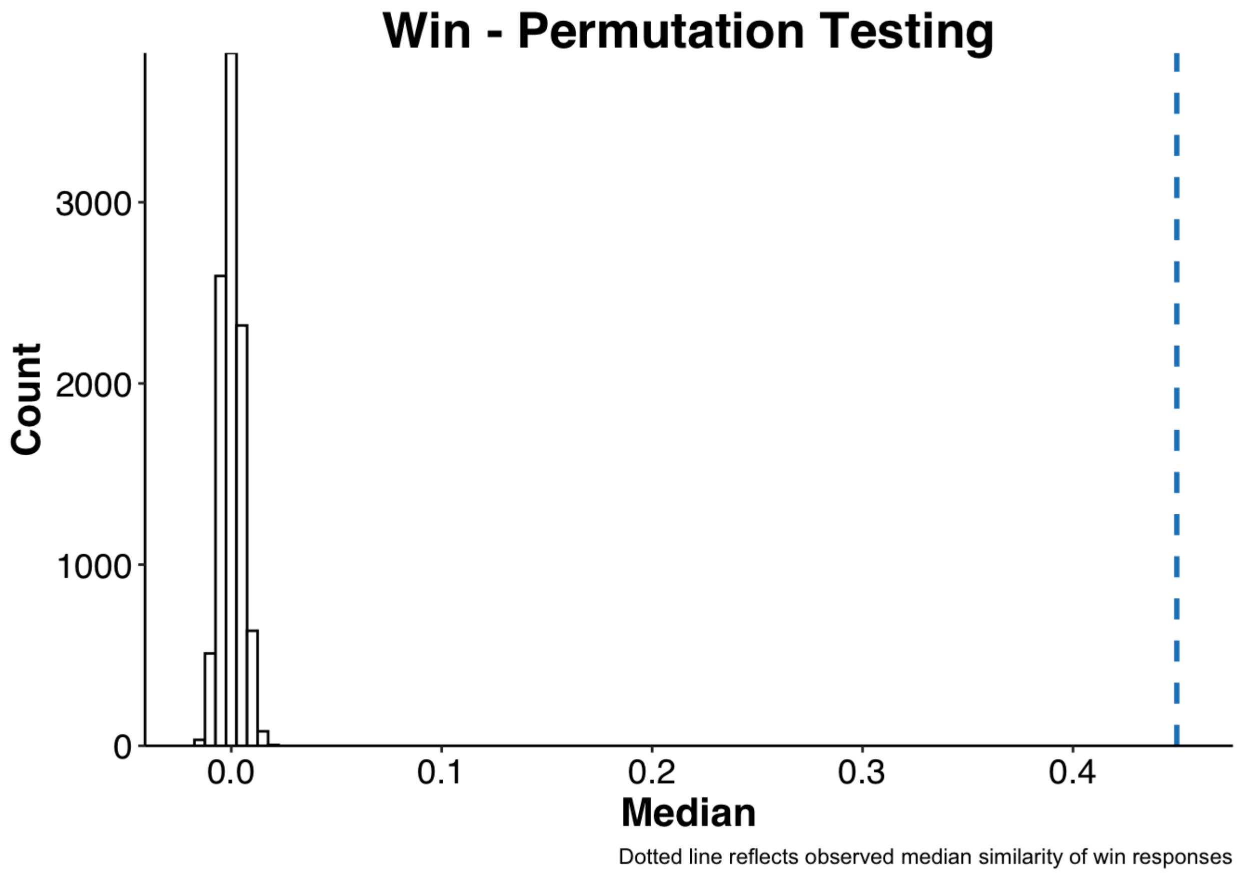

Supp. Fig. 12. Null distribution of median pairwise similarity for win responses compared to observed win median. Distribution of median pairwise similarity values across 10,000 bootstrapping iterations pictured in white bars and observed median win similarity value pictured as dotted blue vertical line.

Conversational Linguistic Features

Sentiment & Clout Conversation Examples

	SENTIMENT	CLOUT
HIGH	"I'm wishing myself luck in the next immunity challenge." "She's so sweet." "We gotta trust in a game like this."	"But I think we keep ourselves strong, us four." "We have the numbers." "They can't hurt us."
LOW	"Shii Ann's an absolutely obnoxious person." "I know I'm not safe, that's for sure." "Stupid people."	"I was thinking that today." "I'll never win." "But we can't beat Rupert."

Supp. Fig. 13. Examples of conversational sentences that are tagged as high vs. low clout by LIWC and as high vs. low sentiment by "sentimentr". High sentiment reflects positive values calculated by "sentimentr" and denotes a positive emotional tone, while low sentiment reflects negative values and denotes a negative emotional tone. High clout reflects values above 50 (in a range of 1-99) calculated by LIWC and denotes high confidence, while low clout reflects values below 50 and denotes low confidence.

Semantic Similarity

Gist Specific

Supp. Table 1. Gist specific semantic similarity. Model estimates and 95% credibility intervals shown for fixed effects. MCMC chain convergence (Rhat) and chain resolution (bulk effective sample size) values shown for fixed effects.

	Estimate	95% CrI [lower, upper]	Rhat	Bulk Effective Sample Size
Intercept	-0.324	[-0.730, 0.071]	1.00094	3576
Gist Specific Similarity	1.583	[0.932, 2.243]	1.00009	17872
Condition (Ref. = Friend)	0.550	[-0.124, 1.233]	1.00096	3659
Gist Specific Similarity * Condition (Ref. = Friend)	-2.500	[-3.502, -1.495]	1.00007	21631

Supp. Table 2. Gist specific semantic similarity marginal effects. Marginal effects for friend and rival conditions and 95% credibility intervals shown.

Condition	Marginal Effect	95% CrI [lower, upper]
Friend	1.582	[0.932, 2.243]
Rival	-0.919	[-1.524, -0.326]

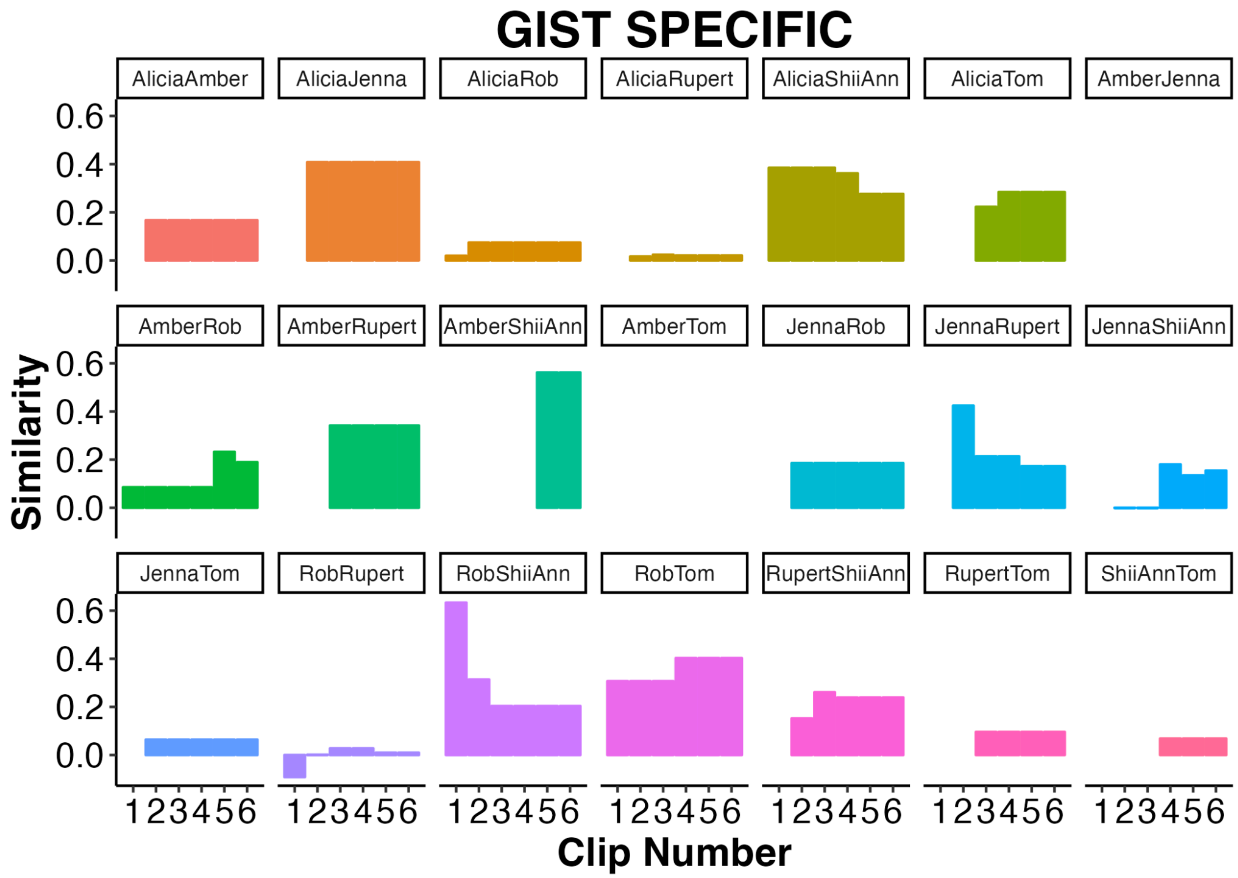

Supp. Fig. 14. Dyadic gist specific semantic similarity across episode clips. Average gist specific similarity per dyad across episode clips. Similarity scores can range from -1 to 1, but typically range from 0 to 1 in this data.

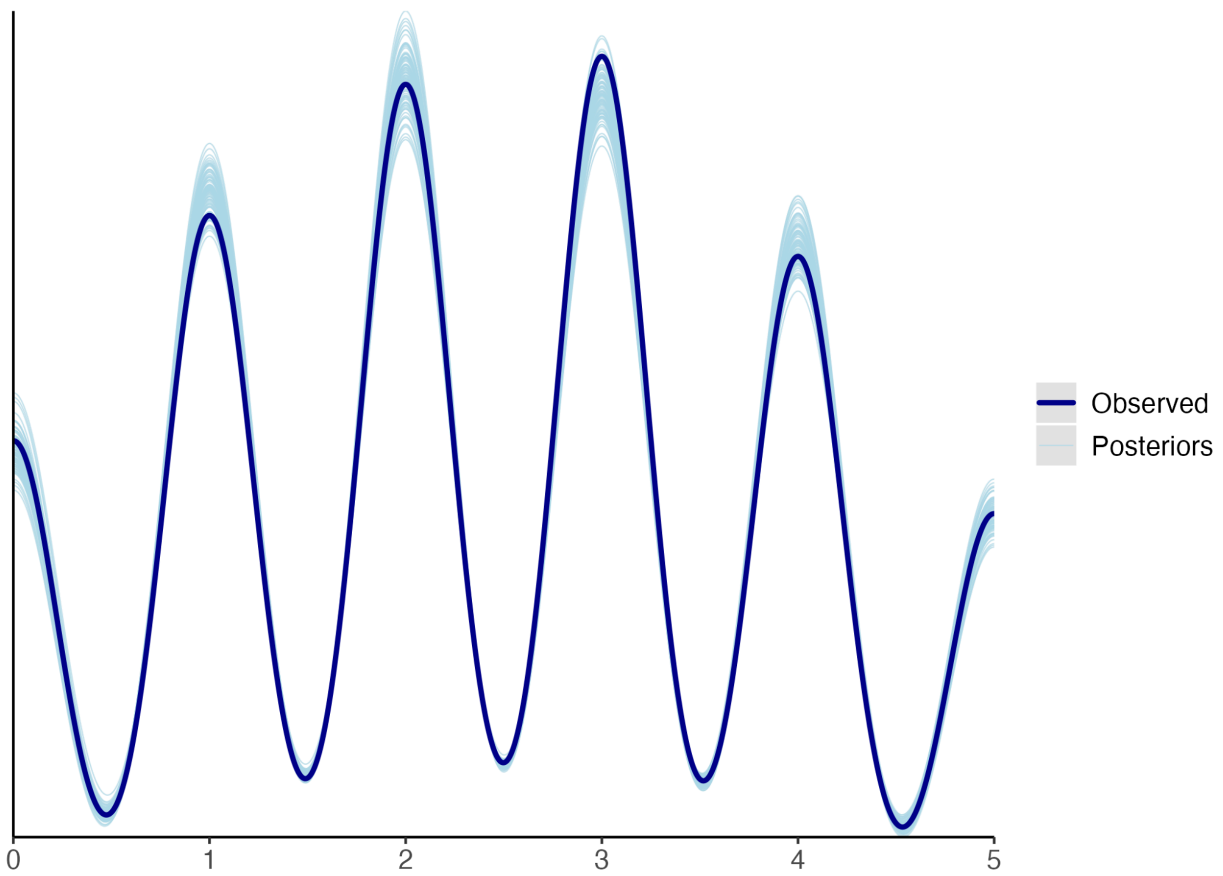

Supp. Fig. 15. Posterior predictive check density plot for gist specific semantic similarity model. Dark blue line shows observed data and light blue lines reflect a sample of 100 posteriors. Due to the ratio of selected to presented responses used to calculate % time chosen, a beta-binomial model with logit link was fit to the data. The waveform pattern of observed data reflects the ratio of integers of selected vs. presented responses. This graph highlights that the simulated data from model posteriors (light blue lines) are an excellent fit to the pattern in the observed data (dark blue line).

Recent Specific

Supp. Table 3. Recent specific semantic similarity. Model estimates and 95% credibility intervals shown for fixed effects. MCMC chain convergence (Rhat) and chain resolution (bulk effective sample size) values shown for fixed effects.

	Estimate	95% CrI [lower, upper]	Rhat	Bulk Effective Sample Size
Intercept	0.031	[-0.354, 0.408]	1.00179	4750
Recent Specific Similarity	0.191	[-0.288, 0.670]	1.00017	23245
Condition (Ref. = Friend)	-0.027	[-0.695, 0.662]	1.00197	4719
Recent Specific Similarity * Condition (Ref. = Friend)	-0.114	[-0.846, 0.616]	1.00002	24597

Supp. Table 4. Recent specific semantic similarity marginal effects. Marginal effects for friend and rival conditions and 95% credibility intervals shown.

Condition	Marginal Effect	95% CrI [lower, upper]
Friend	0.190	[-0.297, 0.660]
Rival	0.075	[-0.394, 0.562]

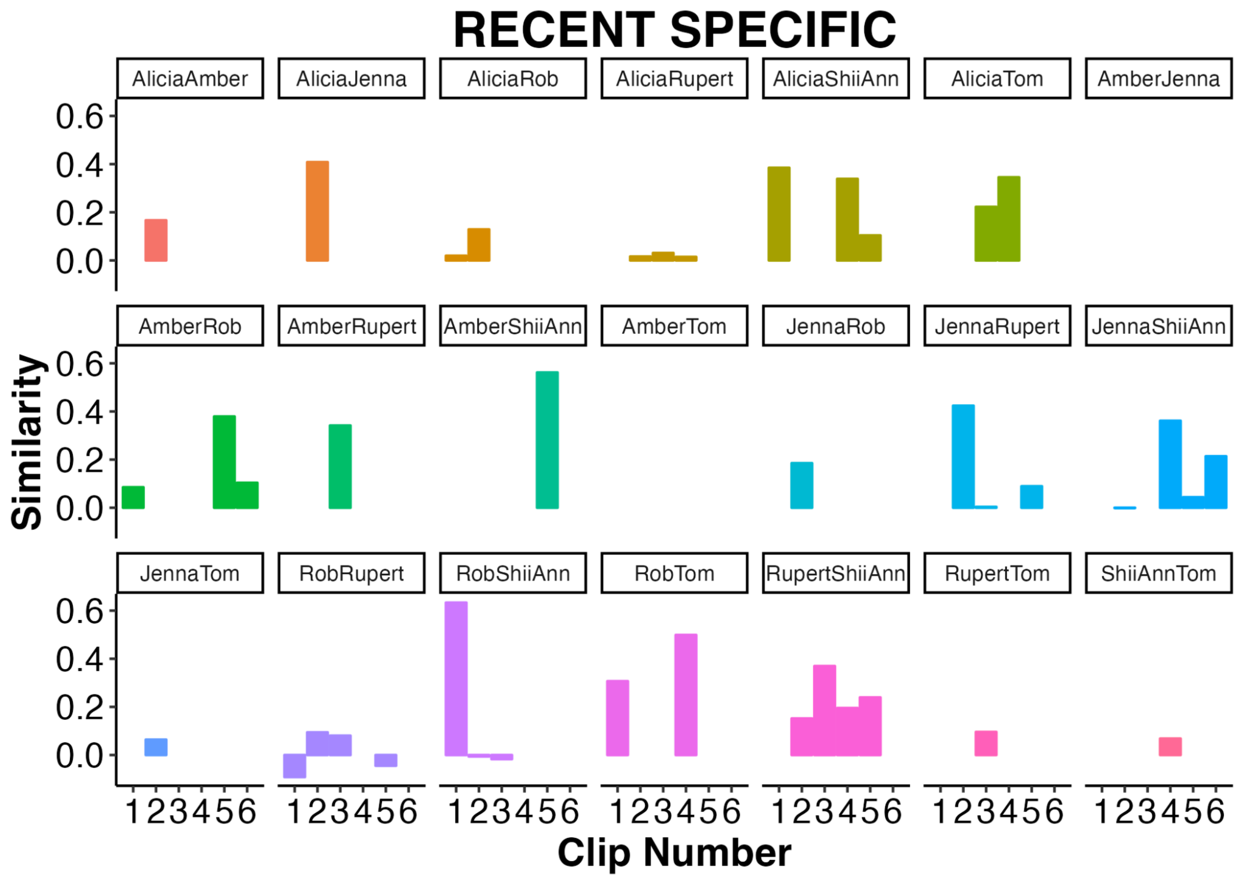

Supp. Fig. 16. Dyadic recent specific semantic similarity across episode clips. Average recent specific similarity per dyad across episode clips. Similarity scores can range from -1 to 1, but typically range from 0 to 1 in this data.

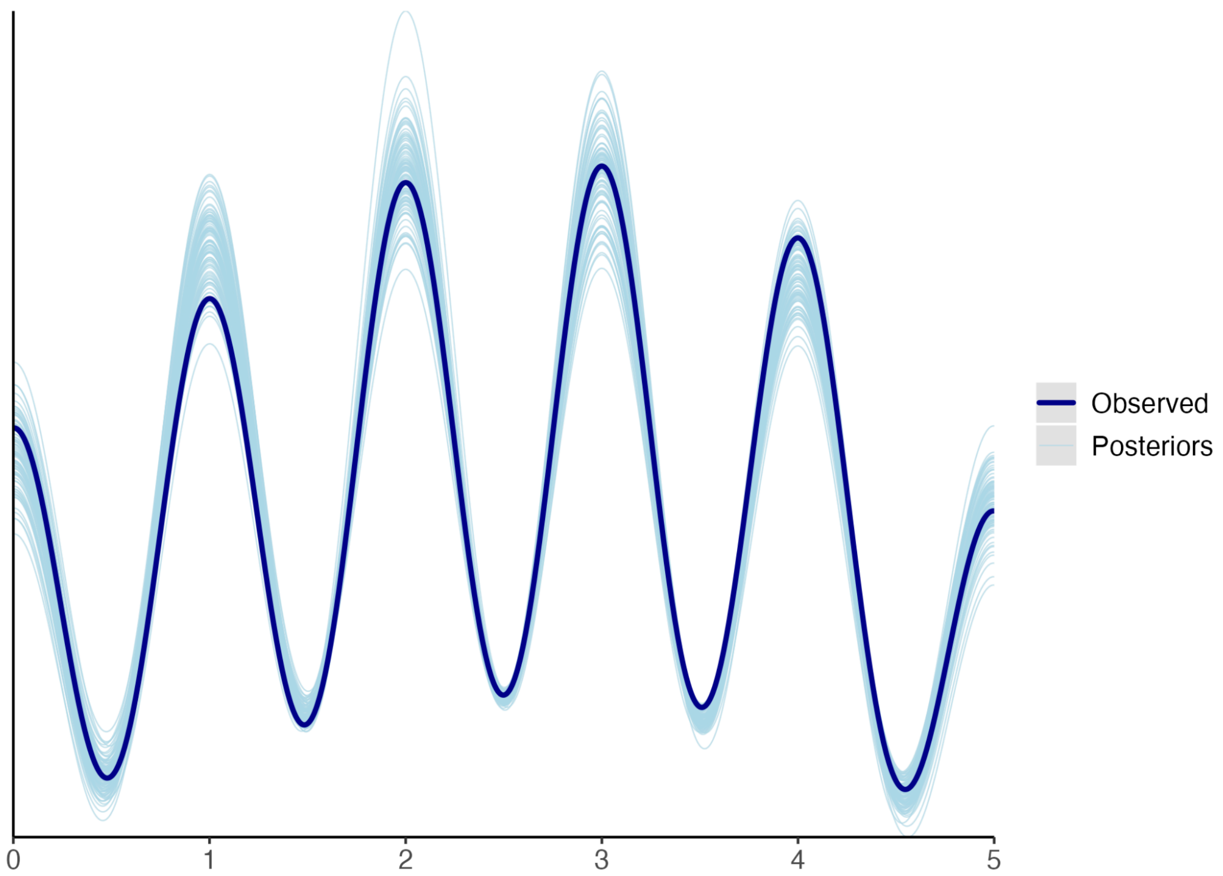

Supp. Fig. 17. Posterior predictive check density plot for recent specific semantic similarity model. Dark blue line shows observed data and light blue lines reflect a sample of 100 posteriors. Due to the ratio of selected to presented responses used to calculate % time chosen, a beta-binomial model with logit link was fit to the data. The waveform pattern of observed data reflects the ratio of integers of selected vs. presented responses. This graph highlights that the simulated data from model posteriors (light blue lines) are an excellent fit to the pattern in the observed data (dark blue line).

Gist General

Supp. Table 5. Gist general semantic similarity. Model estimates and 95% credibility intervals shown for fixed effects. MCMC chain convergence (Rhat) and chain resolution (bulk effective sample size) values shown for fixed effects.

	Estimate	95% CrI [lower, upper]	Rhat	Bulk Effective Sample Size
Intercept	0.086	[-0.283, 0.456]	1.00036	3842
Gist General Similarity	-0.260	[-0.883, 0.369]	1.00005	23127
Condition (Ref. = Friend)	-0.376	[-0.985, 0.221]	1.00040	3776
Gist General Similarity * Condition (Ref. = Friend)	1.134	[0.194, 2.066]	1.00000	25270

Supp. Table 6. Gist general semantic similarity marginal effects. Marginal effects for friend and rival conditions and 95% credibility intervals shown.

Condition	Marginal Effect	95% CrI [lower, upper]
Friend	-0.261	[-0.869, 0.381]
Rival	0.874	[0.236, 1.516]

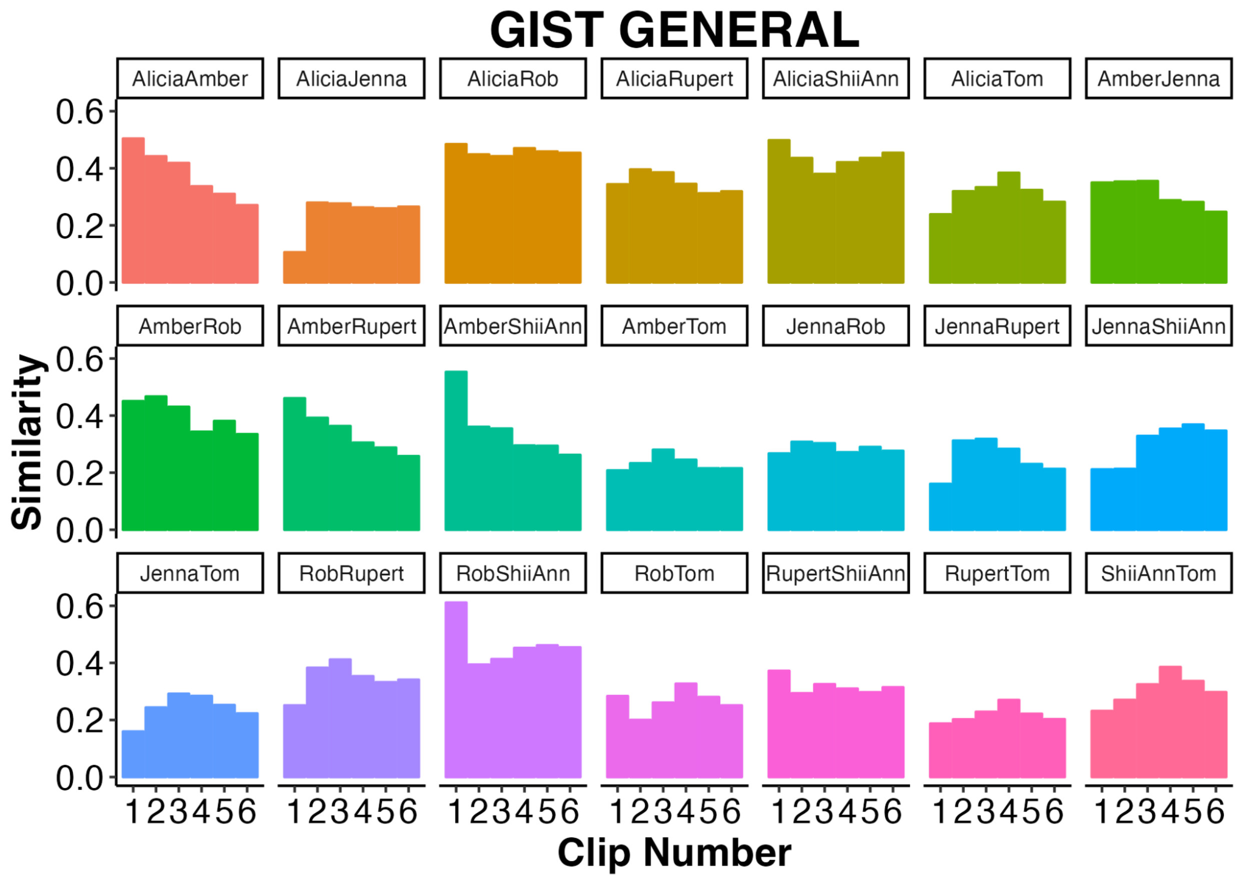

Supp. Fig. 18. Dyadic gist general semantic similarity across episode clips. Average gist general similarity per dyad across episode clips. Similarity scores can range from -1 to 1, but typically range from 0 to 1 in this data.

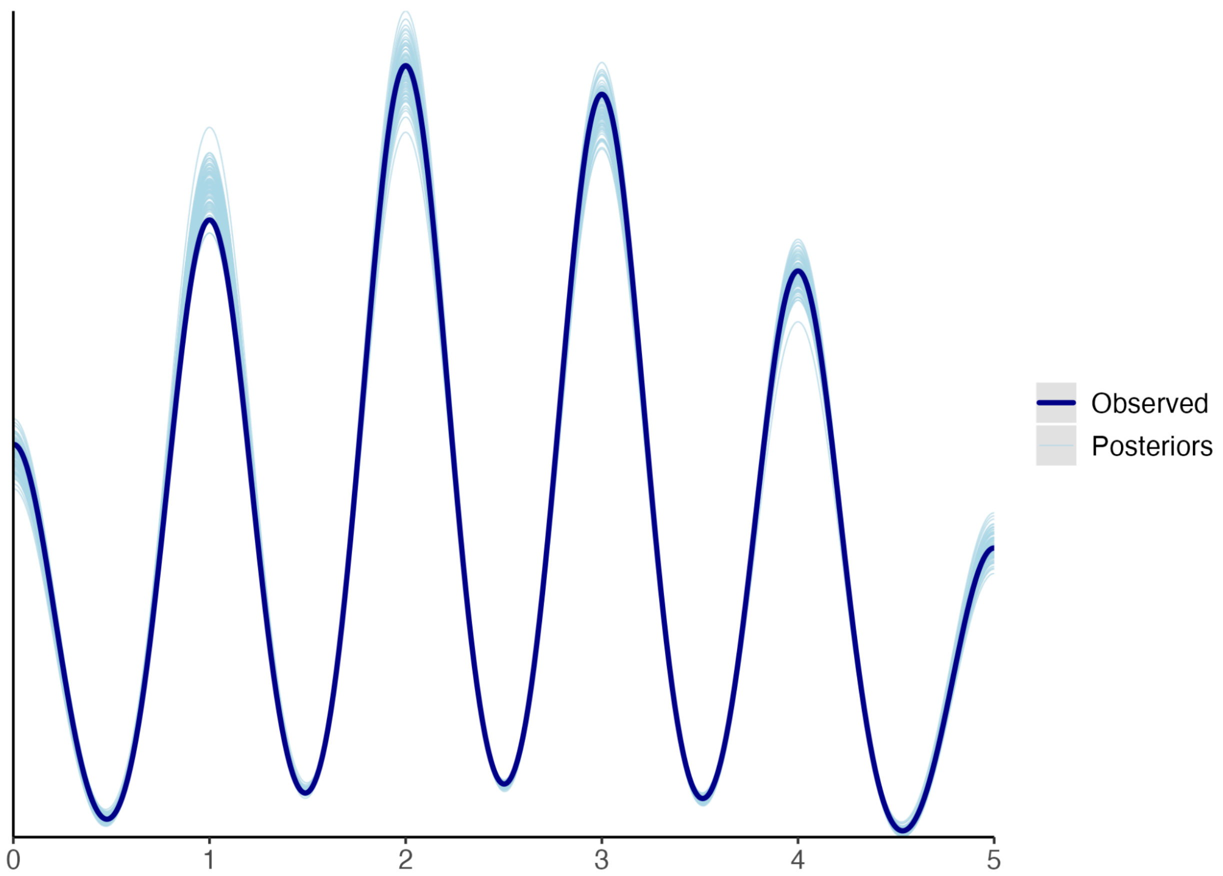

Supp. Fig. 19. Posterior predictive check density plot for gist general semantic similarity model. Dark blue line shows observed data and light blue lines reflect a sample of 100 posteriors. Due to the ratio of selected to presented responses used to calculate % time chosen, a beta-binomial model with logit link was fit to the data. The waveform pattern of observed data reflects the ratio of integers of selected vs. presented responses. This graph highlights that the simulated data from model posteriors (light blue lines) are an excellent fit to the pattern in the observed data (dark blue line).

Recent General

Supp. Table 7. Recent general semantic similarity. Model estimates and 95% credibility intervals shown for fixed effects. MCMC chain convergence (Rhat) and chain resolution (bulk effective sample size) values shown for fixed effects.

	Estimate	95% CrI [lower, upper]	Rhat	Bulk Effective Sample Size
Intercept	0.077	[-0.233, 0.387]	1.00257	3242
Recent General Similarity	-0.254	[-0.519, 0.012]	1.00019	35343
Condition (Ref. = Friend)	-0.185	[-0.711, 0.341]	1.00259	3359
Recent General Similarity * Condition (Ref. = Friend)	0.606	[0.239, 0.975]	1.00006	37007

Supp. Table 8. Recent general semantic similarity marginal effects. Marginal effects for friend and rival conditions and 95% credibility intervals shown.

Condition	Marginal Effect	95% CrI [lower, upper]
Friend	-0.254	[-0.516, 0.014]
Rival	0.353	[0.089, 0.596]

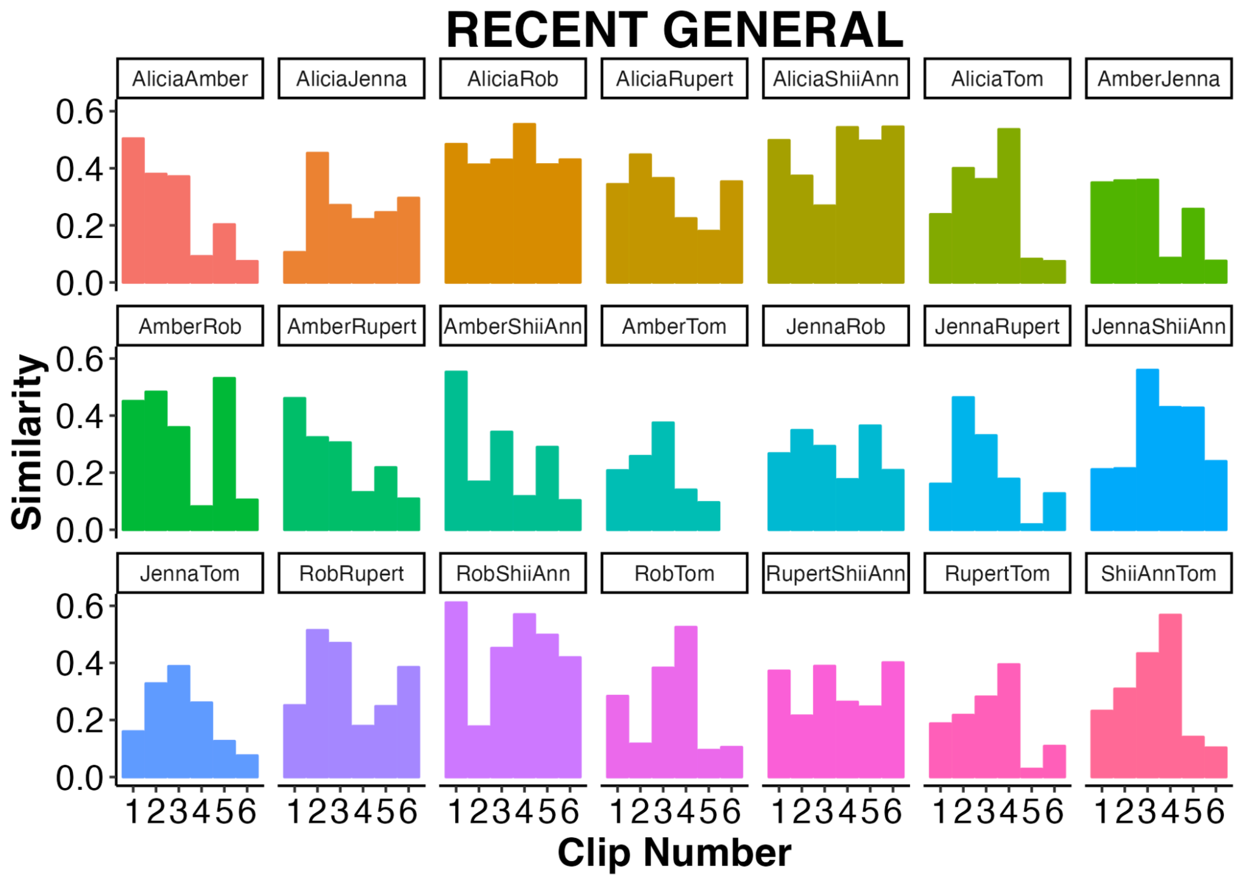

Supp. Fig. 20. Dyadic recent general semantic similarity across episode clips. Average recent general similarity per dyad across episode clips. Similarity scores can range from -1 to 1, but typically range from 0 to 1 in this data.

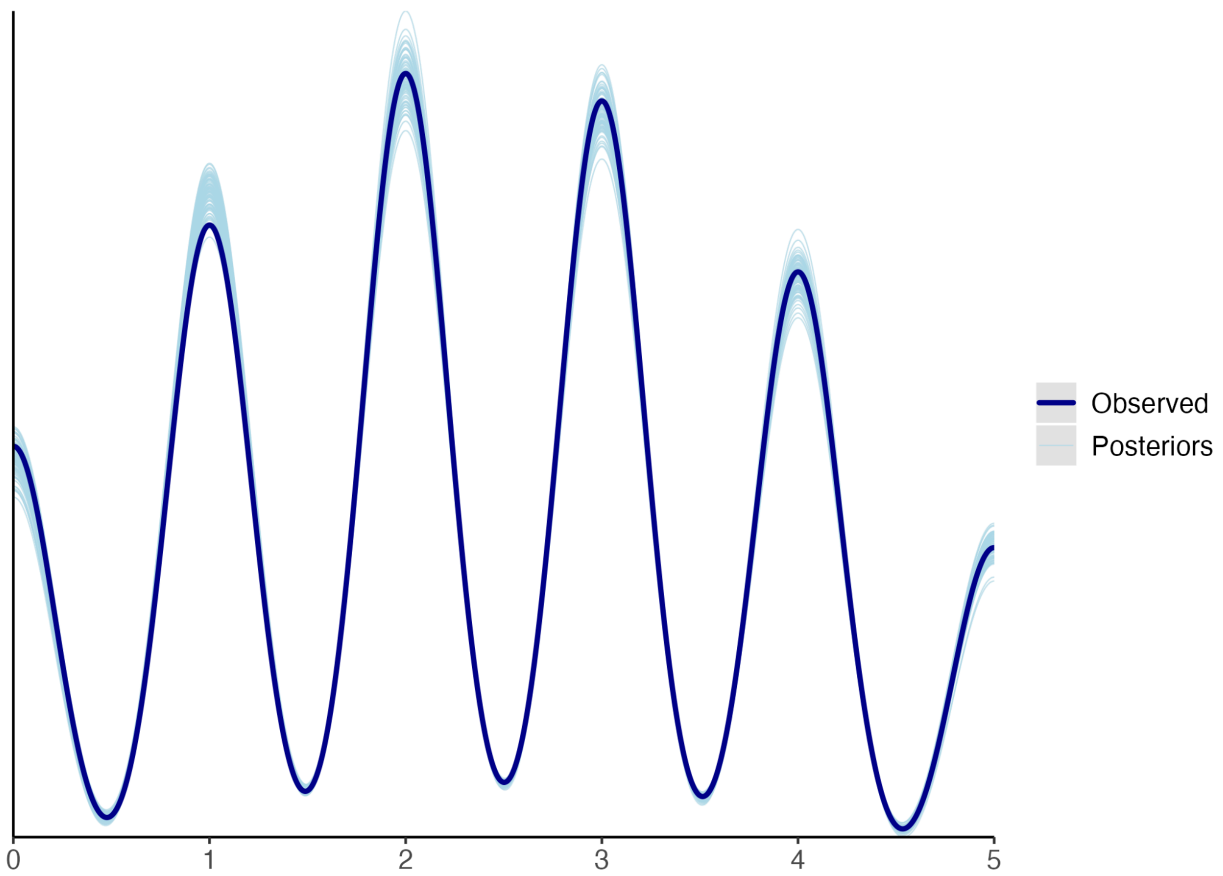

Supp. Fig. 21. Posterior predictive check density plot for recent general semantic similarity model. Dark blue line shows observed data and light blue lines reflect a sample of 100 posteriors. Due to the ratio of selected to presented responses used to calculate % time chosen, a beta-binomial model with logit link was fit to the data. The waveform pattern of observed data reflects the ratio of integers of selected vs. presented responses. This graph highlights that the simulated data from model posteriors (light blue lines) are an excellent fit to the pattern in the observed data (dark blue line).

Sentiment

Gist Specific

Supp. Table 9. Gist specific sentiment. Model estimates and 95% credibility intervals shown for fixed effects. MCMC chain convergence (Rhat) and chain resolution (bulk effective sample size) values shown for fixed effects.

	Estimate	95% CrI [lower, upper]	Rhat	Bulk Effective Sample Size
Intercept	-0.008	[-0.391, 0.382]	1.00090	4350
Gist Specific Sentiment	0.564	[0.274, 0.855]	1.00001	25867
Condition (Ref. = Friend)	0.040	[-0.628, 0.696]	1.00075	4484
Gist Specific Sentiment * Condition (Ref. = Friend)	-0.996	[-1.425, -0.570]	1.00000	32138

Supp. Table 10. Gist specific sentiment marginal effects. Marginal effects for friend and rival conditions and 95% credibility intervals shown.

Condition	Marginal Effect	95% CrI [lower, upper]
Friend	0.563	[0.277, 0.857]
Rival	-0.432	[-0.696, -0.160]

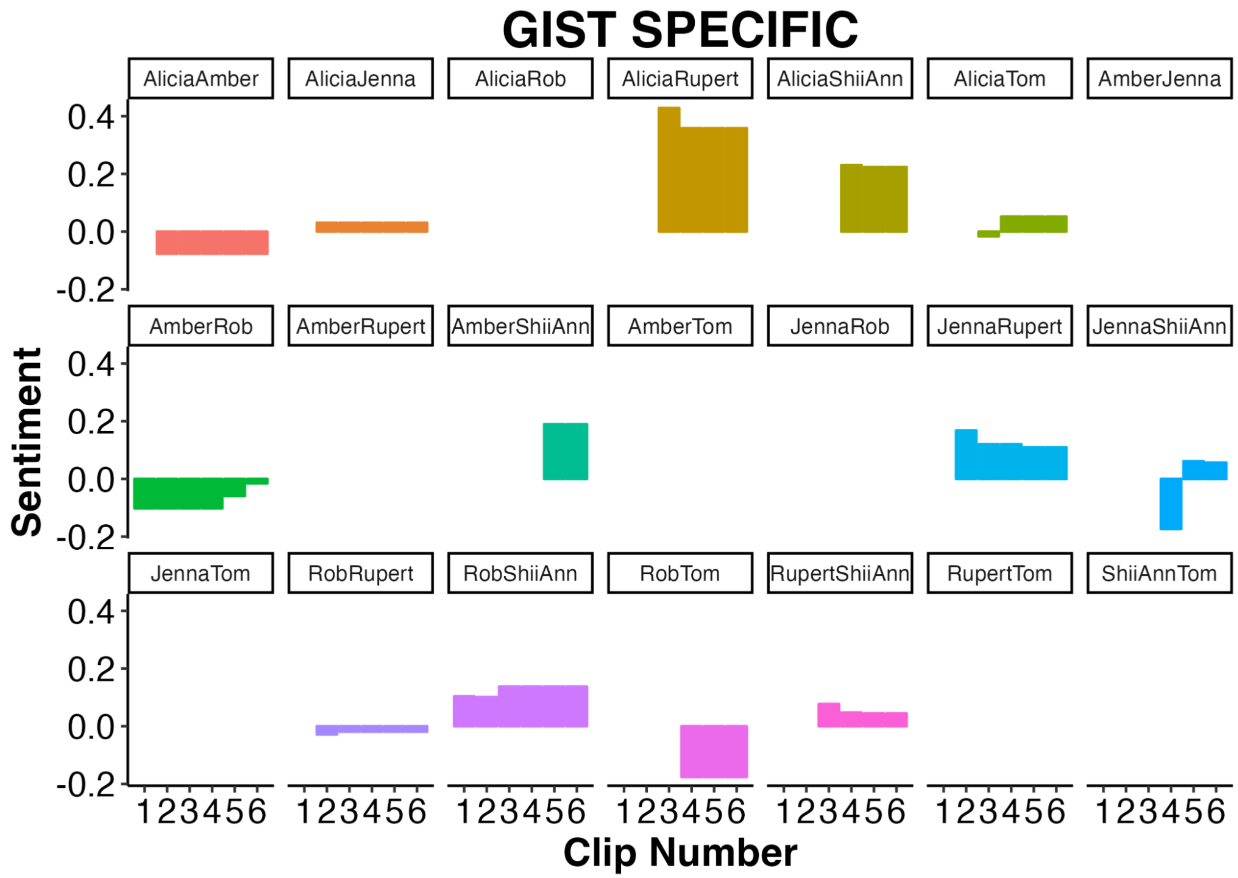

Supp. Fig. 22. Dyadic gist specific sentiment across episode clips. Average gist specific sentiment per dyad across episode clips. Sentiment scores are unbounded.

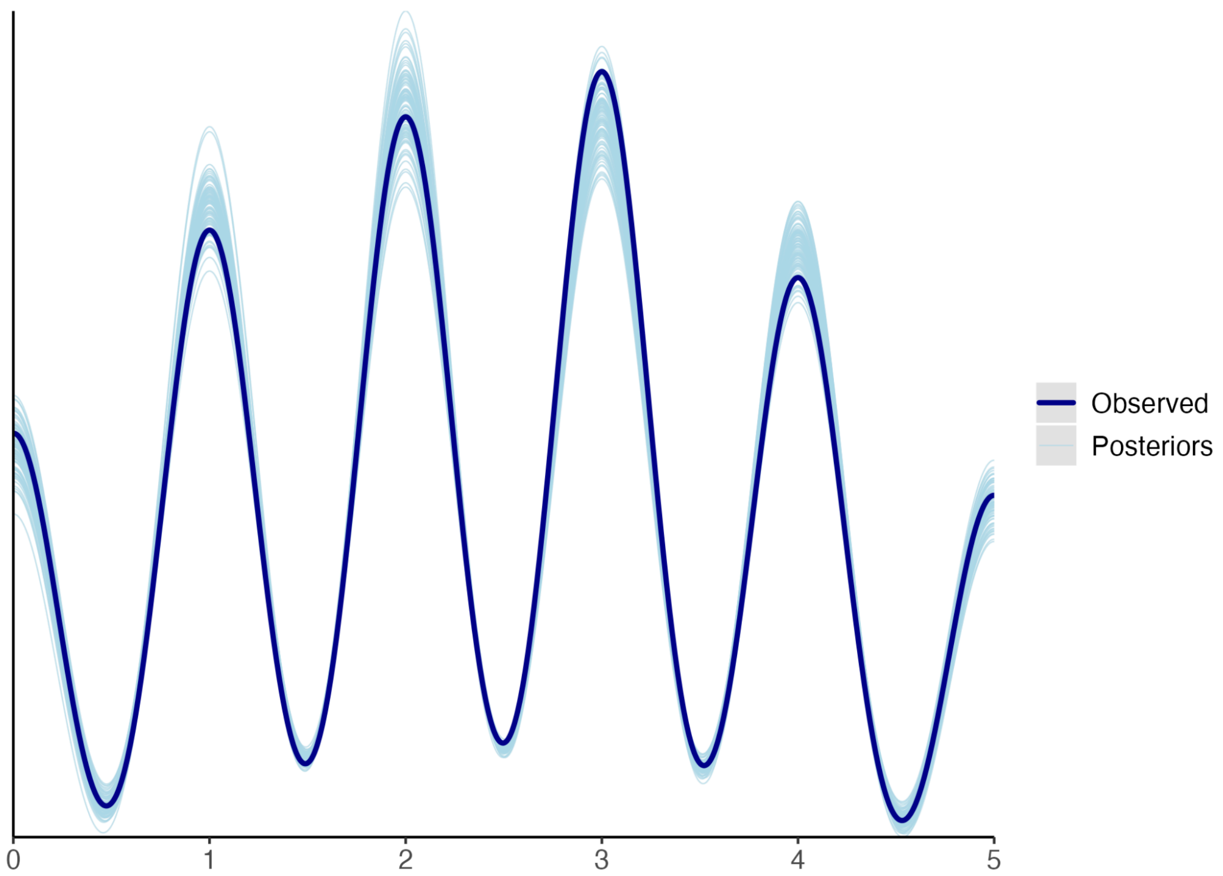

Supp. Fig. 23. Posterior predictive check density plot for gist specific sentiment model. Dark blue line shows observed data and light blue lines reflect a sample of 100 posteriors. Due to the ratio of selected to presented responses used to calculate % time chosen, a beta-binomial model with logit link was fit to the data. The waveform pattern of observed data reflects the ratio of integers of selected vs. presented responses. This graph highlights that the simulated data from model posteriors (light blue lines) are an excellent fit to the pattern in the observed data (dark blue line).

Recent Specific

Supp. Table 11. Recent specific sentiment. Model estimates and 95% credibility intervals shown for fixed effects. MCMC chain convergence (Rhat) and chain resolution (bulk effective sample size) values shown for fixed effects.

	Estimate	95% CrI [lower, upper]	Rhat	Bulk Effective Sample Size
Intercept	0.123	[-0.281, 0.527]	1.00086	9071
Recent Specific Sentiment	-0.023	[-0.373, 0.328]	1.00019	25418
Condition (Ref. = Friend)	-0.140	[-0.845, 0.566]	1.00087	9348
Recent Specific Sentiment * Condition (Ref. = Friend)	0.156	[-0.371, 0.689]	1.00014	25500

Supp. Table 12. Recent specific sentiment marginal effects. Marginal effects for friend and rival conditions and 95% credibility intervals shown.

Condition	Marginal Effect	95% CrI [lower, upper]
Friend	-0.023	[-0.367, 0.333]
Rival	0.132	[-0.215, 0.487]

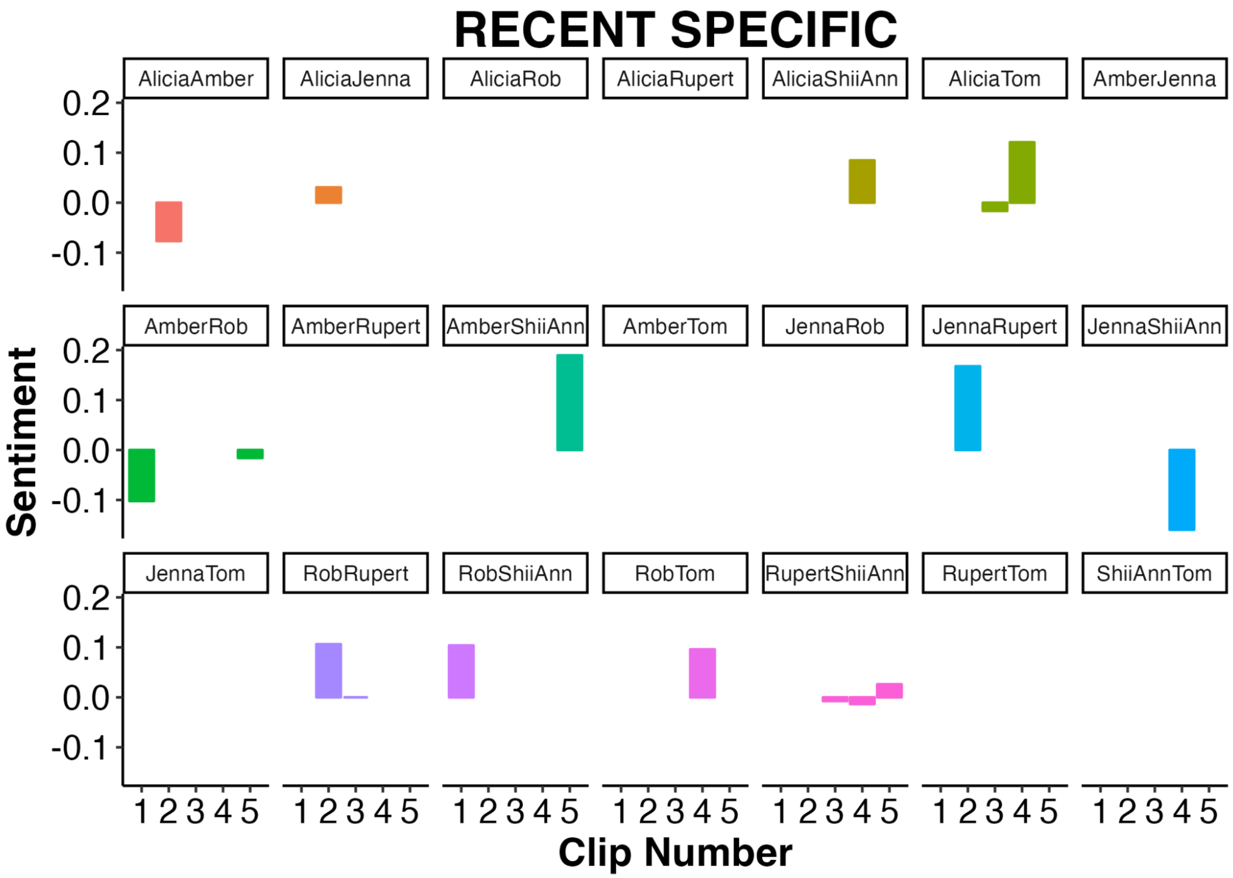

Supp. Fig. 24. Dyadic recent specific sentiment across episode clips. Average recent specific sentiment per dyad across episode clips. Sentiment scores are unbounded.

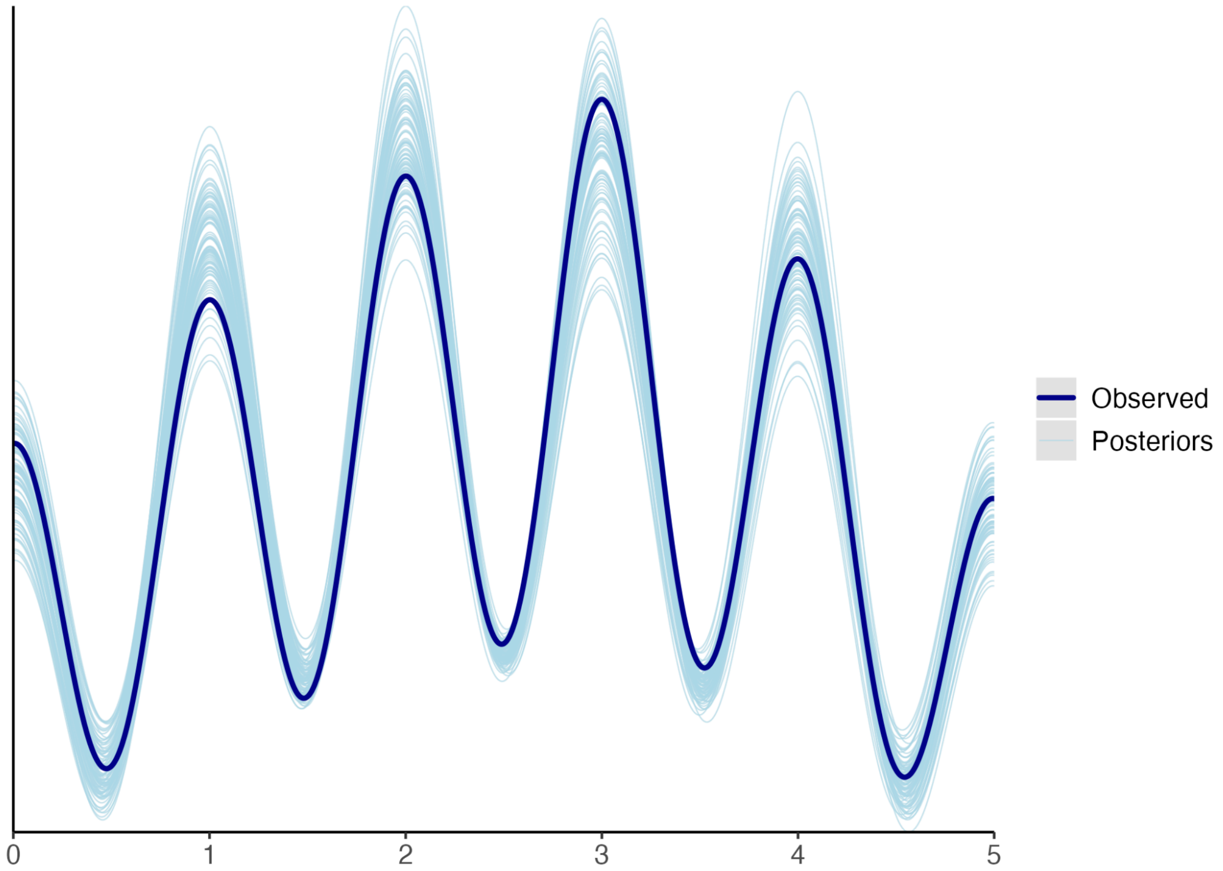

Supp. Fig. 25. Posterior predictive check density plot for recent specific sentiment model. Dark blue line shows observed data and light blue lines reflect a sample of 100 posteriors. Due to the ratio of selected to presented responses used to calculate % time chosen, a beta-binomial model with logit link was fit to the data. The waveform pattern of observed data reflects the ratio of integers of selected vs. presented responses. This graph highlights that the simulated data from model posteriors (light blue lines) are an excellent fit to the pattern in the observed data (dark blue line).

Gist General

Supp. Table 13. Gist general sentiment. Model estimates and 95% credibility intervals shown for fixed effects. MCMC chain convergence (Rhat) and chain resolution (bulk effective sample size) values shown for fixed effects.

	Estimate	95% CrI [lower, upper]	Rhat	Bulk Effective Sample Size
Intercept	-0.119	[-0.430, 0.193]	1.00067	3405
Gist General Sentiment	1.421	[0.893, 1.946]	0.99998	26561
Condition (Ref. = Friend)	0.108	[-0.416, 0.638]	1.00055	6888
Gist General Sentiment * Condition (Ref. = Friend)	-1.335	[-2.086, -0.567]	0.99999	25309

Supp. Table 14. Gist general sentiment marginal effects. Marginal effects for friend and rival conditions and 95% credibility intervals shown.

Condition	Marginal Effect	95% CrI [lower, upper]
Friend	1.423	[0.897, 1.949]
Rival	0.084	[-0.446, 0.628]

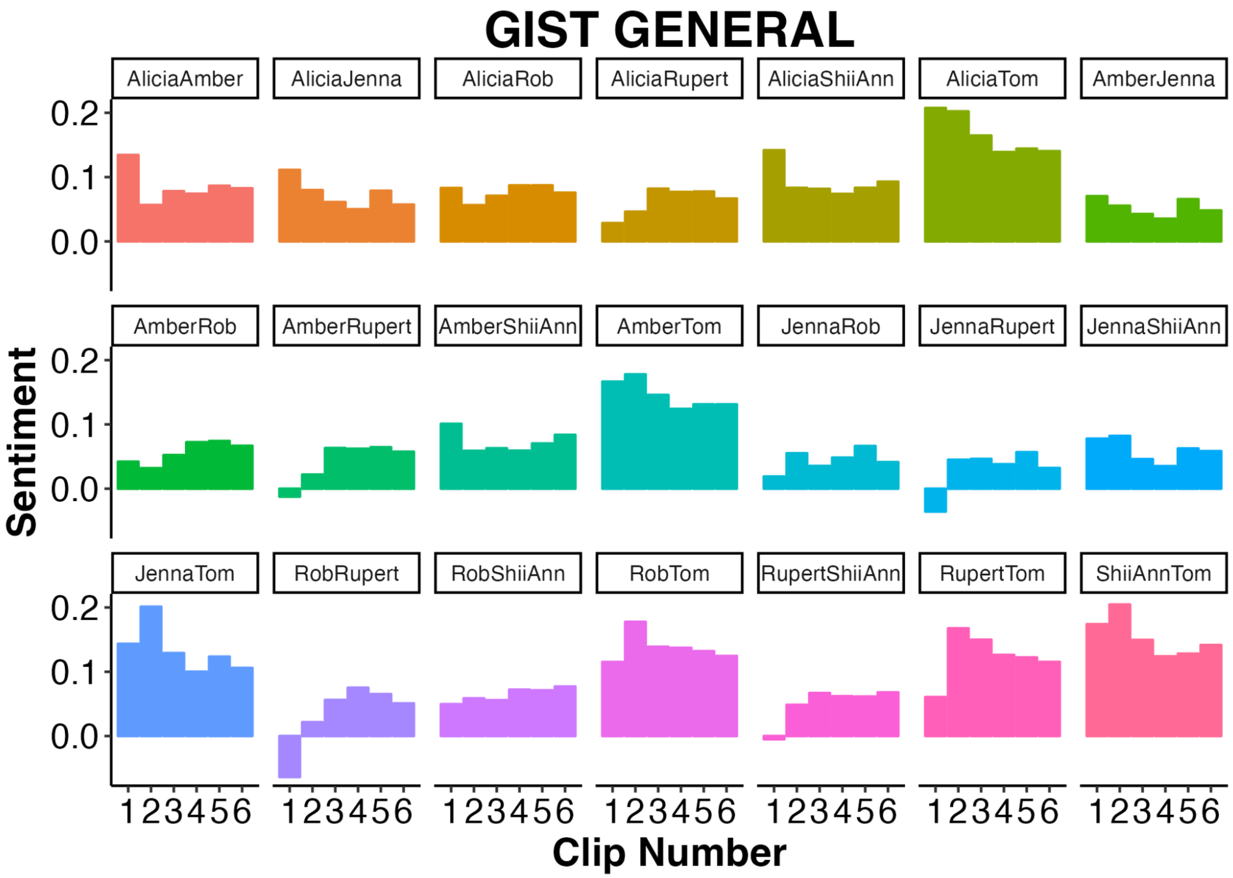

Supp. Fig. 26. Dyadic gist general sentiment across episode clips. Average gist general sentiment per dyad across episode clips. Sentiment scores are unbounded.

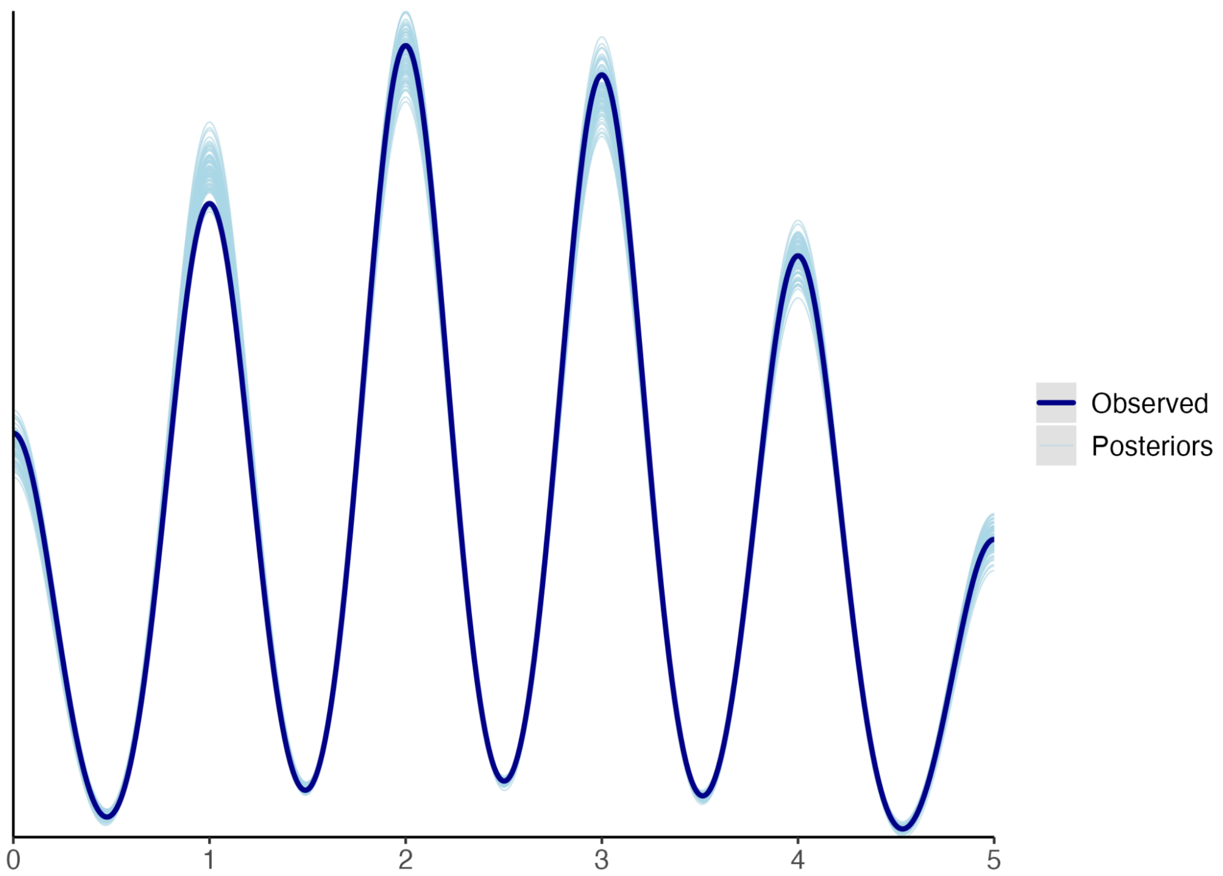

Supp. Fig. 27. Posterior predictive check density plot for gist general sentiment model. Dark blue line shows observed data and light blue lines reflect a sample of 100 posteriors. Due to the ratio of selected to presented responses used to calculate % time chosen, a beta-binomial model with logit link was fit to the data. The waveform pattern of observed data reflects the ratio of integers of selected vs. presented responses. This graph highlights that the simulated data from model posteriors (light blue lines) are an excellent fit to the pattern in the observed data (dark blue line).

Recent General

Supp. Table 15. Recent general sentiment. Model estimates and 95% credibility intervals shown for fixed effects. MCMC chain convergence (Rhat) and chain resolution (bulk effective sample size) values shown for fixed effects.

	Estimate	95% CrI [lower, upper]	Rhat	Bulk Effective Sample Size
Intercept	-0.030	[-0.326, 0.262]	1.00066	4155
Recent General Sentiment	0.528	[0.193, 0.867]	1.00013	42787
Condition (Ref. = Friend)	0.010	[-0.502, 0.532]	1.00050	4359
Recent General Sentiment * Condition (Ref. = Friend)	-0.681	[-1.163, -0.202]	0.99997	43081

Supp. Table 16. Recent general sentiment marginal effects. Marginal effects for friend and rival conditions and 95% credibility intervals shown.

Condition	Marginal Effect	95% CrI [lower, upper]
Friend	0.527	[0.191, 0.865]
Rival	-0.153	[-0.483, 0.194]

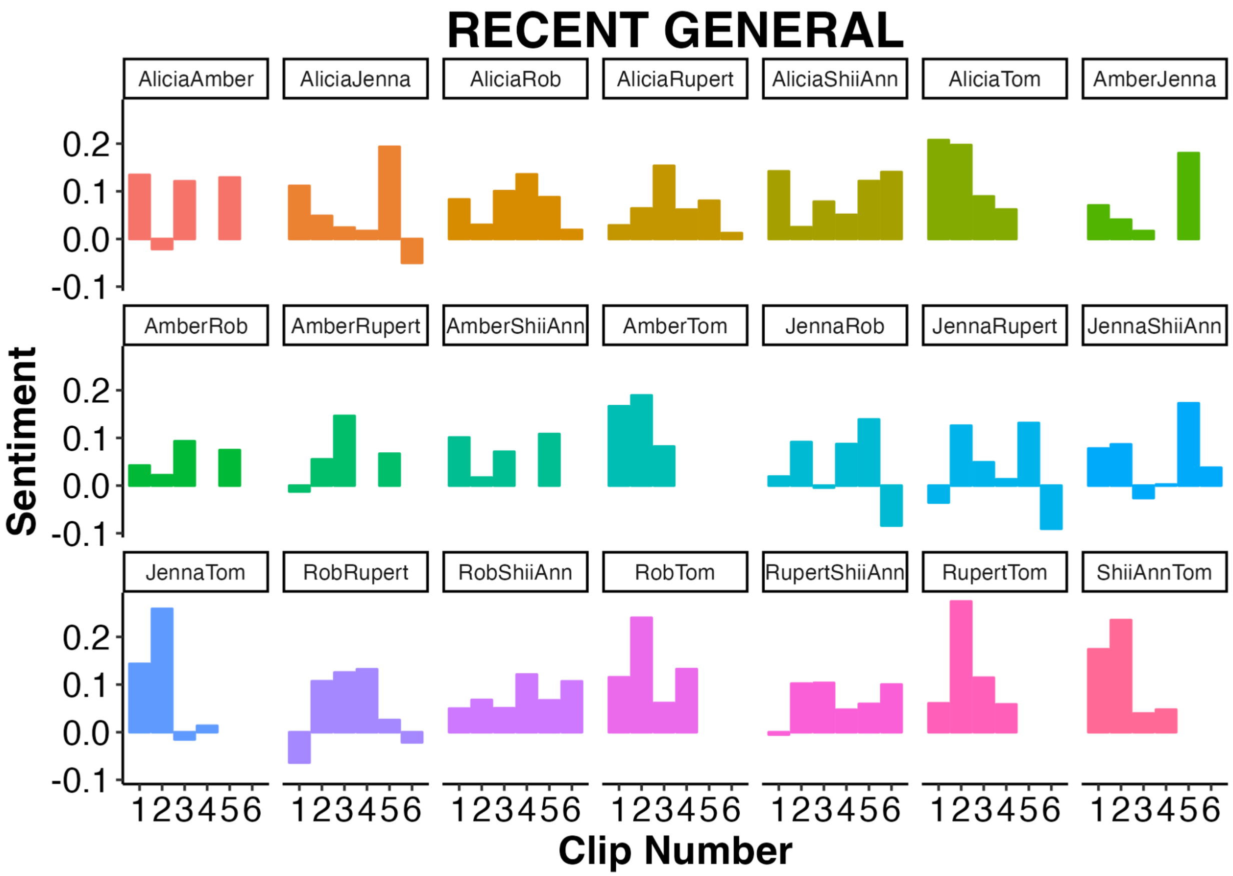

Supp. Fig. 28. Dyadic recent general sentiment across episode clips. Average recent general sentiment per dyad across episode clips. Sentiment scores are unbounded.

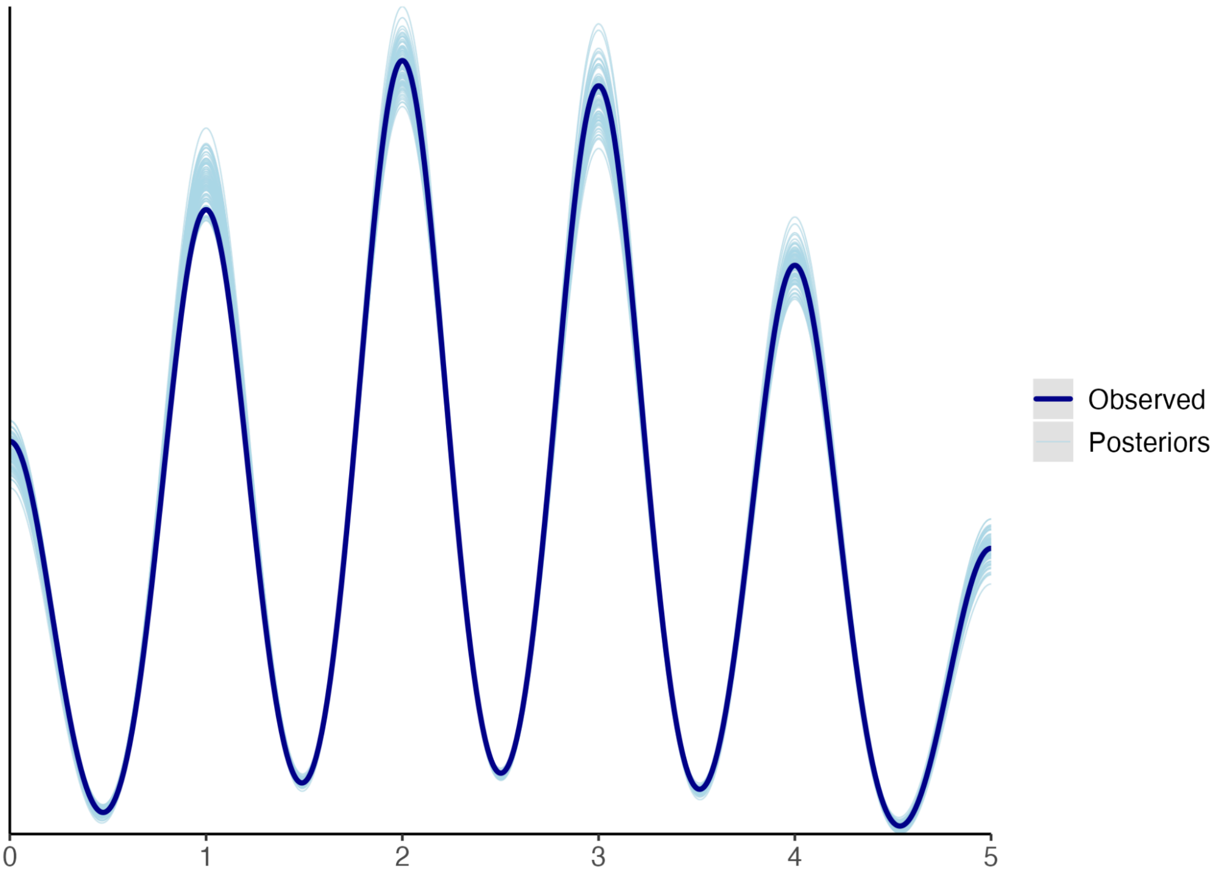

Supp. Fig. 29. Posterior predictive check density plot for recent general sentiment model. Dark blue line shows observed data and light blue lines reflect a sample of 100 posteriors. Due to the ratio of selected to presented responses used to calculate % time chosen, a beta-binomial model with logit link was fit to the data. The waveform pattern of observed data reflects the ratio of integers of selected vs. presented responses. This graph highlights that the simulated data from model posteriors (light blue lines) are an excellent fit to the pattern in the observed data (dark blue line).

Clout

Gist Specific

Supp. Table 17. Gist specific clout. Model estimates and 95% credibility intervals shown for fixed effects. MCMC chain convergence (Rhat) and chain resolution (bulk effective sample size) values shown for fixed effects.

	Estimate	95% CrI [lower, upper]	Rhat	Bulk Effective Sample Size
Intercept	-0.495	[-0.899, -0.090]	1.00112	4756
Gist Specific Clout	0.011	[0.008, 0.014]	1.00020	54536
Condition (Ref. = Friend)	0.793	[0.121, 1.463]	1.00087	4827
Gist Specific Clout * Condition (Ref. = Friend)	-0.018	[-0.022, -0.014]	1.00010	68960

Supp. Table 18. Gist specific clout marginal effects. Marginal effects for friend and rival conditions and 95% credibility intervals shown.

Condition	Marginal Effect	95% CrI [lower, upper]
Friend	0.011	[0.008, 0.014]
Rival	-0.006	[-0.009, -0.004]

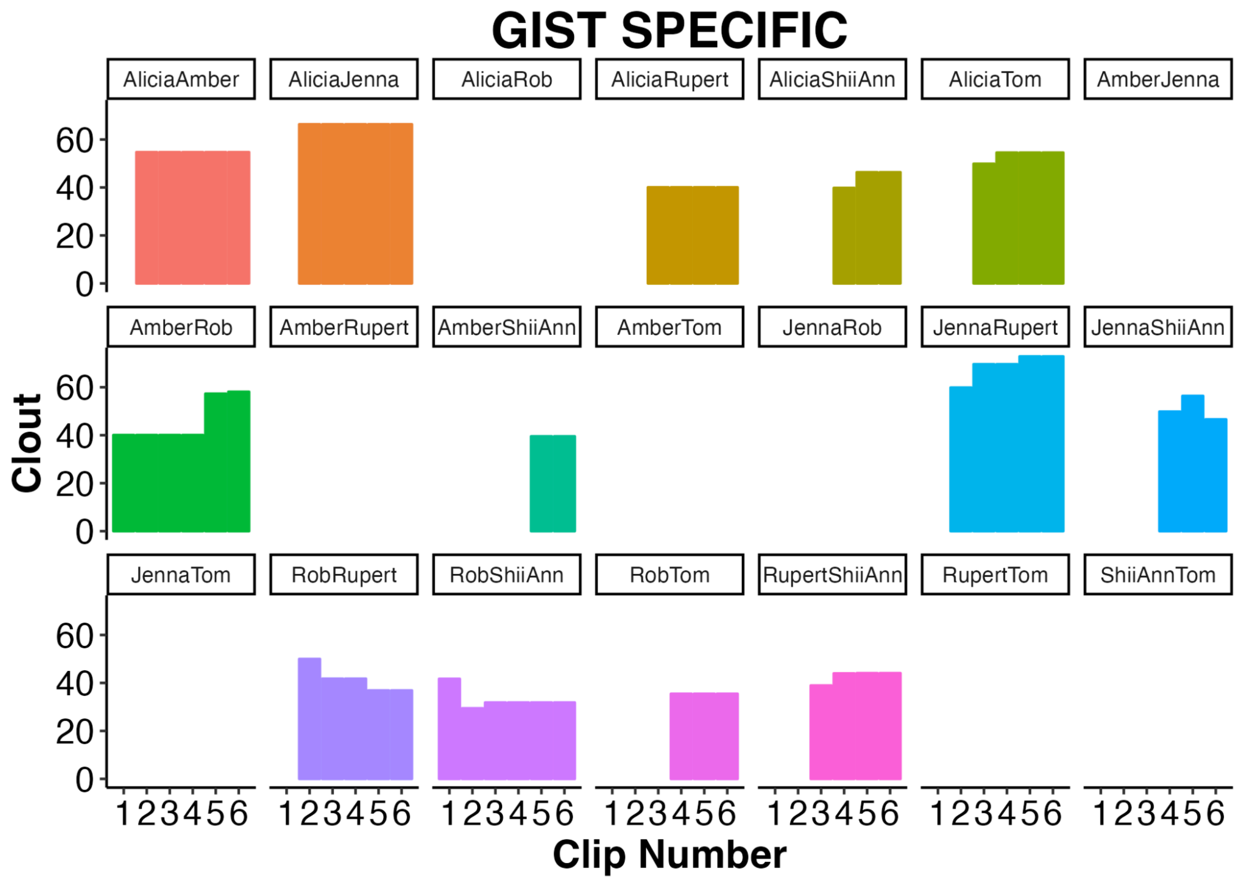

Supp. Fig. 30. Dyadic gist specific clout across episode clips. Average gist specific clout per dyad across episode clips. Clout scores range from 1 to 99.

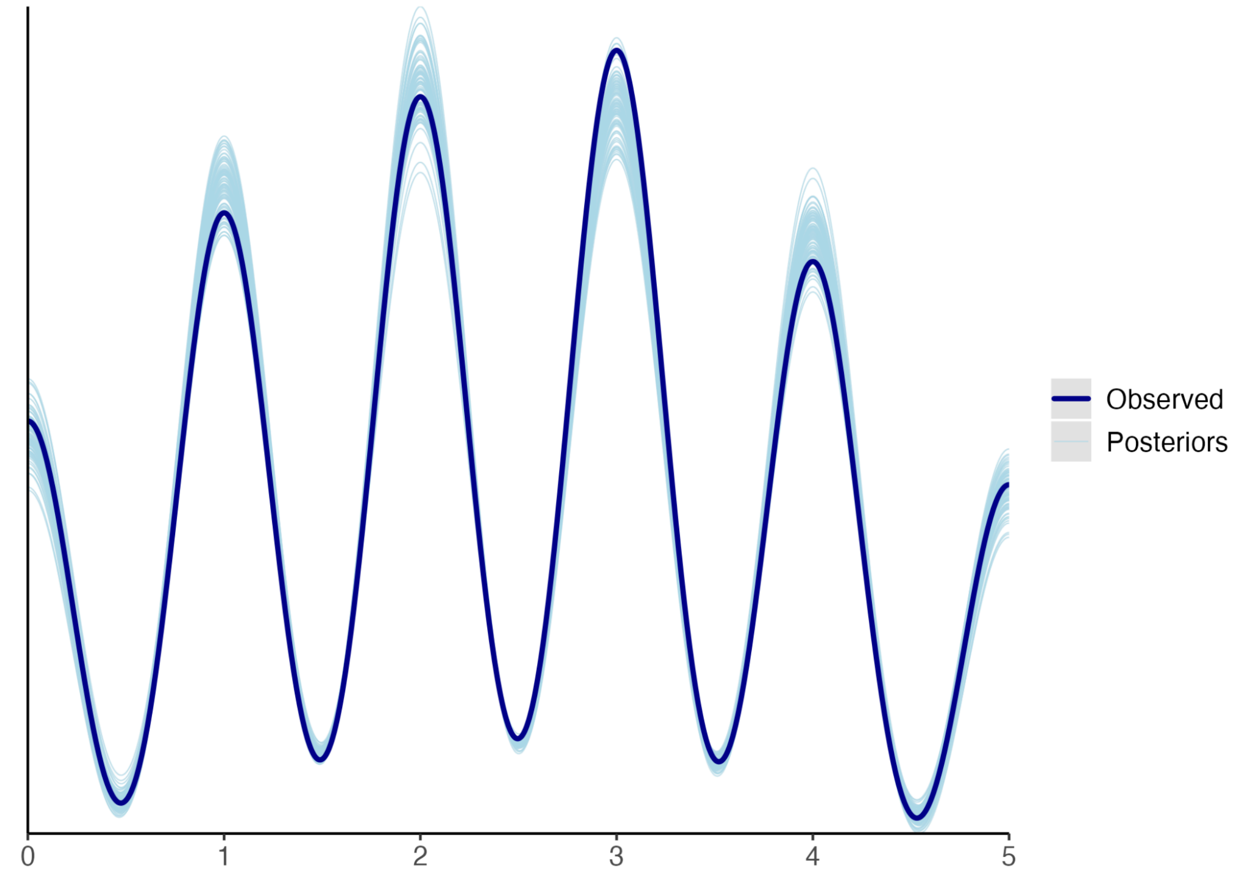

Supp. Fig. 31. Posterior predictive check density plot for gist specific clout model. Dark blue line shows observed data and light blue lines reflect a sample of 100 posteriors. Due to the ratio of selected to presented responses used to calculate % time chosen, a beta-binomial model with logit link was fit to the data. The waveform pattern of observed data reflects the ratio of integers of selected vs. presented responses. This graph highlights that the simulated data from model posteriors (light blue lines) are an excellent fit to the pattern in the observed data (dark blue line).

Recent Specific

Supp. Table 19. Recent specific clout. Model estimates and 95% credibility intervals shown for fixed effects. MCMC chain convergence (Rhat) and chain resolution (bulk effective sample size) values shown for fixed effects.

	Estimate	95% CrI [lower, upper]	Rhat	Bulk Effective Sample Size
Intercept	-0.339	[-0.778, 0.097]	1.00137	4089
Recent Specific Clout	0.009	[0.006, 0.012]	1.00004	36568
Condition (Ref. = Friend)	0.494	[-0.238, 1.224]	1.00154	4167
Recent Specific Clout * Condition (Ref. = Friend)	-0.013	[-0.017, -0.008]	0.99999	44809

Supp. Table 20. Recent specific clout marginal effects. Marginal effects for friend and rival conditions and 95% credibility intervals shown.

Condition	Marginal Effect	95% CrI [lower, upper]
Friend	0.009	[0.006, 0.012]
Rival	-0.003	[-0.006, -0.0003]

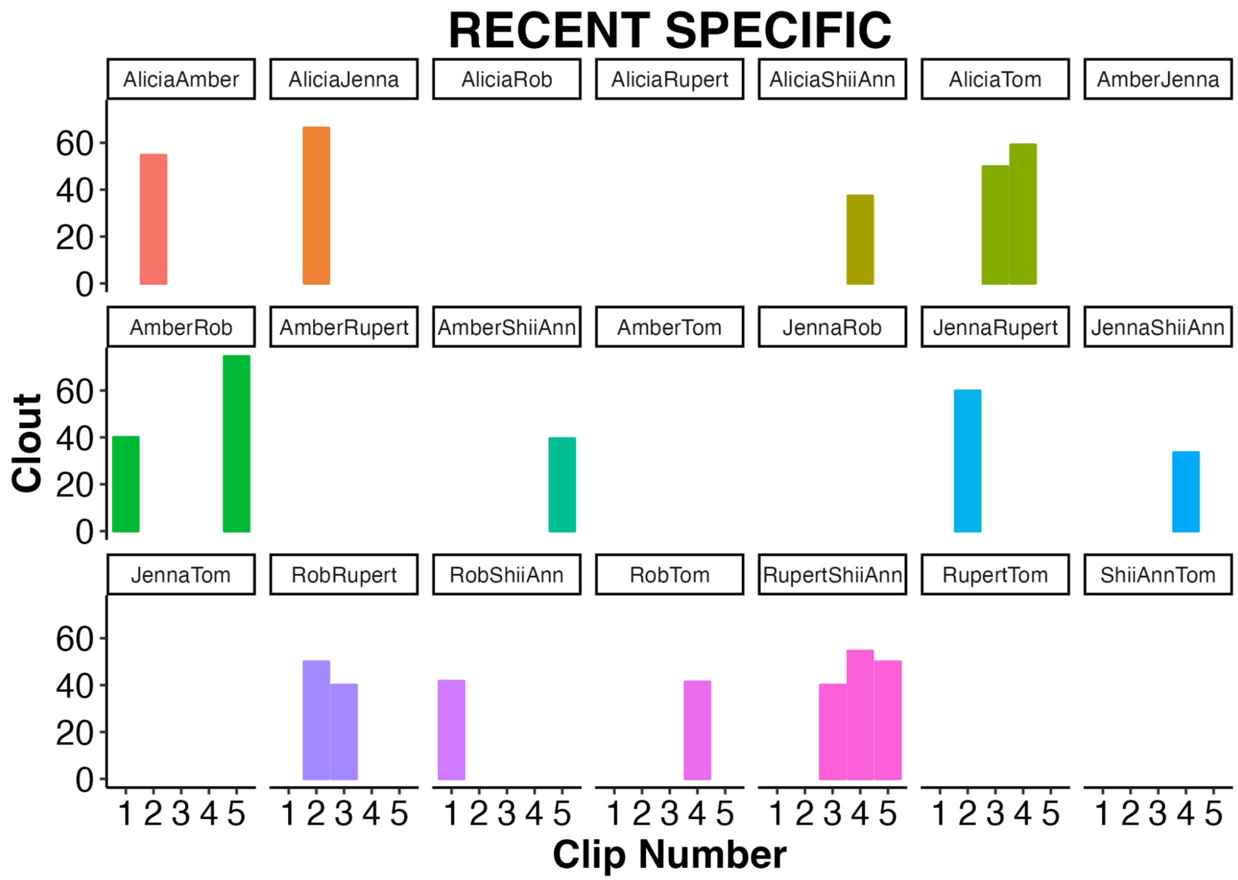

Supp. Fig. 32. Dyadic recent specific clout across episode clips. Average recent specific clout per dyad across episode clips. Clout scores range from 1 to 99.

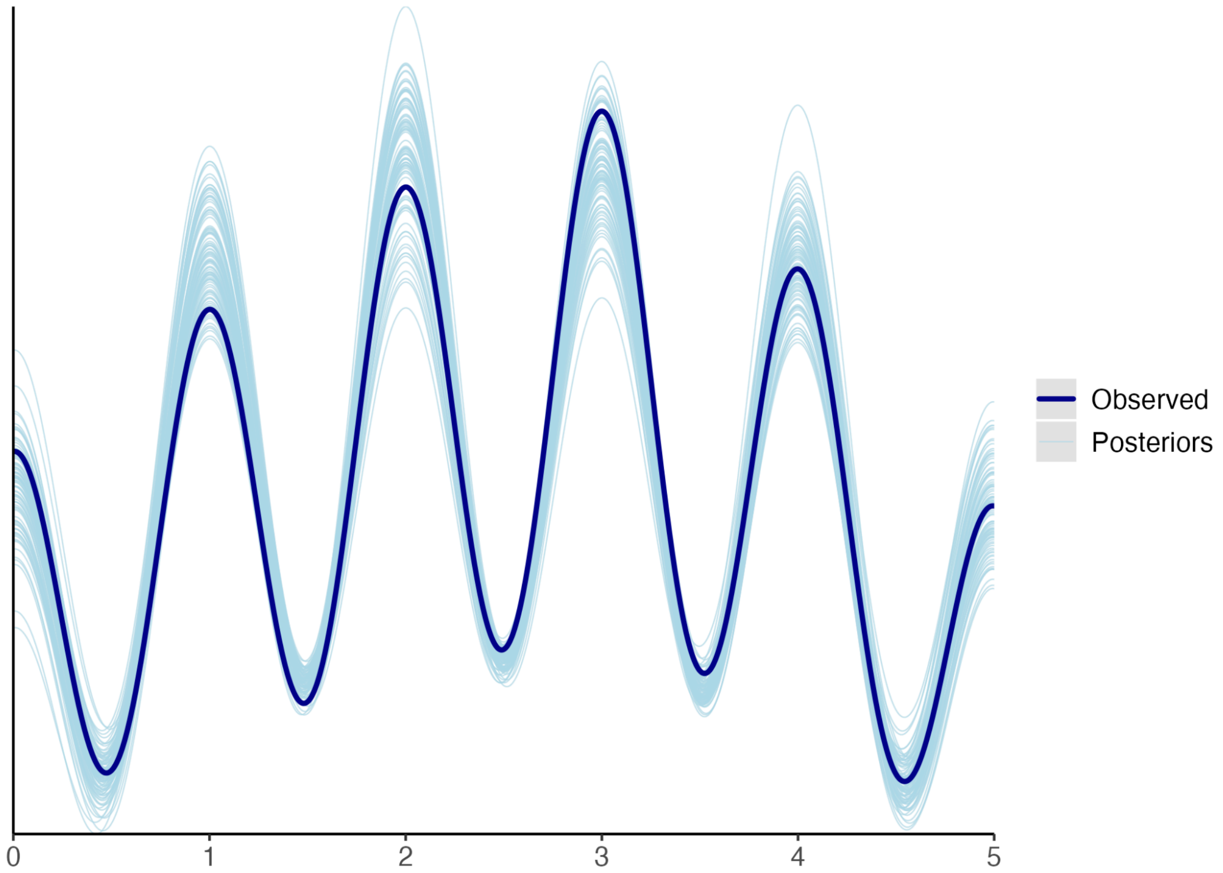

Supp. Fig. 33. Posterior predictive check density plot for recent specific clout model. Dark blue line shows observed data and light blue lines reflect a sample of 100 posteriors. Due to the ratio of selected to presented responses used to calculate % time chosen, a beta-binomial model with logit link was fit to the data. The waveform pattern of observed data reflects the ratio of integers of selected vs. presented responses. This graph highlights that the simulated data from model posteriors (light blue lines) are an excellent fit to the pattern in the observed data (dark blue line).

Gist General

Supp. Table 21. Gist general clout. Model estimates and 95% credibility intervals shown for fixed effects. MCMC chain convergence (Rhat) and chain resolution (bulk effective sample size) values shown for fixed effects.

	**Estimate**	**95% CrI [lower, upper]**	**Rhat**	**Bulk Effective Sample Size**
Intercept	0.014	[-0.321, 0.347]	1.00124	3789
Gist General Clout	-0.0003	[-0.003, 0.003]	1.00003	55677
Condition (Ref. = Friend)	-0.237	[-0.801, 0.328]	1.00118	3682
Gist General Clout * Condition (Ref. = Friend)	0.005	[0.0002, 0.009]	1.00013	68215

Supp. Table 22. Gist general clout marginal effects. Marginal effects for friend and rival conditions and 95% credibility intervals shown.

**Condition**	**Marginal Effect**	**95% CrI [lower, upper]**
Friend	-0.0003	[-0.003, 0.003]
Rival	0.005	[0.001, 0.008]

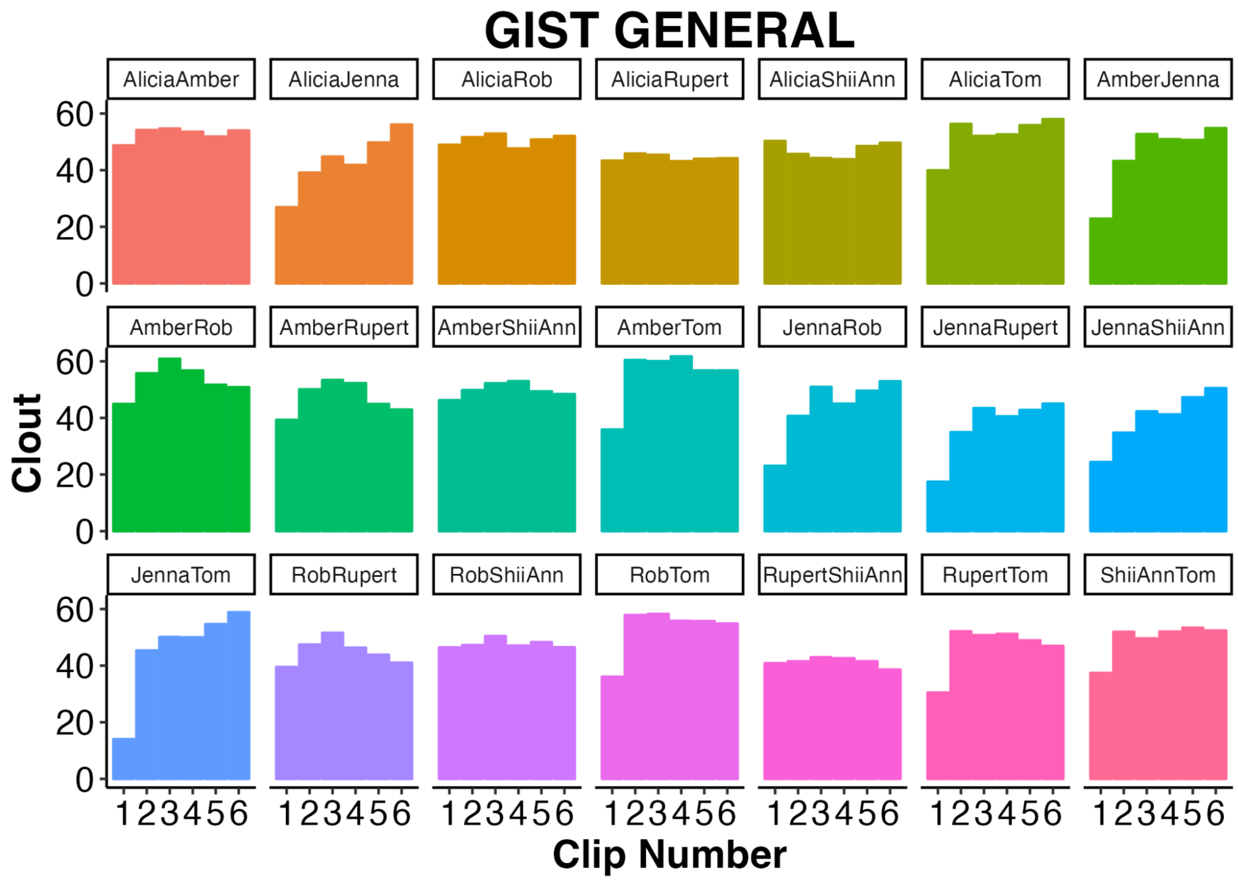

Supp. Fig. 34. Dyadic gist general clout across episode clips. Average gist general clout per dyad across episode clips. Clout scores range from 1 to 99.

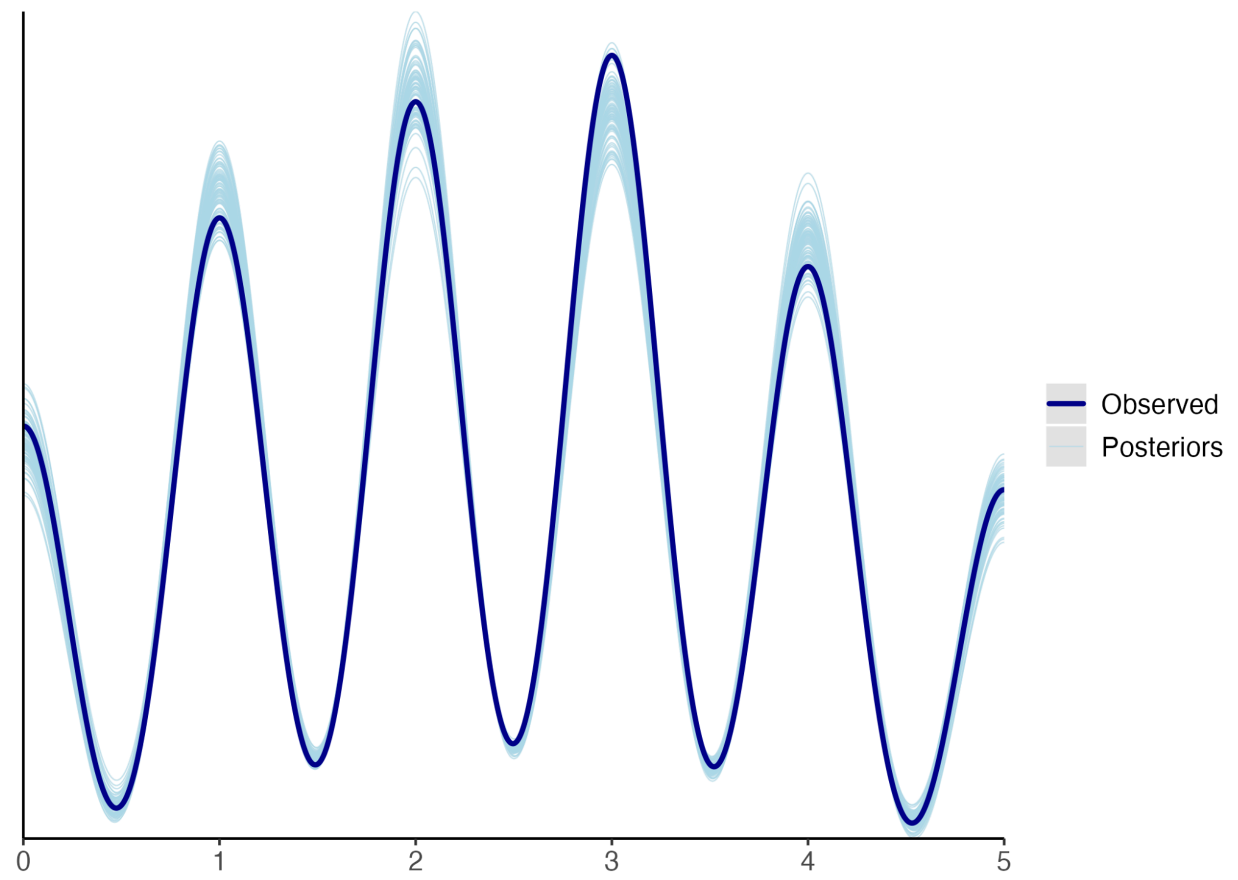

Supp. Fig. 35. Posterior predictive check density plot for gist general clout model. Dark blue line shows observed data and light blue lines reflect a sample of 100 posteriors. Due to the ratio of selected to presented responses used to calculate % time chosen, a beta-binomial model with logit link was fit to the data. The waveform pattern of observed data reflects the ratio of integers of selected vs. presented responses. This graph highlights that the simulated data from model posteriors (light blue lines) are an excellent fit to the pattern in the observed data (dark blue line).

Recent General

Supp. Table 23. Recent general clout. Model estimates and 95% credibility intervals shown for fixed effects. MCMC chain convergence (Rhat) and chain resolution (bulk effective sample size) values shown for fixed effects.

	**Estimate**	**95% CrI [lower, upper]**	**Rhat**	**Bulk Effective Sample Size**
Intercept	-0.044	[-0.343, 0.256]	1.00075	3444
Clout Recent General	0.001	[-0.001, 0.003]	1.00006	43755
Condition (Ref. = Friend)	-0.073	[-0.598, 0.449]	1.00064	3500
Clout Recent General * Condition (Ref. = Friend)	0.001	[-0.002, 0.003]	1.00015	47738

Supp. Table 24. Recent general clout marginal effects. Marginal effects for friend and rival conditions and 95% credibility intervals shown.

**Condition**	**Marginal Effect**	**95% CrI [lower, upper]**
Friend	0.001	[-0.0005, 0.003]
Rival	0.002	[0.00005, 0.003]

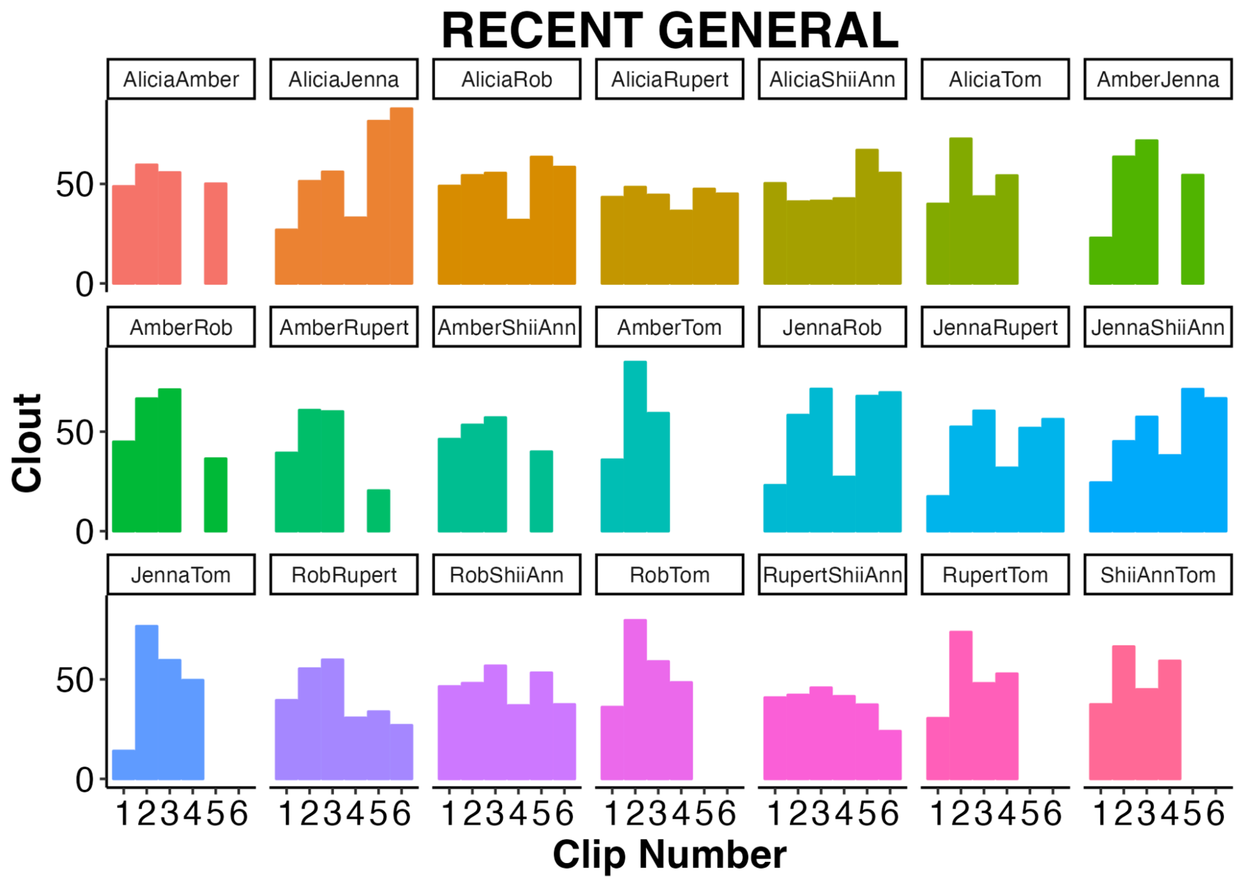

Supp. Fig. 36. Dyadic recent general clout across episode clips. Average recent general clout per dyad across episode clips. Clout scores range from 1 to 99.

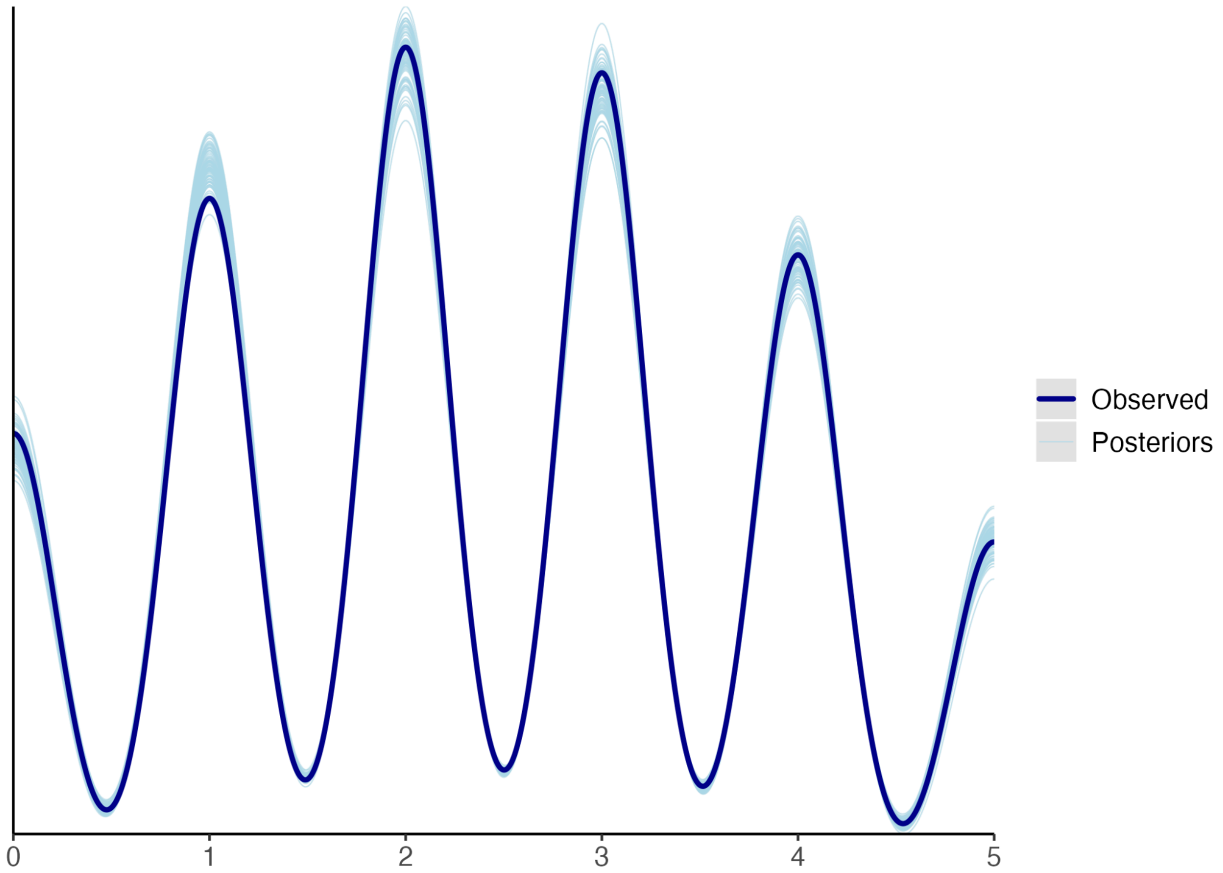

Supp. Fig. 37. Posterior predictive check density plot for recent general clout model. Dark blue line shows observed data and light blue lines reflect a sample of 100 posteriors. Due to the ratio of selected to presented responses used to calculate % time chosen, a beta-binomial model with logit link was fit to the data. The waveform pattern of observed data reflects the ratio of integers of selected vs. presented responses. This graph highlights that the simulated data from model posteriors (light blue lines) are an excellent fit to the pattern in the observed data (dark blue line).

LASSO Regression – Combined Linguistic Features

In addition to distinct evaluations of the associations between semantic similarity, sentiment, and clout and social-relational inference, we investigated all features together at each level of analysis. We employed Least Absolute Shrinkage and Selection Operator (LASSO) regression (Ranstam & Cook, 2018; Tibshirani, 1996) using the "glmnet" package in R (Friedman et al., 2010). LASSO regression is typically used to identify variables that minimize model prediction error (Ranstam & Cook, 2018). We fit four LASSO models for each level of analysis (recent-general, recent-specific, gist-general, gist-specific) to examine overlapping contributions of each linguistic feature on relational judgments.

We created four data frames that included relational judgments per condition and language feature scores (semantic similarity, sentiment, clout) per level of analysis. This allowed us to appropriately remove any missing values that corresponded to missed behavioral responses or that reflected the lack of interaction between a given dyad. To account for within-participant differences between friendship and rivalry judgments, we calculated a participant friend – rival % chosen difference score for each dyad.

For each regression, we also calculated an optimal lambda using k-fold cross validation to determine which lambda value best minimized prediction error. We used this optimal lambda in a LASSO regression model that included participant % chosen difference scores as the outcome variable and dyad, semantic similarity, sentiment, and clout as predictors. We obtained estimate coefficients for each predictor and calculated an R^2 for each model to measure the strength of the modeled relationship between relational judgment differences and all language features. Least associated predictors are decreased to zero, leaving only predictors that are meaningful predictors in the model.

Supp. Table 25. Recent general model. Model estimates and shown for fixed effects. Optimal lambda (λ) minimizes test mean squared error and R^2 shows the amount of variance explained in relational judgments by the best model.

	**Estimate**	**Optimal λ**	**R^2**
Intercept	0.1206	0.000197	0.027117
Dyad	-0.0011		
Sentiment	-0.1111		
Clout	0.0004		
Semantic Similarity	-0.4104		

Supp. Table 26. Recent specific model. Model estimates and shown for fixed effects. Optimal lambda (λ) minimizes test mean squared error and R^2 shows the amount of variance explained in relational judgments by the best model.

	Estimate	Optimal λ	R^2
Intercept	-0.0331		
Dyad	-0.0034		
Sentiment	-0.3212	0.000302	0.066853
Clout	0.0026		
Semantic Similarity	-0.1206		

Supp. Table 27. Gist general model. Model estimates and shown for fixed effects. Optimal lambda (λ) minimizes test mean squared error and R^2 shows the amount of variance explained in relational judgments by the best model.

	Estimate	Optimal λ	R^2
Intercept	0.2387		
Dyad	-0.0039		
Sentiment	-0.4975	0.000181	0.042346
Clout	0.0026		
Semantic Similarity	-0.8643		

Supp. Table 28. Gist specific model. Model estimates and shown for fixed effects. Optimal lambda (λ) minimizes test mean squared error and R^2 shows the amount of variance explained in relational judgments by the best model.

	Estimate	Optimal λ	R^2
Intercept	0.0304		
Dyad	-0.0024		
Sentiment	-0.3245	0.000274	0.065805
Clout	0.0018		
Semantic Similarity	-0.3580		

Language Similarity and Relational Homophily

General

Supp. Table 29. General early semantic similarity. Model estimates and 95% credibility intervals shown for fixed effects. MCMC chain convergence (Rhat) and chain resolution (bulk effective sample size) values shown for fixed effects.

	Estimate	95% CrI [lower, upper]	Rhat	Bulk Effective Sample Size
Intercept	-1.419	[-2.927, 0.171]	1.00045	5831
General Semantic Similarity	3.026	[-0.296, 6.164]	1.00039	6156
Condition (Ref. = Friend)	1.600	[-1.193, 4.276]	1.00050	6161
General Semantic Similarity * Condition (Ref. = Friend)	-3.422	[-8.975, 2.437]	1.00055	6432

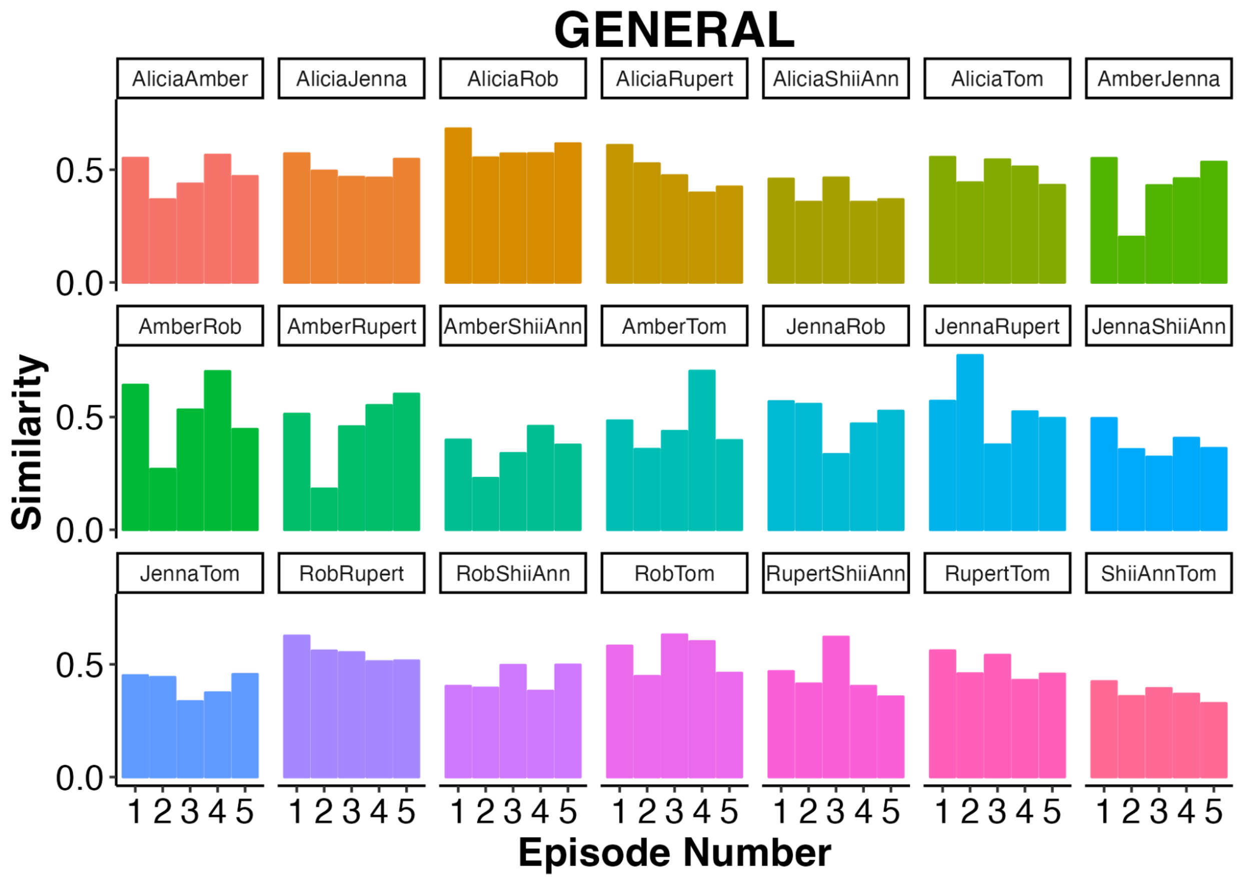

Supp. Fig. 38. Dyadic general semantic similarity in early episodes. Average general semantic similarity per dyad across episode numbers. Similarity scores range from -1 to 1.

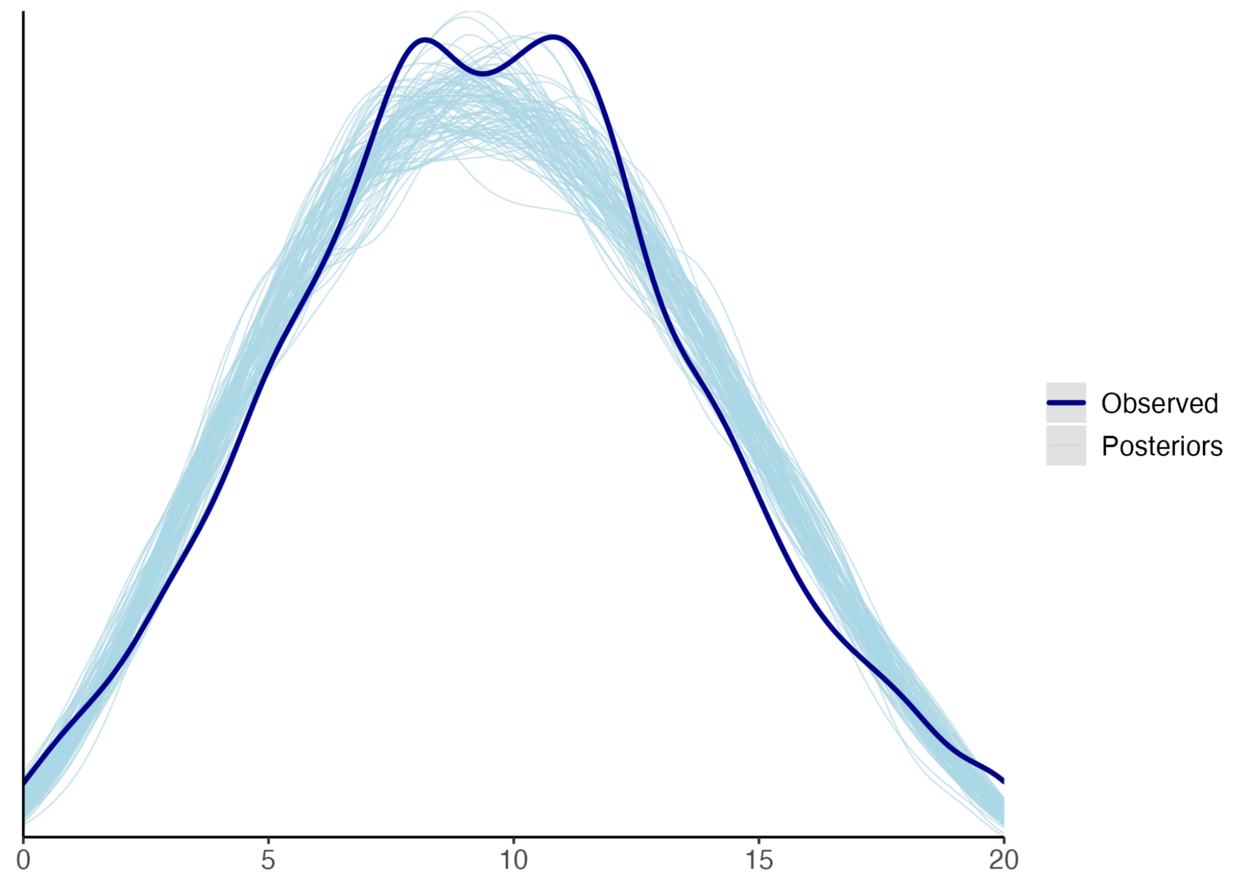

Supp. Fig. 39. Posterior predictive check density plot for general early semantic similarity model. Dark blue line shows observed data and light blue lines reflect a sample of 100 posteriors. This graph highlights that the simulated data from model posteriors (light blue lines) are an excellent fit to the pattern in the observed data (dark blue line).

Specific

Supp. Table 30. Specific early semantic similarity. Model estimates and 95% credibility intervals shown for fixed effects. MCMC chain convergence (Rhat) and chain resolution (bulk effective sample size) values shown for fixed effects.

	Estimate	95% CrI [lower, upper]	Rhat	Bulk Effective Sample Size
Intercept	0.080	[-0.496, 0.663]	1.00038	10280
Specific Semantic Similarity	0.174	[-2.496, 2.816]	1.00039	12833
Condition (Ref. = Friend)	-0.131	[-1.175, 0.921]	1.00038	10357
Specific Semantic Similarity * Condition (Ref. = Friend)	-0.403	[-5.151, 4.438]	1.00039	12999

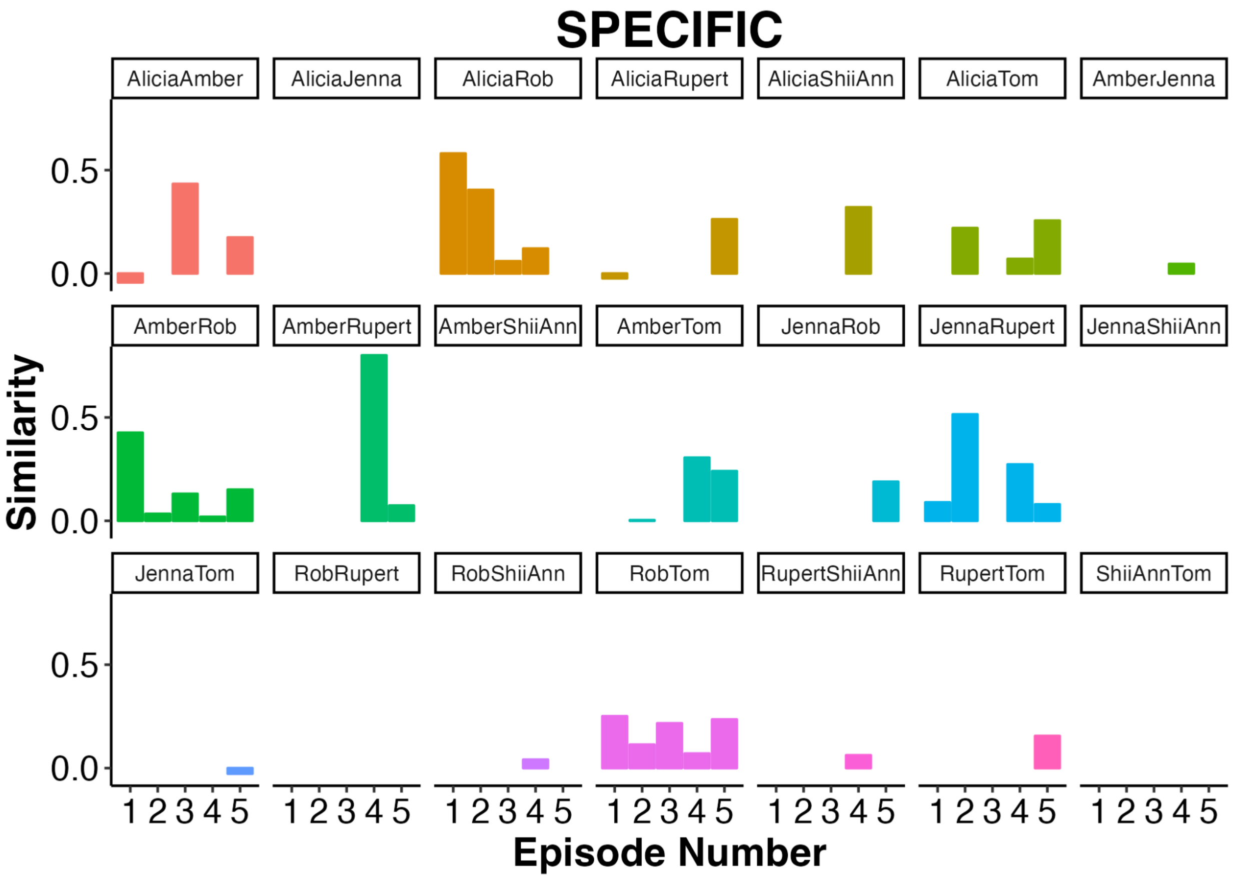

Supp. Fig. 40. Dyadic specific semantic similarity in early episodes. Average specific semantic similarity per dyad across episode numbers. Similarity scores range from -1 to 1.

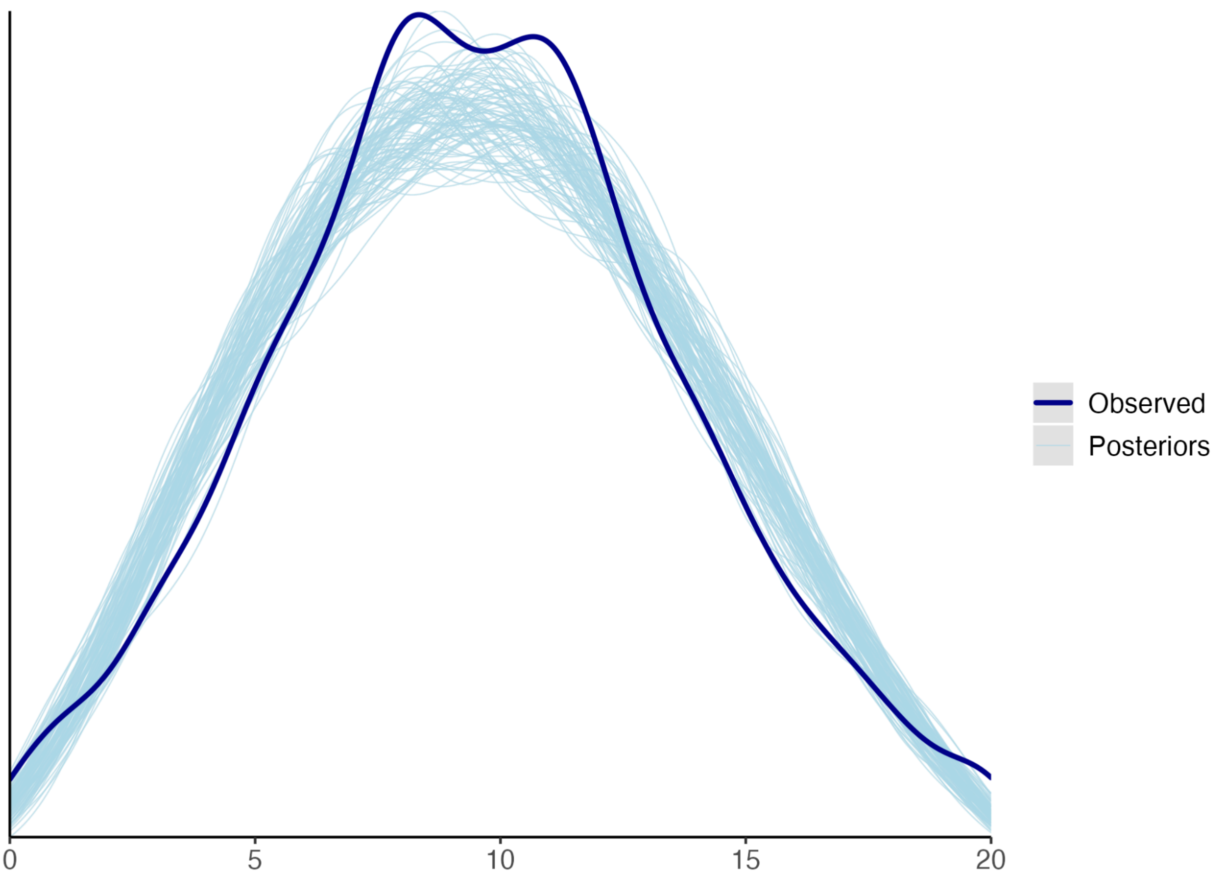

Supp. Fig. 41. Posterior predictive check density plot for specific early semantic similarity model. Dark blue line shows observed data and light blue lines reflect a sample of 100 posteriors. This graph highlights that the model posteriors (light blue lines) are an excellent fit to the pattern in the observed data (dark blue line).

Supplemental References

Friedman, J. H., Hastie, T., & Tibshirani, R. (2010). Regularization Paths for Generalized Linear Models via Coordinate Descent. *Journal of Statistical Software*, *33*, 1–22. https://doi.org/10.18637/jss.v033.i01

Ranstam, J., & Cook, J. A. (2018). LASSO regression. *The British Journal of Surgery*, *105*(10), 1348–1348. https://doi.org/10.1002/bjs.10895

Tibshirani, R. (1996). Regression Shrinkage and Selection Via the Lasso. *Journal of the Royal Statistical Society. Series B, Statistical Methodology*, *58*(1), 267–288. https://doi.org/10.1111/j.2517-6161.1996.tb02080.x
